# Asynchronous temporal mapping for high-dynamic-range video and hardware-level privacy with event cameras

**DOI:** 10.1038/s44172-026-00687-4

**Published:** 2026-05-13

**Authors:** Yuhan Bao, Hao Shi, Jiajun Zhai, Kaiwei Wang

**Affiliations:** 1 State Key Laboratory of Extreme Photonics and Instrumentation, Zhejiang University, Hangzhou, China; 2 College of Optical Science and Engineering, Zhejiang University, Hangzhou, China; 3 Central Research Institute of Sunny Optical Technology, Hangzhou, China

**Keywords:** Imaging and sensing, Imaging techniques

## Abstract

Conventional imaging systems struggle to simultaneously achieve high-dynamic-range imaging under extreme illumination and ensure that visual data remain confidential from the moment of acquisition. Here we present Asynchronous Temporal Mapping, a framework that encodes high-dynamic-range visual information into a chaotic, asynchronous event stream through pixel-wise optical modulation. The framework addresses the bandwidth bottleneck of prior synchronous temporal-mapping approaches, enabling high-fidelity video capture with event cameras. By embedding the asynchronous principle of event sensing into a computational optical pipeline, it introduces an imaging framework characterized by high-dynamic-range video capture and inherent hardware-level protection.

## Abstract (web summary)

Yuhan Bao and colleagues propose an asynchronous sparse imaging system that enables both HDR video capture and real-time visual encryption. By asynchronously modulating the transmittance in front of event-camera pixels, the system securely maps visual information into the time domain, providing a hardware foundation for future privacy preserving and efficient visual sensing.

## Introduction

Faithfully capturing reality—preserving fine textures in both extreme highlights and deep shadows—stands as a fundamental requirement for vision systems in fields ranging from autonomous driving to medical diagnostics. However, conventional cameras inevitably fail under such challenging conditions due to their inherent limited dynamic range, resulting in saturated highlights and noise-corrupted shadows. Simultaneously, growing concerns over privacy demand that imaging systems protect visual data from the moment of capture. In safety-critical environments and privacy-sensitive public surveillance, there is a pressing need to minimize plaintext exposure and avoid the latency of software-based encryption. This calls for a camera that serves as a confidential eye capable of faithfully perceiving reality while providing inherent privacy protection at the point of capture.

Event cameras present a promising pathway toward overcoming these dual challenges by leveraging their unique operating principles. For capturing reality, their inherent high dynamic range (HDR) enables the fine detail capture in both extremely dark and bright regions under challenging illumination^1^. However, their reliance on temporal brightness contrast creates an inherently sparse output that fails to capture static or low-texture regions, leading to substantial information loss in stationary scenes and preventing them from truly capturing reality. To overcome this inherent limitation, existing approaches have mainly followed two directions: some works fuse events with additional modalities like grayscale frames to fill the information gap^2–4^, while others seek more self-contained solutions within the event sensing paradigm. Among the latter, methods that reconstruct scenes solely from brightness changes—such as event-based video reconstruction networks^5–7^—produce only pseudo-grayscale outputs that fail to faithfully represent the true scene irradiance. For confidential vision, the fundamental operation of event cameras—recording only relative brightness changes while omitting absolute intensity—provides a native form of information obfuscation, seemingly offering inherent privacy protection at the hardware level. However, while absolute intensity is concealed, the accumulated spatiotemporal event patterns still reveal sufficient structural and contour information, leading to potential information leakage that remains inadequate for high-stakes confidential applications.

Recent advances in event-based computational optical imaging^8–12^ have demonstrated that active modulation, which controls the optical process to encode scene information into the event stream, can unlock novel sensing modalities. This enables event cameras to capture richer optical information far beyond the mere brightness changes induced by scene motion. Among them, temporal-mapping approaches^13,14^ attempt to overcome the intensity ambiguity of event cameras by globally modulating optical transmittance and decoding absolute intensity from event timing, thereby enabling high-fidelity snapshot HDR imaging. However, their synchronous, global modulation schemes suffer from a fundamental bottleneck for video acquisition: at the high modulation rates required for video, it triggers near-simultaneous events across the entire sensor. This creates bursty event explosions that saturate the sensor’s data bandwidth, leading to severe readout artifacts and fundamentally restricting its application to low-frame-rate snapshot imaging. Moreover, the deterministic link between event timing and scene intensity offers no inherent privacy protection, as the raw event stream remains visually interpretable. Thus, while temporal mapping provides a path to high-fidelity intensity recovery, its synchronous implementation is ill-suited for confidential, high-speed video capture.

**Fig. 1:**
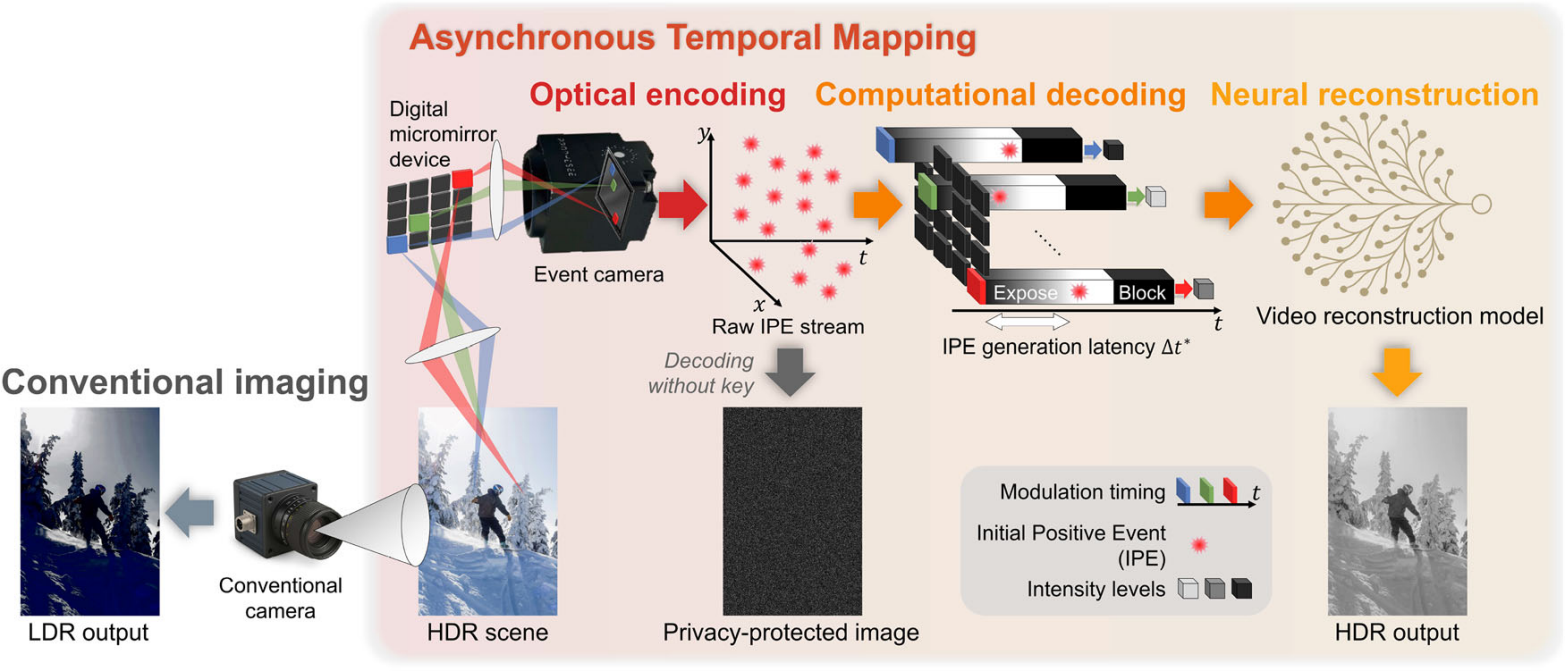
Schematic of the Asynchronous Temporal Mapping (AsynTemMap) pipeline, which achieves simultaneous privacy protection and high-dynamic-range (HDR) reality capture in a unified framework. Pixel-wise modulation by the digital micromirror device (DMD) optically encodes HDR scene intensities into the generation latencies of Initial Positive Events (IPEs), producing a chaotic raw IPE stream which, when decoded without the key, results in only noise-like outputs, thus preserving privacy at the point of acquisition. The computational decoding stage computes each IPE's generation latency Δt^*^ from its modulation onset timing, then maps latency to intensity. These intensities are then passed to a neural reconstruction model, which restores a photorealistic HDR output with preserved details in shadows and highlights. A conventional camera image is shown for comparison, where low dynamic range (LDR) causes loss of shadow detail. Background photograph by Greg Rosenke on Unsplash.

To simultaneously achieve high-dynamic-range video acquisition and inherent privacy protection with an event camera, we propose Asynchronous Temporal Mapping (AsynTemMap). As illustrated in Fig. 1, our framework fully leverages the asynchronous sensing characteristics of event cameras to establish a computational optical imaging system that integrates optical encoding, computational decoding, and neural reconstruction. Specifically, AsynTemMap applies pixel-wise asynchronous transmittance modulation to an event camera, encoding the absolute intensity information of reality into the generation latency Δt* of each initial positive event (IPE). This optical encoding not only ensures a stable data flow that supports high-speed video acquisition but also transforms sensitive visual information into a chaotic asynchronous IPE distribution. At the earliest stage of visual sensing, this process inherently encrypts the captured data, eliminating the exposure window for software encryption and removing the encryption time overhead, providing zero-latency confidentiality at the point of capture. Once each IPE is associated with the correct modulation timing, the computational decoding process recovers the scene’s intensity by applying temporal mapping principles, converting each IPE into an intensity level. Subsequently, a neural reconstruction model integrates these asynchronous intensity levels, mitigating asynchronous sampling artifacts and ultimately reconstructing HDR video frames that faithfully restore both highlight and shadow details of reality. Building on this system, we have made temporal mapping technology truly applicable to video-rate visual information capture, overcoming its original limitation to low-frame-rate snapshot imaging while preserving its ability to capture reality in low-light and HDR scenes. Extensive experiments in dynamic motion scenarios demonstrate the versatility of AsynTemMap. More importantly, we provide compelling evidence of its inherent privacy protection in diverse privacy-sensitive contexts. Unlike traditional software-based event encryption algorithms, AsynTemMap eliminates encryption overhead and reduces decryption time by a factor of 200, thereby ensuring real-time privacy-preserving data acquisition at the point of capture, while subsequent neural reconstruction is performed offline for high-quality recovery. Additionally, it enhances encryption security, as reflected in improved key sensitivity and ciphertext randomness.

In summary, our work bridges the gap between high-fidelity intensity recovery and practical high-speed acquisition in event-based vision. The key contributions are: (i) A paradigm of high-fidelity video acquisition for event cameras: We introduce the Asynchronous Temporal Mapping (AsynTemMap) paradigm. By replacing global synchronous modulation with pixel-wise asynchronous modulation, we overcome the bandwidth saturation that plagues existing temporal-mapping methods, enabling stable, high-frame-rate video acquisition while preserving the ability to faithfully recover absolute scene irradiance. (ii) A unified reconstruction pipeline: We develop an end-to-end pipeline comprising computational decoding and a neural reconstruction model (AsynTemRec). This pipeline jointly compensates for the temporal misalignment artifacts induced by asynchronous sampling and various hardware imperfections, delivering high-fidelity video reconstruction across diverse dynamic scenes, including those with extreme illumination. (iii) Hardware-level confidential vision: We demonstrate that the asynchronous modulation mechanism inherently implements a Secure Event Coding via Asynchronous Modulation (SECAM) scheme. This provides robust, zero-latency privacy protection at the point of capture, with security metrics rivaling software encryption but at a fraction of the computational cost.

## Principle and system overview

### Temporal-mapping principle: latency to intensity

The principle of temporal mapping is based on modulating the optical transmittance of the imaging system, thereby inducing controlled event generation whose latency is directly linked to the scene’s true absolute light intensity. As the transmittance *TR*(*t*) ramps from 0, the incident intensity at each pixel (*x*, *y*) varies as $I(x,y,t)={I}_{\max }(x,y)\cdot TR(t),$ where ${I}_{\max }(x,y)$ represents the absolute scene intensity. When the accumulated energy reaches the threshold *E*_thd_, the pixel emits an initial positive event (IPE). The response latency Δt*(*x*, *y*) of IPE–measured from the onset of modulation–therefore directly encodes the absolute intensity, as expressed by

1 $${I}_{\max }(x,y)=\frac{{E}_{thd}}{{\int }_{\!\!0}^{\Delta {t}^{* }(x,y)}TR(t)\,dt}.$$

This establishes an explicit latency–intensity mapping, which forms the physical foundation of temporal-mapping imaging and enables the capture of high-fidelity HDR grayscale snapshots using a single event camera. This capability enables the simultaneous and precise capture of details in both highlights and shadows under extreme illumination. The temporal-mapping imaging principle has also been successfully applied across diverse domains, including HDR low-light vision^15^, fluorescence microscopy^16^, 3D reconstruction^17^, and focus estimation^18^. However, while the synchronous global modulation scheme employed in temporal mapping’s original setup adequately supports snapshot imaging at low frame rates, it encounters a fundamental bottleneck at higher modulation frequencies. The near-simultaneous triggering of events across the entire sensor creates intense data bursts that impose excessive bandwidth pressure. This fundamental bandwidth bottleneck ultimately prevents synchronous temporal mapping from being used for continuous video acquisition, which motivates the proposed asynchronous approach.

### Pixel-wise asynchronous modulation

To enable video acquisition by overcoming the bandwidth bottleneck of synchronous modulation, we introduce pixel-wise asynchronous modulation. As illustrated in Fig. 1, the asynchronous transmittance modulation is implemented by a digital micromirror device (DMD). Each micromirror periodically flips between on and off states. When a micromirror turns to the on state, the incident light is reflected toward the corresponding pixel on the event camera. During the period in which the micromirror remains on, the pixel integrates incoming light, and an IPE is triggered at a specific latency Δt* after the modulation onset, depending on the local illumination intensity. Subsequently, the micromirror flips to the off state, blocking the light and resetting the pixel for the next acquisition.

Each micromirror follows a distinct flipping schedule, introducing deliberate temporal offsets among pixels so that the modulation onsets are spatially desynchronized. This staggered modulation timing effectively disperses the IPE generation across the modulation period, preventing simultaneous bursts of events and thereby relieving the bandwidth pressure that occurs under global modulation. Through this mechanism, AsynTemMap preserves the inherent high-fidelity imaging capability of temporal mapping while extending it toward stable and continuous video acquisition.

### Secure event coding via asynchronous modulation

Notably, the asynchronous modulation mechanism of AsynTemMap inherently provides a zero-latency, hardware-level visual encryption capability, termed Secure Event Coding via Asynchronous Modulation (SECAM). This addresses the critical need for inherent privacy protection at the capture stage, eliminating the plaintext exposure window present in software encryption.

In AsynTemMap, the temporal offsets *O*(*i*, *j*), which define the flipping schedule of each micromirror, serve as the encryption key. Under the asynchronous modulation, the onsets of pixel-wise exposure are deliberately scrambled. As a result, the raw IPE stream captured by the event camera appears chaotic, as shown in Fig. 1. The absolute timestamp of each IPE loses its direct correspondence to scene intensity, causing any direct temporal-mapping decoding attempt to yield only noise-like output. This ensures privacy protection from the very moment of data acquisition. Authorized decryption requires subtracting the respective modulation onset from the timestamp of each raw IPE, thereby recovering its true generation latency Δt*. Applying the mapping in Eq. (1) then converts these latencies into intensity, completing the computational decoding from raw IPE stream to raw AsynTemMap frames. This hardware-level encryption paradigm provides robust privacy protection while enabling real-time processing.

### Reconstruction under motion and hardware degradation

Each pixel in the decrypted raw AsynTemMap frames reflects the scene intensity sampled at a distinct temporal instant across the sensor array. In static or slowly varying scenes, this is equivalent to a conventional image. However, when the scene undergoes rapid motion, the asynchronous sampling among pixels introduces feature misalignment within moving regions, producing asynchronous sampling artifacts (ASA). In addition, real hardware systems inevitably exhibit imperfections—including optical aberrations, DMD defects, and sensor noise—which further degrade the image fidelity.

To mitigate these issues, we develop a neural AsynTemMap Video Reconstruction (AsynTemRec) model that jointly compensates for ASA and hardware-induced degradation. Through this hybrid optical–computational pipeline, AsynTemMap achieves high-fidelity video reconstruction under dynamic scenes and imperfect devices, faithfully capturing reality. The overall performance of the AsynTemMap system—including its reconstruction fidelity and robustness under motion and hardware degradation—is comprehensively validated.

## Result

We comprehensively evaluate the performance of the proposed AsynTemMap system through a series of experiments covering static, dynamic, and privacy-sensitive imaging scenarios. The experiments are designed to validate its capabilities in four key aspects: (i) high-dynamic-range imaging in extreme illumination conditions, (ii) stable high-speed video acquisition enabled by asynchronous modulation, (iii) robust reconstruction in the presence of motion and hardware degradation, and (iv) inherent privacy protection at capture via the SECAM mechanism and its quantitative advantage over conventional software encryption.

### From snapshot to video: overcoming the bandwidth bottleneck

#### High-fidelity snapshot imaging via synchronous modulation

**Fig. 2:**
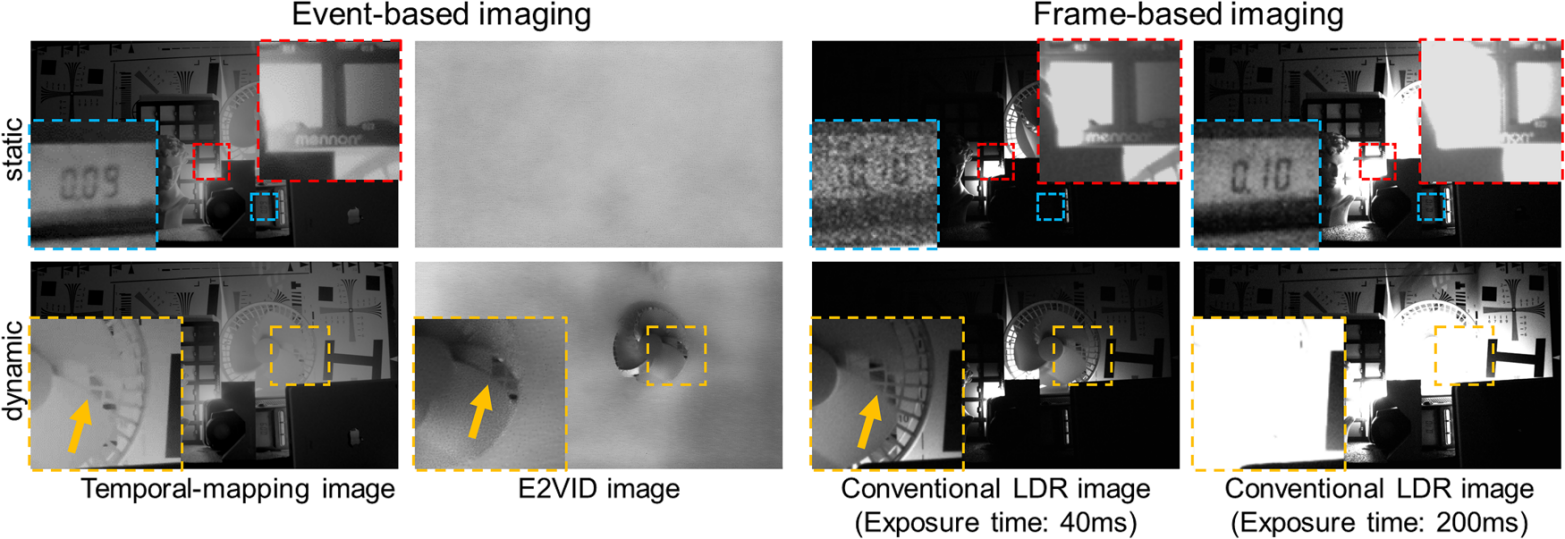
Comparison of HDR imaging performance under extreme illumination conditions using different imaging methods. In static scenes, temporal-mapping imaging under synchronous modulation captures the brightly lit text mennon on the color chart (red box) alongside the dimly lit digital reading 0.09 on the illuminance meter (blue box). E2VID^5^ produces no recognizable output. Conventional frame-based imaging fails to preserve both bright and dark details under any single exposure setting. In dynamic scenes with a rotating fan, temporal-mapping maintains clear features with minimal motion artifacts. E2VID recovers the general fan shape but with apparent intensity distortion. Conventional imaging exhibits noticeable motion blur in the fan region. Regions within boxes have been adjusted in grayscale contrast and brightness for optimal visualization.

To evaluate imaging performance under extreme illumination and motion, we construct a laboratory scene under an extremely dark ambient condition (0.1 Lux on average), which contains local illumination ranging from 28,400 Lux (brightest) to 0.05 Lux (darkest), corresponding to a real scene illuminance dynamic range of 115 dB, and incorporate a rotating fan to illustrate dynamic distortion. The comparative results of different imaging methods under static and dynamic conditions are presented in Fig. 2.

In static conditions, synchronous temporal-mapping imaging successfully captures both the brightly lit text mennon on the color chart (red box) and the dimly lit digital reading 0.09 on the illuminance meter (blue box) in a single capture spanning 250 ms. This result demonstrates simultaneous preservation of high-fidelity details across the scene’s extreme brightness range. In contrast, E2VID^5^ fails to provide recognizable information across the entire image due to the absence of motion-induced events. Conventional frame-based imaging, limited by its inherent dynamic range, fails to capture both highlight and shadow regions in a single exposure. With an exposure time of 40 ms, the text mentioned in the red box is legible, but the digital reading in the blue box remains illegible even after post-processing enhancement. With a longer exposure time of 200 ms, the characters in the red box are overexposed and lost, while the digital reading in the blue box becomes visible. The fundamental advantage of temporal-mapping stems from its per-pixel adaptive exposure mechanism. Unlike conventional systems with uniform exposure time, each pixel in temporal-mapping adjusts its effective integration period based on local illumination. Bright regions trigger IPEs within microseconds, while dim regions may utilize tens to hundreds of milliseconds to integrate sufficient photons. At the 2 Hz modulation frequency, this adaptive exposure mechanism supports a theoretical dynamic range of 114 dB, as derived in Eq. (S1) of the supplemental document, enabling the faithful capture of reality across extreme highlights and shadows.

In the dynamic scene with a rotating fan, synchronous temporal-mapping imaging shows minimal motion blur within the yellow-boxed motion region. The edge of the white fan blade indicated by the arrow remains sharp, though some contrast degradation is observed in the black resolution line pairs in the background. By comparison, E2VID manages to reconstruct the rough shape of the fan in the dynamic region, but exhibits severe distortion in the recovered intensity of the blades. As for frame-based imaging, motion blur varies with exposure time. At the shorter exposure of 40 ms, the blade edge pointed by the arrow appears slightly blurred relative to the temporal-mapping result. At the longer 200 ms exposure, motion blur completely obscures the black line pairs in the background. The region becomes severely overexposed, rendering the blade structure indiscernible. The experimental observations can be explained as follows. The minimal motion blur in temporal-mapping imaging also originates from its adaptive exposure mechanism. At the bright fan blade, for instance, the IPE generation latency is only about 30 ms. The intensity information at that location is encoded at the very instant the IPE is generated, preventing further motion from introducing additional blur. In contrast, conventional frame-based imaging uses a fixed global exposure time, integrating motion throughout the entire exposure period, which leads to persistent motion blur. The intensity reconstruction distortion in E2VID’s output stems from its reliance on high-contrast features to trigger clear, unambiguous motion events. The fan blades, being a low-contrast target, fail to produce enough well-defined events, resulting in inaccurate intensity recovery.

While temporal-mapping demonstrates compelling snapshot imaging capability under slow synchronous modulation, extending it to video acquisition requires substantially higher modulation frequencies. However, as shown in the next section, increasing the frequency under synchronous modulation introduces severe artifacts-a fundamental issue that our asynchronous modulation approach effectively resolves.

#### Enabling video acquisition via asynchronous modulation

**Fig. 3:**
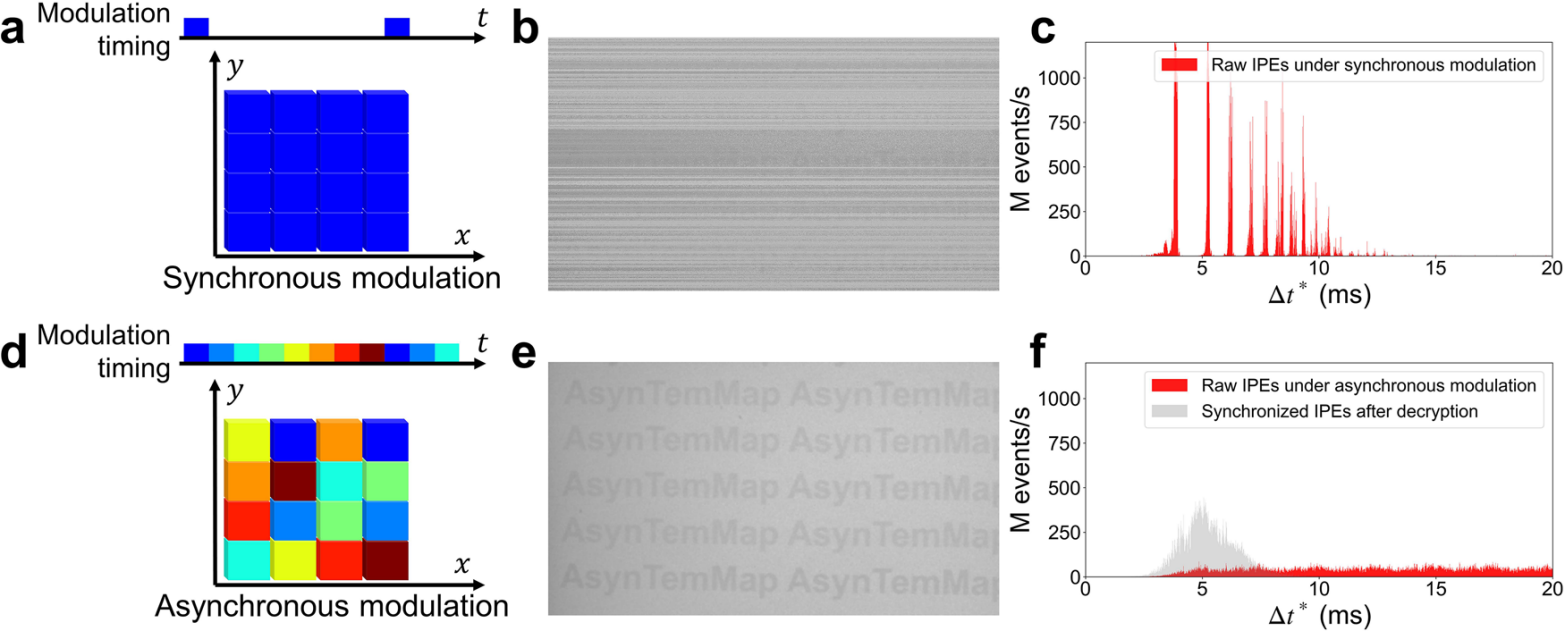
Comparison of synchronous and asynchronous modulation. Asynchronous modulation eliminates the row-wise striping artifacts observed in synchronous high-speed modulation by flattening the distribution of initial positive events (IPEs) and alleviating bandwidth pressure. **a** Schematic of micromirror flipping patterns under synchronous modulation. **b** Temporal-mapping image under synchronous modulation with severe stripe artifacts. **c** Under synchronous modulation, IPE timestamp distribution (red patch) shows event bursts and abnormal spikes. **d** Schematic of micromirror flipping patterns under asynchronous modulation. **e** Asynchronous modulation produces a clear, artifact-free image. **f** Under asynchronous modulation, the raw IPE distribution (red patch) is temporally uniform, and the synchronized IPEs after decryption (gray patch) match the expected response latency distribution.

Figure 3a, d schematically illustrate the core distinction between the two modulation schemes. Under synchronous modulation, all DMD micromirrors flip simultaneously, whereas asynchronous modulation drives each mirror to flip at a distinct, precisely controlled temporal offset, as indicated by the color-coded sequence. Both schemes operate all micromirrors at a uniform 50 Hz modulation, yielding 50 fps video of the identical low-contrast text sample.

The imaging outcomes are drastically different. The synchronous result (Fig. 3b) exhibits prominent horizontal bright stripes that severely degrade text readability. In contrast, the AsynTemMap result (Fig. 3e) presents clear text, completely free of stripe artifacts. The origin of these contrasting results is revealed by comparing the IPE timestamp distributions in Fig. 3c, f. Under synchronous modulation, the red patch in Fig. 3c shows an intense burst of IPEs around 4 ms after modulation onset, with the instantaneous rate exceeding 1000 M events/s. This surpasses the camera’s sustainable bandwidth, congesting the internal buffer and forcing a row-wise readout mode that assigns identical timestamps to all events within a row, thereby introducing random readout delays. The periodic nature of the row-wise readout mechanism continually disrupts temporal precision during IPE generation, creating the discrete spikes evident in the distribution. The consequent distortion of IPE generation latencies produces the horizontal stripes seen in Fig. 3b. Under asynchronous modulation, the raw IPE distribution, shown in the red patch of Fig. 3f, is uniform across the modulation cycle, reducing the peak event rate to 97 M events/s. This smooth generation alleviates bandwidth pressure and prevents row-readout artifacts. By computationally synchronizing the raw timestamps and subtracting their respective modulation onsets, the true generation latencies are recovered. The resulting synchronized distribution, shown in the gray patch of Fig. 3f, accurately reflects the scene’s physical reality, enabling the faithful intensity mapping in Fig. 3e.

This comparison clearly demonstrates the necessity of AsynTemMap over synchronous temporal mapping for video acquisition. Building upon this key advancement, we next demonstrate high-frame-rate video capture of real-world dynamic scenes and address additional challenges in the imaging pipeline.

### Neural reconstruction for faithful reality recovery

**Fig. 4:**
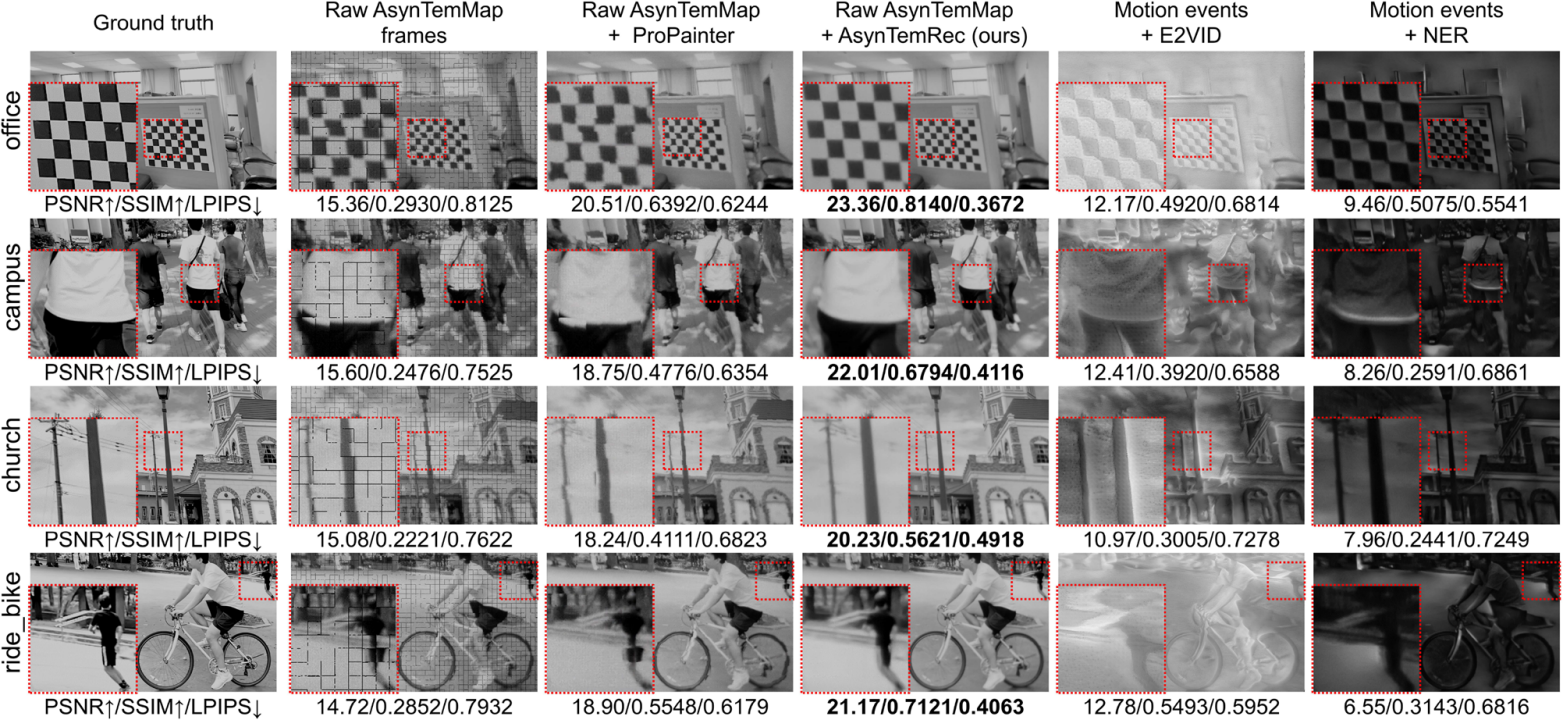
Qualitative and quantitative comparison of reconstruction performance under motion and hardware degradation. Columns (left to right): ground-truth images; raw Asynchronous Temporal Mapping (AsynTemMap) frames, exhibiting asynchronous sampling artifacts (ASA) and hardware defects; results after applying ProPainter^20^ to inpaint crack-like defects; results after applying the proposed reconstruction model, compensating for both ASA and hardware degradation; E2VID^5^ reconstructions from motion events; and NER^19^ reconstructions from motion events. Rows (top to bottom): office—a well-textured scene featuring fast linear motion in the foreground against a static background; campus—a complex dynamic scene involving non-linear human motion; church—a fast-motion scene featuring multi-scale structures; and ride_bike—a scene with humans in different movements near and far. The proposed reconstruction model achieves the highest PSNR/SSIM and lowest LPIPS in all cases.

With the introduction of pixel-wise asynchronous modulation, the AsynTemMap framework overcomes the bandwidth bottleneck, thereby extending the temporal-mapping principle to high-frame-rate video acquisition. However, this asynchronous sampling introduces a new challenge: rapid scene motion causes asynchronous sampling artifacts (ASA), where structures appear misaligned because different spatial locations are captured at distinct temporal instants, as evident in the raw AsynTemMap frames of Fig. 4. Furthermore, hardware imperfections-including DMD modulation defects, optical aberrations, and sensor noise-introduce additional degradations such as crack-like discontinuities and high-frequency noise. To recover faithful video from these corrupted observations, we develop a neural reconstruction model, AsynTemRec, which is designed to jointly compensate for both ASA and hardware-induced degradations.

Experimental setup. To validate AsynTemRec’s capability to overcome these challenges and recover faithful reality, we construct a real-world dataset of ten dynamic video sequences using the AsynTemMap prototype. The scenes are displayed on a 240 Hz monitor to ensure repeatable experiments and provide precise ground truth. The AsynTemMap system operates at a modulation frequency of 60 Hz. The captured raw IPEs are first decoded into raw AsynTemMap frames. These raw frames are then reconstructed using different neural models for performance comparison. In parallel, for event-based baseline comparison, we capture motion events for each sequence by fixing the DMD micromirrors in the on state, thereby disabling modulation, which turns the DMD into a static planar mirror, eliminating modulation-specific defects such as the crack-like patterns. We then reconstruct the videos using two representative motion-event-reconstruction methods: E2VID^5^ and the more recent NER^19^. This experimental setup ensures that both the AsynTemMap and the motion-event-reconstruction pipelines share identical optical conditions, including aberrations, thus enabling a direct and equitable comparison of their reconstruction performance. We compare four approaches in this section: (1) Raw AsynTemMap frames without neural processing; (2) Raw AsynTemMap + ProPainter^20^ for inpainting crack-like discontinuities; (3) Raw AsynTemMap + AsynTemRec (ours) for joint ASA and degradation correction; (4) Motion Events + E2VID^5^; and (5) Motion Events + NER^19^.

Implementation details. AsynTemRec is trained from scratch on a synthetic dataset generated using the same temporal offset key ${O}^{{\prime} }(x,y)$ as employed in the real test sequences. Details of the synthetic dataset generation, along with the network architecture, loss function, and training schedule, are provided in the Implementation Details of Reconstruction Model section of the supplemental document. To ensure a fair comparison, ProPainter is retrained on the same training dataset (including the identical temporal offset keys ${O}^{{\prime} }(x,y)$) used for AsynTemRec, following its publicly available training schedule. In contrast, both E2VID and NER are used in their default, pre-trained configurations without further fine-tuning, as their input (standard motion events) matches the data modality they were originally designed for.

Inference speed. For practical deployment, we measure the inference speed of AsynTemRec on an NVIDIA RTX 4090 GPU with FP32 precision. AsynTemRec processes an input frame of size 1280 × 720 in 198.82 ms on average, corresponding to approximately 5 frames per second. This speed is acceptable for offline, high-quality reconstruction applications where reconstruction fidelity is prioritized over real-time performance.

Figure 4 presents a qualitative comparison of three representative scenes, while Table 1 summarizes the quantitative results across all ten sequences. The original video sequences displayed on the monitor serve as the ground truth. All outputs are spatially and temporally aligned to this ground truth for quantitative evaluation.

**Table 1:** Quantitative comparison across methods on all test sequences

Sequence	Raw AsynTemMap			Raw AsynTemMap + ProPainter^20^			Raw AsynTemMap + AsynTemRec (ours)			Motion events + E2VID^5^			Motion events + NER^19^		
	PSNR*↑*	SSIM*↑*	LPIPS*↓*	PSNR*↑*	SSIM*↑*	LPIPS*↓*	PSNR*↑*	SSIM*↑*	LPIPS*↓*	PSNR*↑*	SSIM*↑*	LPIPS*↓*	PSNR*↑*	SSIM*↑*	LPIPS*↓*
drone_lake	21.07	0.4713	0.5092	23.06	0.5789	0.4500	23.96	0.6607	0.3833	5.89	0.1040	0.8427	14.95	0.2372	0.6049
alley_1	15.21	0.2313	0.7377	18.66	0.4485	0.6666	20.58	0.6114	0.4574	12.11	0.3715	0.6840	8.56	0.3052	0.6622
alley_2	14.86	0.2309	0.7327	18.63	0.4525	0.6624	20.37	0.6125	0.4442	11.26	0.3683	0.7069	8.04	0.3083	0.6446
catch_ball	16.52	0.2776	0.7356	22.15	0.4991	0.5251	22.33	0.5625	0.4174	10.08	0.3345	0.5822	9.13	0.2957	0.5810
ride_bike	15.01	0.2940	0.7827	19.70	0.5433	0.6064	21.15	0.6920	0.4044	12.40	0.5201	0.5742	6.53	0.3103	0.6828
campus	15.47	0.2471	0.7361	18.87	0.4555	0.6342	21.48	0.6443	0.4264	12.28	0.3539	0.6465	8.32	0.2578	0.6908
shrub	14.74	0.1791	0.7147	17.69	0.3482	0.6369	19.49	0.4855	0.4983	11.26	0.2136	0.6421	7.98	0.1603	0.7279
church	14.97	0.2199	0.7478	17.76	0.3824	0.6682	19.96	0.5506	0.4932	11.79	0.3109	0.7207	7.82	0.2214	0.7317
office	15.31	0.2907	0.8038	20.29	0.5991	0.6321	23.48	0.8103	0.3749	11.98	0.4795	0.7010	9.58	0.5003	0.5749
traffic	15.69	0.2216	0.7545	19.01	0.4230	0.6194	21.44	0.5924	0.4136	11.80	0.2729	0.6734	9.36	0.2388	0.6463
Average	15.89	0.2663	0.7255	19.58	0.4731	0.6101	21.42	0.6222	0.4313	11.09	0.3329	0.6774	9.03	0.2835	0.6547

Higher values indicate better performance for PSNR and SSIM, while lower values indicate better performance for LPIPS.

The qualitative results of four representative scenes in Fig. 4 reveal the distinct reconstruction performance of each method under various dynamic conditions. Raw AsynTemMap frames universally exhibit geometric misalignment in moving regions due to asynchronous sampling, alongside hardware-induced crack-like discontinuities and noise. ProPainter effectively addresses crack-like discontinuities caused by DMD defects but fails to correct motion-induced ASA. In contrast, AsynTemRec robustly addresses both hardware-induced degradations and ASA, effectively restoring original structures in dynamic regions. E2VID, which operates on motion events, not only fails to reconstruct information in slow-moving or static regions but also produces distorted recoveries even in areas with apparent motion. The distinct challenges of each scene further highlight these characteristics. In the office scene, which mixes rapid motion with static regions, AsynTemRec successfully corrects both the misaligned checkerboard and crack-like defects. ProPainter effectively restores static areas like the distant windows, but leaves the moving checkerboard severely distorted. E2VID shows the opposite tendency-it roughly recovers the moving checkerboard but fails to perceive the static background. The campus scene, featuring non-linear motion, demonstrates AsynTemRec’s capability to handle non-rigid deformation. It accurately restores the misaligned garment corner within the red box, while preserving subtle wrinkles. ProPainter merely fills the pixel-level discontinuities without correcting the structural misalignment. E2VID completely confuses the intensity values, failing to distinguish between the white shirt and dark trousers. The church scene, containing both coarse and fine structures in motion, presents challenges for scene reconstruction. In the raw AsynTemMap frames, the finest horizontal wires exhibit inherently low contrast due to optical aberrations in the system. Both ProPainter and AsynTemRec manage to recover the basic structure of these wires, with AsynTemRec providing a cleaner result by effectively suppressing noise. However, neither method effectively enhances the contrast of these subtle textures. We attribute this limitation primarily to the optical system’s aberrations, which attenuate the signal of fine, low-contrast structures in the raw AsynTemMap frames before reconstruction. This attribution is directly supported by a simulation experiment: when AsynTemRec is tested on a synthetic church sequence free from optical aberrations (see Fig. S5 in the supplemental document), fine features like the wires are recovered with high fidelity. Therefore, the most effective strategy to enhance the recovery of such fine details is to improve the optical system. A secondary factor is the training objective: the pixel-wise MSE loss used in AsynTemRec prioritizes overall fidelity and naturally yields smooth outputs, but does not specifically amplify weak signals. For tasks requiring enhanced detail, supplementing the perceptual or adversarial loss could be beneficial, though it may introduce non-physical artifacts. In stark contrast, E2VID fails entirely to recover the fine wires, as their inherently low contrast, combined with motion nearly parallel to their orientation, results in negligible luminance change and a critical lack of motion events. The ride_bike scene features a camera tracking a nearby cyclist moving at a moderate speed, while a distant runner in the upper-right corner exhibits much faster relative motion. In the raw AsynTemMap frames, this runner region in the red box shows pronounced ASA. Comparing the reconstructions for raw AsynTemMap frames, AsynTemRec effectively corrects this misalignment, whereas ProPainter merely inpaints the crack-like defects without resolving the structural distortion. E2VID, reconstructing solely from motion events, only recovers the foreground cyclist; the contour of the distant runner is severely distorted. For a more comprehensive evaluation, we also include the recent NER^19^ as an additional motion-event-reconstruction baseline, which is specifically designed for challenging low-light conditions similar to our experimental setup. Qualitatively, NER shows a nuanced trade-off compared to E2VID: it produces slightly sharper contours and better-defined textures in some structured regions (e.g., the checkerboard in the office and the wire poles in the church). However, this comes at the cost of more severe photometric distortion; NER’s reconstructions are consistently darker and exhibit greater intensity inaccuracy across all scenes. As visually evident in the examples of Fig. 4, this trade-off results in NER’s overall reconstruction metrics being comparable to, or even lower than, that of E2VID. Critically, both E2VID and NER exhibit a common trait of motion-event-reconstruction methods: they cannot achieve true photometric fidelity.

The quantitative results in Table 1 corroborate the qualitative observations: AsynTemRec performs best across all three metrics. Its superior PSNR and SSIM reflect high pixel-level and structural fidelity to the ground truth, while its lowest LPIPS confirms superior perceptual realism. This advantage originates from fundamental methodological differences. Within the AsynTemMap paradigm, AsynTemRec’s comprehensive physical modeling of both ASA and hardware degradations enables globally coherent reconstructions, outperforming ProPainter, which excels at local inpainting but lacks a global model to correct motion distortions. The overall AsynTemMap paradigm, integrated with neural reconstruction, demonstrates clear superiority over the conventional motion-events-reconstruction approach. The key advantage lies in its proactive encoding of the imaging process and subsequent algorithmic reconstruction under a unified physical model, enabling it to faithfully recover reality. In contrast, conventional methods rely on inferred scene content from passive brightness changes. This underscores the critical role of a computational optical imaging framework in empowering event-based vision to truly capture reality.

### Confidential vision through hardware-level encryption

**Fig. 5:**
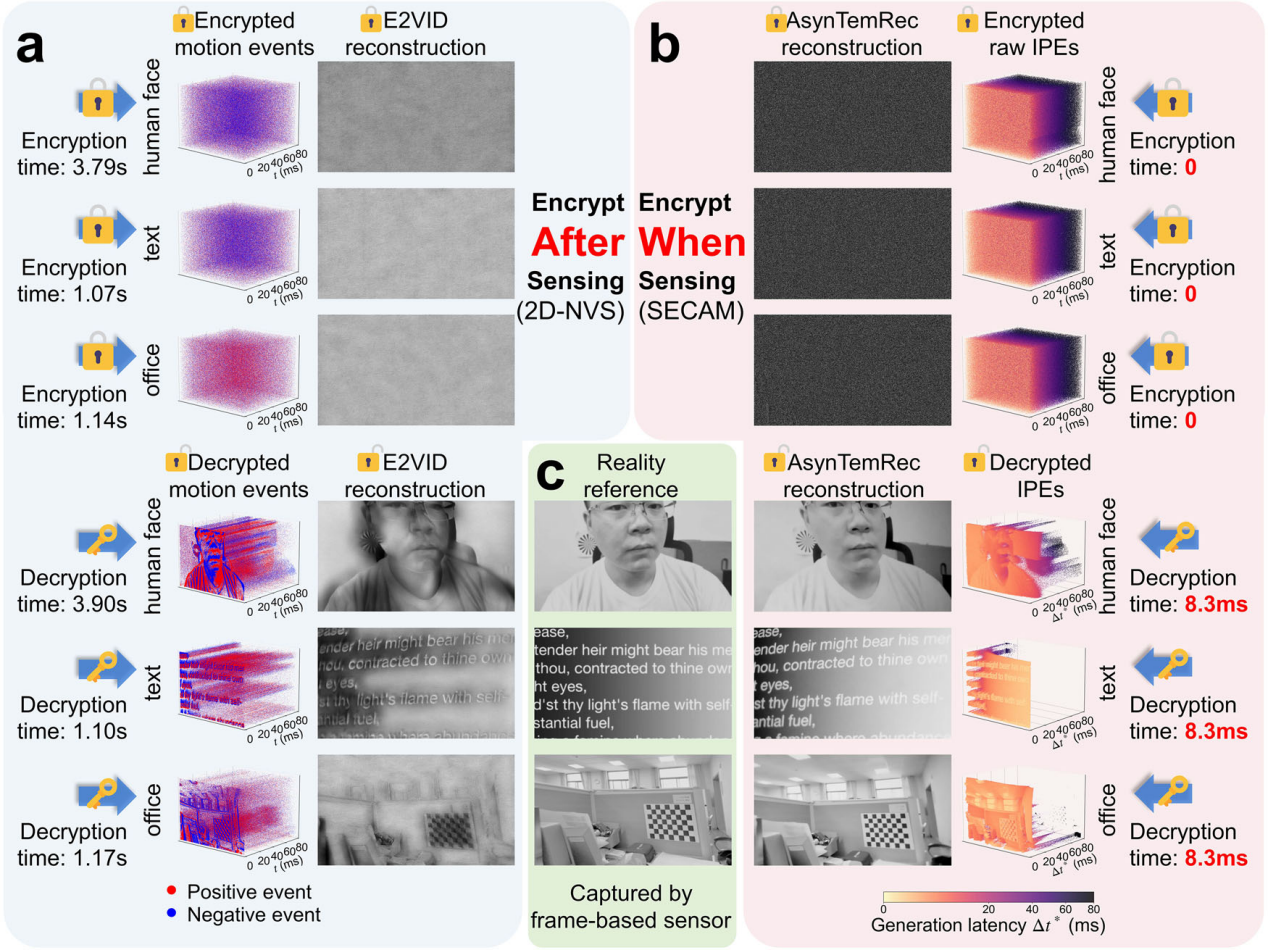
Comparison of encryption paradigms for event-based vision. **a** Post-sensing software encryption (2D-NVS)^21^. The raw motion events are encrypted after acquisition, and the ciphertext/plaintext is reconstructed with E2VID^5^. While visual obfuscation is achieved, this method incurs a high encryption and decryption latency ( > 1 s for 80 ms of events). **b** In-sensing hardware encryption (Secure Event Coding via Asynchronous Modulation, SECAM). The scene is optically encrypted into chaotic raw initial positive events (IPEs) with zero latency during acquisition. Decrypting a frame takes only 8.3 ms; subsequent reconstruction faithfully restores the scene. **c** Scene reference captured by a conventional frame-based sensor for visual comparison; the images are not spatially aligned with reconstruction results. Three representative privacy-sensitive scenes are shown: human face, text, and office. Both encryption paradigms provide visual obfuscation, but SECAM simultaneously offers near-zero-latency hardware encryption and higher-fidelity reconstruction.

Beyond capturing reality, modern imaging systems are also expected to provide inherent privacy protection to achieve confidential vision. While event-based vision encodes brightness changes into asynchronous event streams instead of recording absolute intensities, conventional motion events still expose scene contours when accumulated into event frames, as shown at the bottom of Fig. 5a. E2VID^5^ reconstructions further reveal fine-grained details, rendering individual facial features discernible, text content legible, and the spatial layout of offices clearly visible. Thus, traditional event-based systems cannot ensure complete inherent privacy, leaving visual encryption dependent on post-processing software methods^21^. The top of Fig. 5a shows the ciphertext event point cloud after post-processing encryption of raw motion events using the existing 2D-NVS algorithm^21^, along with the results after reconstruction using E2VID. Although this encryption method successfully protects privacy, it inevitably introduces encryption time overhead, which limits its real-time application in high-speed vision systems. The encryption/decryption process incurs a substantial time overhead, with events generated within 80 milliseconds requiring more than 1 second to be processed on a single-core 3.2 GHz CPU.

In contrast, AsynTemMap encodes scene intensity into a chaotic spatiotemporal event stream via asynchronous modulation, as evident in the raw encrypted IPEs shown at the top of Fig. 5b. This process inherently conceals structural information, a property termed Secure Event Coding via Asynchronous Modulation (SECAM). Unlike post-processing encryption, SECAM operates at the acquisition stage, providing zero-latency, hardware-level encryption. Unauthorized decoding without the correct key produces noise-like ciphertext bearing minimal resemblance to the plaintext (SSIM  ≈ 0.01 across three scenes), effectively obscuring all critical scene features. The recovery of IPE generation latencies from the raw timestamps constitutes the SECAM decryption process. Using the correct temporal offsets key ${O}^{{\prime} }(x,y)$ to align the IPE timestamps transforms them into their true latency distributions, visualized as the Decrypted IPEs in the bottom of Fig. 5b. Applying Eq. (1) and AsynTemRec to these decrypted events yields images that faithfully restore the scene reality depicted on the right bottom, with authorized decrypted IPEs through AsynTemRec providing a closer match to the true scene reference compared to the reconstruction of decrypted motion events. As for the time overhead, SECAM takes a fixed 8.3 ms to decrypt the raw IPEs corresponding to a single frame on a single-core 3.2 GHz CPU, which is markedly faster than the several seconds of decryption overhead required by 2D-NVS on motion events.

In Fig. 5, we have qualitatively compared 2D-NVS on motion events and SECAM on AsynTemMap IPEs. To ensure a fair and quantitative comparison of the two event encryption methods, we include experiments on 2D-NVS applied to plaintext IPEs (correctly decrypted IPEs), alongside SECAM on AsynTemMap IPEs. This allows us to demonstrate the advantages of SECAM’s event encryption paradigm over 2D-NVS in terms of plaintext-ciphertext similarity, key sensitivity, ciphertext randomness, and encryption/decryption latency. For fair comparison, plaintext and ciphertext data are converted to grayscale images via Eq. (1) before computing standard image encryption metrics^22^. Detailed results are summarized in Table 2. Both methods achieve extremely low plaintext-ciphertext similarity, effectively concealing visual information. However, SECAM demonstrates stronger security properties: it exhibits near-perfect key sensitivity (NPCR  ≈ 99%) compared to 2D-NVS (74%), and higher ciphertext entropy (7.40 bits vs. 4.54 bits), indicating improved resistance to statistical attacks. The most notable advantage lies in efficiency. As a hardware-level paradigm, SECAM achieves zero encryption latency with a decryption time of only 0.009 μs/event. This enables a real-time processing rate of 111 M events/s-a throughput sufficient for real-time encryption and decryption on the fly during the capture of a 1280  × 720 @ 120 FPS video stream via the AsynTemMap system. In stark contrast, 2D-NVS, as a symmetric software-based algorithm, incurs comparable costs for both encryption and decryption (approximately 1.9 *μ* s/event), making it nearly 200 times slower than SECAM and entirely inadequate for high-speed vision applications.

**Table 2:** Quantitative comparison of different event encryption methods

Metric type	Metric	SECAM	2D-NVS^21^
Plain–Ciphertext Similarity	PSNR (dB) *↓*	6.15	6.03
	SSIM *↓*	0.01	0.01
Key Sensitivity	NPCR (%) *↑*	98.98	74.29
Ciphertext Randomness	Entropy (bit) *↑*	7.40	4.54
Encryption Time	*μ* s/event *↓*	0	1.85
Decryption Time	*μ* s/event *↓*	0.009	1.90

The Normalized Pixel Change Rate (NPCR) quantifies the percentage of differing pixels in the ciphertext when a single bit of the secret key is altered.

SECAM establishes a hardware-level encryption paradigm natively designed for event-based vision. While optical encryption concepts exist for frame-based systems (e.g., single-pixel imaging), SECAM is not a mere adaptation of such methods. Instead, it intrinsically exploits the asynchronous sensing principle of event cameras: encryption is achieved by randomizing the per-pixel modulation onset within the same optical encoding process designed for high-fidelity video acquisition. This co-design means that privacy protection incurs no additional system complexity-the encryption is accomplished simultaneously with faithful video capture, resulting in the zero-latency advantage demonstrated in Table 2. Furthermore, as a hardware-level paradigm, SECAM is hardware-friendly and readily deployable. In the Encryption Characteristics under Hardware Constraints section of the supplemental document, we explore in greater detail how hardware constraints influence encryption characteristics. We conclude that for capturing AsynTemMap video at 50 FPS with 1280 × 720 resolution, a spatial light modulation resolution of at least 128  × 72 and a modulation frequency of no less than 500 Hz are sufficient to achieve optimal encryption performance, which is easily achievable with current commercial DMD products. In summary, the SECAM embedded in AsynTemMap offers an efficient, secure, and easily deployable hardware-level visual encryption solution.

### Threat model and attack analysis

#### Threat model definition

In this section, we define the threat model considered for the security analysis of SECAM and outline three types of adversaries with progressively increasing capabilities: a ciphertext-only attacker, a known-plaintext attacker (KPA), and a chosen-plaintext attacker (CPA). The ciphertext-only attacker is the weakest adversary, having access only to the encrypted event stream and no knowledge of the original scene or the encryption key. Under this setting, the attacker can only exploit statistical or side-channel information from the ciphertext itself. We evaluate SECAM under this threat model through side-channel analyses. The known-plaintext attacker is assumed to have access to both the ciphertext and partial plaintext information. This setting reflects more practical attack scenarios where limited scene content may be known or inferred. We assess SECAM under this threat model via quantitative leakage analysis and demonstrate its advantage over 2D-NVS. The chosen-plaintext attacker represents the strongest adversary, with the ability to actively select scene inputs and observe the corresponding ciphertext. Rather than claiming formal security against this attack, we discuss the potential risks of SECAM under a naive hardware configuration and outline feasible engineering mitigation strategies. Together, these three threat models provide a structured framework for analyzing the security properties of SECAM, from passive observation to increasingly powerful adversarial capabilities.

#### Ciphertext-only attacker: side-channel analysis

For a ciphertext-only attacker, besides brute-force decryption, the only available attack vector is to exploit the statistical information inherent in the ciphertext. We refer to this as side-channel analysis.

**Fig. 6:**
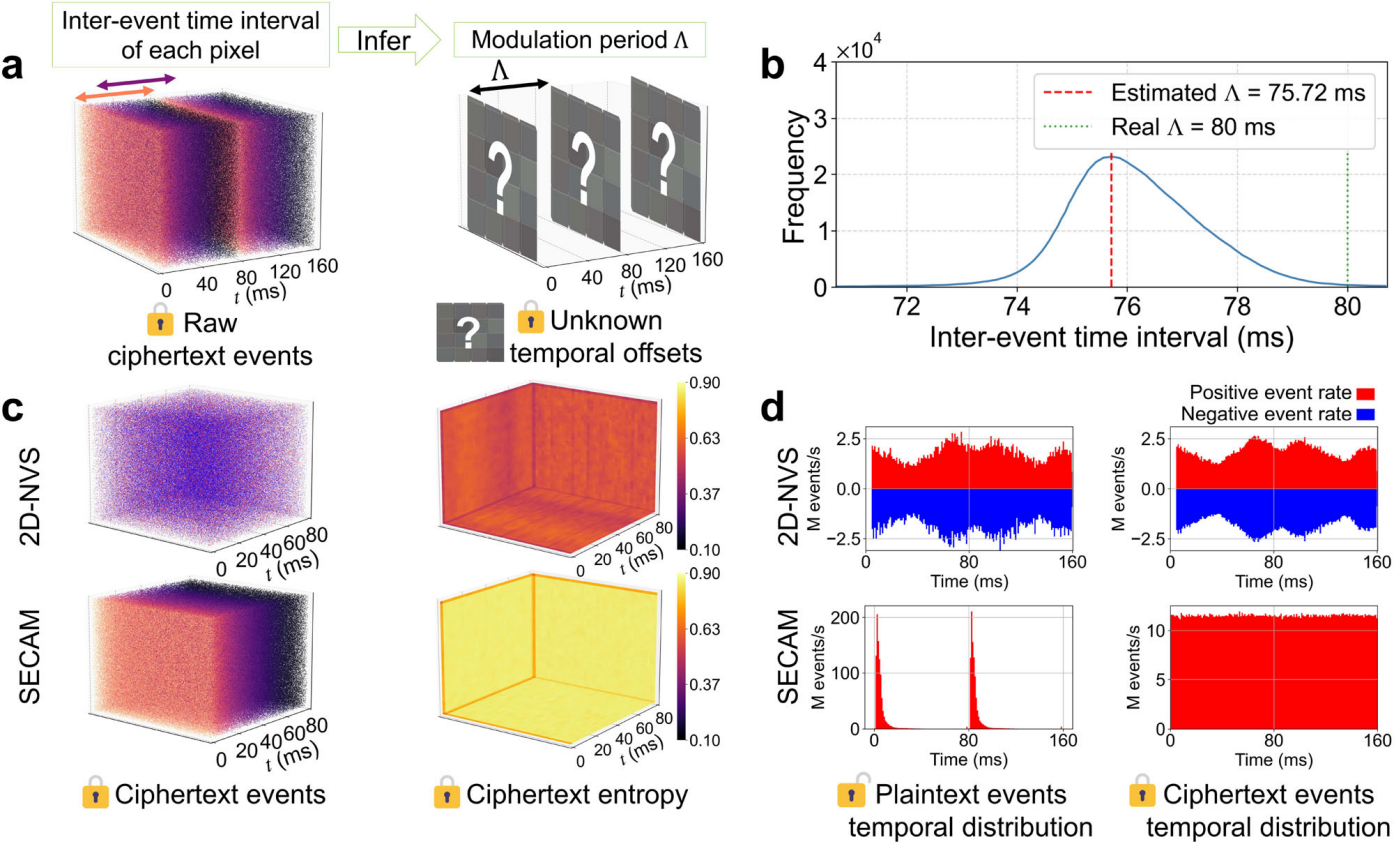
Side-channel analysis experiment conducted by the ciphertext-only attacker. **a** The ciphertext events of Secure Event Coding via Asynchronous Modulation (SECAM) have certain time repeatability under the periodic modulation of Digital Micromirror Device (DMD). The DMD modulation period *Λ* can be inferred from the time interval when each pixel generates events, which is a fixed system property independent of the visual scene content. **b** Event time interval analysis of the ciphertext captured by SECAM to estimate the DMD modulation period *Λ*. Due to the influence of trailing events, the *Λ* estimated from this analysis is smaller than the true value. **c** Spatiotemporal entropy analysis of the ciphertext for both 2D-NVS^21^ and SECAM. The results show that SECAM's entropy is consistently higher than that of 2D-NVS, exhibiting stronger statistical randomness. Additionally, 2D-NVS's entropy markedly decreases between 40 and 60 ms. **d** Further analysis of the temporal distribution characteristics of the ciphertext for both 2D-NVS and SECAM. 2D-NVS almost fully exposes the temporal distribution information of the plaintext, allowing the attacker to infer motion and scene texture changes. In contrast, SECAM's ciphertext is evenly distributed in time, demonstrating resilience to statistical analysis.

For the SECAM paradigm, due to the cyclic modulation of the DMD, the ciphertext inherently contains periodic repetition characteristics, especially when capturing a static or slowly varying scene. This periodicity presents an initial opportunity for the ciphertext-only attacker to perform side-channel analysis. Theoretically, under the fixed modulation period *Λ* of the DMD, the time intervals between positive events generated at the same position should correspond to *Λ*, as schematically illustrated in Fig. 6a. To investigate this, we extract approximately 160 ms of raw IPEs from the face sequence and compute the time intervals between consecutive positive events for each pixel. The distribution of these inter-event time intervals is shown in Fig. 6b. A clear peak is observed in the histogram, where the peak position corresponds to an estimated *Λ* = 75715 *μs*, which is smaller than the actual DMD modulation period of 80ms. This discrepancy arises because, after each modulation onset by the DMD, in addition to generating IPEs, a small number of trailing events are randomly generated during the subsequent period. Therefore, the estimated *Λ*, based on the inter-event time interval peak, is smaller than the true *Λ*. This shows that the hardware-induced noise from trailing events provides some level of resistance to side-channel attacks on SECAM.

In the subsequent experiment, we assume that the ciphertext-only attacker has successfully inferred the correct *Λ* by other means and has obtained the corresponding ciphertext for a frame. The attacker proceeds to analyze the statistical distribution characteristics of the ciphertext in order to extract features of the plaintext. In Fig. 6c, we compare the spatiotemporal characteristics of raw IPEs captured by SECAM with those of motion events encrypted using 2D-NVS. This comparison evaluates the statistical properties of the ciphertexts produced by the two encryption paradigms operating on their respective native data types. To quantify the randomness of the ciphertext event distribution, we employ the normalized spatiotemporal entropy:

2 $$H(i,j,k)=\left\{\begin{array}{ll}\frac{-{\sum }_{\Omega }\frac{{n}_{\Omega }}{N}\log \frac{{n}_{\Omega }}{N}}{\log M}, & N > 0\\ 0, \hfill & N=0\end{array}\right.$$

where *H*(*i*, *j*, *k*) denotes the normalized local spatiotemporal entropy centered at voxel (*i*, *j*, *k*). Events are first discretized into a spatiotemporal grid with voxel dimensions of 8 pixels (spatial) and 1.0 ms (temporal). The summation in the entropy calculation is performed over all voxels *Ω* within a *K*_*X*_ × *K*_*Y*_ × *K*_*T*_ neighborhood centered at (*i*, *j*, *k*), where we set *K*_*X*_ = *K*_*Y*_ = *K*_*T*_ = 5. This corresponds to a local spatiotemporal volume containing *M* = *K*_*X*_*K*_*Y*_*K*_*T*_ = 125 voxels. Here, *n*_*Ω*_ is the number of events in voxel *Ω*, and *N* = ∑_*Ω*_*n*_*Ω*_ is the total number of events in that neighborhood. A value of *H*(*i*, *j*, *k*) closer to 1 indicates a more uniform spatiotemporal distribution of events within the local volume, implying weaker statistical structure and thus enhanced resistance to side-channel analysis of the ciphertext. As shown in Fig. 6c, the entropy of the SECAM ciphertext is consistently higher than that of 2D-NVS, demonstrating that SECAM produces a ciphertext with reduced statistical regularities and thus enhanced resistance to side-channel attacks.

A more detailed comparison of the ciphertext entropy in Fig. 6c reveals that the SECAM ciphertext exhibits uniform entropy projections along the X, Y, and T axes, while the entropy of the 2D-NVS ciphertext shows a noticeable drop between 40 and 60ms. This suggests that there might be a vulnerability in the temporal distribution characteristics of 2D-NVS ciphertext. To explain this phenomenon, we further compare the event rate over time for both plaintext and ciphertext of 2D-NVS and SECAM in Fig. 6d. It can be seen that the event distribution characteristics of 2D-NVS plaintext and ciphertext are similar, and the event rate decreases between 40 and 60 ms, indicating a reduction in scene motion or texture. The temporal vulnerability of 2D-NVS allows the ciphertext-only attacker to analyze motion or scene changes from the event stream. In contrast, SECAM’s ciphertext events are uniformly distributed in time, effectively masking the temporal distribution features of the plaintext, making it difficult for a ciphertext-only attacker to conduct statistical analysis for side-channel attacks.

These analyses demonstrate that a ciphertext-only attack on SECAM faces two formidable barriers: first, accurately estimating the modulation period *Λ* from the noisy ciphertext is difficult in practice; second, and more critically, the statistical uniformity of the ciphertext, evidenced by its consistently high spatiotemporal entropy and temporally flat distribution, provides no leverage for reconstructing the visual plaintext even if *Λ* were known. Thus, the core security mechanism of SECAM successfully decouples the ciphertext from the plaintext in both space and time. Consequently, this fundamental spatiotemporal decorrelation ensures that statistical analysis of the ciphertext alone yields negligible information about the original scene, robustly fulfilling the requirement for confidentiality against ciphertext-only adversaries.

#### Known-plaintext attacker (KPA) analysis on a periodic pattern

**Fig. 7:**
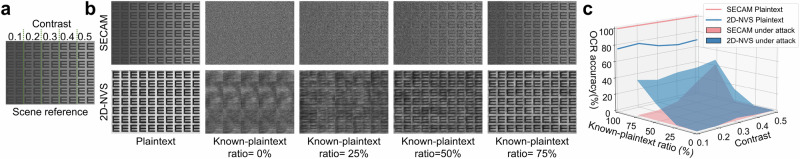
Comparison of resistance to known-plaintext attacks between Secure Event Coding via Asynchronous Modulation (SECAM) and 2D-NVS^21^ event encryption. **a** Periodic pattern of the character E as a controlled test pattern. **b** Reconstructed ciphertext images of character E under varying scene contrasts (0.1–0.5) and known-plaintext ratios: SECAM leaks high-contrast features only at high known-plaintext ratios, while 2D-NVS exhibits consistent leakage regardless of contrast. **c** Optical character recognition (OCR) accuracy as a quantitative metric of information leakage: SECAM achieves substantially lower ciphertext OCR accuracy than 2D-NVS across all attack conditions, demonstrating stronger confidentiality. The 100% plaintext OCR accuracy for SECAM confirms faithful reconstruction of the original scene—establishing a valid baseline for security evaluation—whereas 2D-NVS's degraded plaintext accuracy ( < 80%) stems from reconstruction distortion, not enhanced privacy.

In event-based encryption, a known-plaintext attack (KPA) assumes the adversary possesses partial knowledge of the plaintext event stream and attempts to infer visual content from the reconstructed grayscale image. To realistically evaluate information leakage under such threats, we design an experiment that varies both the known-plaintext ratio (simulating the attacker’s prior knowledge) and the scene contrast (reflecting real-world imaging conditions). We use a spatially periodic pattern of the character E as a controlled test pattern, as shown in Fig. 7a, and employ the open-source Tesseract-OCR software^23^ to quantitatively measure optical character recognition (OCR) accuracy on both plaintext reconstructions and ciphertext reconstructions. This provides a principled, task-driven benchmark for visual information leakage across encryption schemes. We compare two event encryption approaches: SECAM IPEs reconstructed with AsynTemRec, and 2D-NVS^21^ encrypted motion events reconstructed with E2VID^5^.

Figure 7b displays the plaintext and corresponding ciphertext images reconstructed under varying known-plaintext ratios, with SECAM results shown in the top row and 2D-NVS in the bottom row. For each contrast level, we capture one dataset (SECAM raw IPEs and, separately, raw motion events for 2D-NVS input). The images shown are excerpts from reconstructions performed independently at different known-plaintext ratios and then stitched together for visualization. Figure 7c quantifies information leakage via OCR accuracy across scene contrast levels (0.1-0.5) and known-plaintext ratios: solid curves represent plaintext OCR accuracy (100% known events), while colored surfaces depict ciphertext OCR accuracy under partial knowledge.

Plaintext comparison. When the full plaintext is known (leftmost column of Fig. 7b), SECAM’s reconstruction faithfully reproduces the ground-truth character E across all contrast levels. This high-fidelity reconstruction is confirmed by the 100% OCR accuracy for all contrasts (red curve in Fig. 7c), establishing a valid baseline for security evaluation. In contrast, the plaintext reconstructions for 2D-NVS (i.e., motion events reconstructed with E2VID) appear nearly identical across contrast levels and exhibit noticeable geometric distortion, resulting in a plaintext OCR accuracy below 80% (blue curve).

Ciphertext analysis. When only the ciphertext is available (known-plaintext ratio = 0%), SECAM’s output appears chaotic and shows no discernible periodic structure corresponding to the repeated E pattern, regardless of contrast. This confirms that the spatially random key ${O}^{{\prime} }(x,y)$ effectively provides diffusion in the spatial domain, thereby preventing ECB-penguin-like leakage. In contrast, 2D-NVS ciphertext exhibits a concerning consistency across contrast levels: its reconstructed images appear very similar for different contrast scenes. This occurs because the raw motion events generated by the character E at various contrast levels are inherently similar. More importantly, the 2D-NVS algorithm processes these similar plaintext streams through a deterministic cryptographic process. Consequently, similar plaintexts lead to statistically similar ciphertexts, resulting in nearly identical reconstructions. This allows statistical patterns from the plaintext to persist in the ciphertext, a behavior reminiscent of the well-known weakness in the ECB encryption mode^24^.

Partial known-plaintext analysis. As the known-plaintext ratio increases, SECAM exhibits contrast-dependent leakage: higher-contrast instances become visually discernible first. The lowest-contrast character (0.1) remains largely unrecognizable until the known-plaintext ratio reaches 75%. Quantitatively, at 75% known-plaintext ratio, the OCR accuracy for the highest-contrast character (0.5) reaches only 40% and drops sharply with decreasing contrast or known-plaintext ratio (red surface in Fig. 7c). In contrast, 2D-NVS shows consistent information leakage across all contrast levels, with ciphertext OCR accuracy consistently higher than SECAM’s under the same attack conditions (blue surface). This indicates greater vulnerability to known-plaintext attacks.

These results demonstrate that SECAM provides effective resistance to known-plaintext attacks while maintaining high-fidelity reconstruction capability, whereas 2D-NVS exhibits higher information leakage under the same attack conditions. This divergence stems from the fundamentally different types of event data they encrypt. SECAM operates on intensity-encoded events that retain absolute photometric information. Its encryption scrambles pixel-level intensity values, which inherently imposes stronger perturbation on low-contrast, weak-signal features. This property directly enhances privacy: the pixel-wise intensity scrambling imposed by SECAM’s encryption disproportionately affects low-contrast features (e.g., watermarks, fine facial textures). As a result, even with partial known-plaintext, such sensitive content remains effectively shielded from leakage. In contrast, 2D-NVS encrypts motion-triggered events that reflect scene edges and texture. Due to the high redundancy of these events in representing structural content, even a partial set of known events often enables an adversary to infer the dominant contours of the scene. Consequently, this encryption paradigm offers inherently weaker privacy protection: low-contrast features either fail to generate sufficient events to faithfully represent the underlying scene, or, once encoded, are just as vulnerable to reconstruction from partial known-plaintext as high-contrast ones.

#### Discussion on chosen-plaintext attacker (CPA)

A chosen-plaintext attacker (CPA) possesses the capability to actively choose input patterns to the imaging system and observe the corresponding ciphertexts, constituting a powerful attack model that typically assumes a high degree of access to or control over the target. For SECAM, mounting a practical CPA would generally require direct physical access to the hardware to precisely inject controlled optical patterns, a scenario that extends beyond our defined threat model of a remote or passive eavesdropper. Nevertheless, we discuss the potential risks and mitigation strategies for completeness.

The most effective CPA against SECAM would involve injecting a spatially uniform, high-intensity light pulse at a known time *t*_inj_. Under such a condition, the IPE generation latency Δt* for every pixel approaches its minimum, causing the recorded event timestamps to cluster near ${t}_{inj}+{O}^{{\prime} }(x,y)$. An adversary could then analyze the temporal offsets in the ciphertext event stream to statistically infer the spatial key map ${O}^{{\prime} }(x,y)$.

The primary risk arises if the same static key ${O}^{{\prime} }(x,y)$ is used indefinitely across a long capture session. To mitigate this, we emphasize that SECAM’s security in operational settings does not rely on long-term key secrecy alone. As demonstrated by the high key sensitivity (NPCR ≈ 99%) in Table 2, even a minor change in the key completely alters the ciphertext. This property enables a straightforward and effective countermeasure: frequent key renewal. By randomly regenerating and reloading the DMD offset map ${O}^{{\prime} }(x,y)$, any partially recovered key information becomes obsolete. The computational and operational overhead for this renewal is negligible, as it involves only reloading a precomputed pattern to the DMD.

Therefore, while a classical CPA with extensive hardware access poses a theoretical challenge, the proposed system can be robustly secured against such advanced threats in practice through simple key management. This maintains the confidentiality of video streams without compromising the efficiency and hardware-native advantages of the SECAM paradigm.

## Discussion

While the proposed AsynTemMap system demonstrates compelling capabilities in high-dynamic-range and privacy-secure visual acquisition, certain limitations present opportunities for future development. In the current system, the encryption and decoding processes operate in real time at the sensor level, whereas the neural reconstruction is performed offline to achieve high-quality results. The current prototype captures grayscale information due to the monochromatic nature of commercially available high-resolution event sensors. Color imaging could be achieved through conventional Bayer filter integration or, more naturally within our framework, via time-division multiplexed color acquisition, dividing each modulation period into RGB sub-intervals to encode color components temporally while preserving the asynchronous encoding principle.

Beyond capturing reality and preserving privacy, AsynTemMap offers a pathway toward active visual perception. Its pixel-wise modulation allows for selective spatiotemporal sampling, with each IPE providing a direct measurement of instantaneous intensity at a specific coordinate. This capability lays the foundation for adaptive acquisition strategies that dynamically focus on salient regions, advancing passive imaging toward an intelligent, task-driven active sensing framework.

In summary, we have introduced AsynTemMap, an event-based computational optical imaging framework that integrates (1) optical encoding, (2) computational decoding, and (3) neural reconstruction. In the current implementation, real-time encryption and decoding are complemented by offline high-quality reconstruction, enabling efficient data acquisition and high-fidelity recovery. By integrating physically grounded temporal mapping with data-driven learning, our system achieves faithful reality capture under extreme illumination and motion while providing intrinsic hardware-level encryption through SECAM. The highly flexible visual sampling capability of AsynTemMap also establishes a foundation for future low-redundancy, intelligent, and active visual perception systems.

## Methods

In this section, we outline the key components of the AsynTemMap system. The complete imaging pipeline consists of three stages: (1) encoding scenes into asynchronous IPEs via pixel-wise modulation; (2) decoding IPEs into raw frames; and (3) reconstructing high-fidelity videos using AsynTemRec.

### Optical encoding

Asynchronous encoding is implemented using a predefined temporal offset map *O*(*i*, *j*), where each element specifies the modulation onset sub-timing for a DMD micromirror within the modulation period *Λ*. This map also functions as the encryption key in SECAM. The generation of *O*(*i*, *j*) follows Algorithm S1 in the supplemental document. The period *Λ* is divided into *Q* sub-timings with interval *τ* = *Λ*/*Q*. Each micromirror (*i*, *j*) turns on at its designated sub-timing *O*(*i*, *j*) and remains on for *pτ* (typically *p* = *Q*/2) to allow IPE generation.

The asynchronous modulation is physically realized by cyclically displaying a sequence of binary patterns on the DMD. The *k*-th pattern is defined as:

3 $${P}_{k}(i,j)=\left\{\begin{array}{ll}1, & {{{\rm{if}}}}((O(i,j)-k+Q)\,{{{\rm{mod}}}}\,\,Q)\in \{0,1, \ldots ,p\,-\,1\},\\ 0, & {{{\rm{otherwise}}}}.\end{array}\right.$$

When the first pattern *P*_0_ is displayed, the DMD sends an anchor signal to the event camera, recording the reference timestamp *t*_*m*_ for the current modulation period. Combined with *O*(*i*, *j*) and *t*_*m*_, this enables precise determination of each mirror’s modulation onset. This process converts the absolute intensities of the scene into the response latencies of the resulting IPEs through optical encoding.

### Computational decoding

The raw asynchronous IPE stream is decoded into raw AsynTemMap frames through temporal synchronization and temporal-mapping. First, the temporal offsets *O*(*i*, *j*) are calibrated from the DMD to the event sensor coordinate system, producing ${O}^{{\prime} }(x,y)$ (detailed procedure in the Calibration of Event Camera and DMD Coordinates section of the supplemental document). The raw frames are then decoded following Algorithm 1, using ${O}^{{\prime} }(x,y)$ and anchor timestamps {*t*_*m*_}.

#### Algorithm 1

Raw AsynTemMap Frame Decoding

1: Input: Raw events {*e*_*i*_ = (*x*_*i*_, *y*_*i*_, *t*_*i*_)}; calibrated temporal offsets ${O}^{{\prime} }(x,y)$; anchor timestamps ${\{{t}_{m}\}}_{m=1}^{M}$

2: Output: Raw AsynTemMap frame sequence ${\{{A}_{m}\}}_{m=1}^{M}$

3: Synchronization: Adjust event timestamps by subtracting ${O}^{{\prime} }({x}_{i},{y}_{i})$ from *t*_*i*_: ${t}_{i}^{{\prime} }\leftarrow {t}_{i}-{O}^{{\prime} }({x}_{i},{y}_{i})$ for all *e*_*i*_.

4: **for** *m* = 1 to *M* **do**

5: Event selection: Select events ${{{{\mathcal{E}}}}}_{m}^{{\prime} }$ satisfying ${t}_{m}\le {t}_{i}^{{\prime} } < {t}_{m+1}$.

6: IPE extraction: For each pixel (*x*, *y*), first identify the event in ${{{{\mathcal{E}}}}}_{m}^{{\prime} }$ located at (*x*, *y*) that possesses the earliest timestamp ${t}_{i}^{{\prime} }$. Then, assign $T(x,y)\leftarrow {t}_{i}^{{\prime} }$.

7: Latency computation: Δt*(*x*, *y*) ← *T*(*x*, *y*) − *t*_*m*_

8: Intensity mapping: Convert Δt*(*x*, *y*) to intensity ${I}_{\max }(x,y)$ using Eq. (1).

9: Normalization: Normalize ${I}_{\max }(x,y)$ to obtain *A*_*m*_.

10: **end for**

The Synchronization step in Algorithm 1 serves as the essential decryption operation in SECAM, converting the temporally scrambled IPE stream into its physically meaningful form.

### Neural reconstruction

The proposed AsynTemRec employs the BasicVSR++^25^ architecture as its backbone for neural reconstruction from raw AsynTemMap frames. Its core innovation lies in explicitly incorporating the calibrated temporal offset map ${O}^{{\prime} }$ as a per-pixel positional embedding. This provides the model with precise knowledge of each pixel’s sampling timing within the modulation cycle, which is critical for correcting ASA. The Ablation Study section of the supplemental document demonstrate the essential role of ${O}^{{\prime} }$ in mitigating these artifacts, and the detailed network architecture and training procedures are also provided therein.

A comprehensive degradation model for raw AsynTemMap frames is established to handle real-world hardware degradations in AsynTemRec, expressed by:

4 $$A({{{\bf{x}}}})={{{{\mathcal{A}}}}}_{{O}^{{\prime} }}([(\Phi [G]\otimes H)({{{\bf{x}}}})\cdot V({{{\bf{x}}}})\cdot (1+T({{{\bf{x}}}}))]\cdot M({{{\bf{x}}}}))+{n}_{t}({{{\bf{x}}}}).$$

Here, the latent ground truth image *G* is affected not only by asynchronous sampling ${{{{\mathcal{A}}}}}_{{O}^{{\prime} }}(\cdot )$ but also by multiple hardware imperfections: geometric distortion (Φ), aberration-induced blur (*H*), and vignetting (*V*) from the optical system; fixed-pattern noise (*T*) and modulation defects (*M*) from the DMD; and Poisson-distributed temporal noise (*n*_*t*_) from the event sensor. These realistic degradations, calibrated using the procedures detailed in the AsynTemMap Noise and Degradation Model Calibration section of the supplemental document, are incorporated into the AsynTemRec training set. The effectiveness of this degradation modeling is further validated in the  Ablation Study section of the supplemental document.

### Statistics and reproducibility

PSNR and SSIM in Table 1 were computed using the functions from the skimage.metrics module in Python. LPIPS was computed using the lpips library with the Alex backbone. Evaluation was conducted on 10 test sequences, each containing 30-40 frames. All metrics were computed on a per-frame basis and then averaged over each sequence. The reported results correspond to the mean values across frames within each sequence, and the final average is computed across all sequences. No statistical tests were performed. Each experiment was conducted once per test sequence without repeated trials, as the evaluation is deterministic given fixed inputs and model parameters. Character recognition accuracy was evaluated using Tesseract-OCR software (version 5.5)^23^. All plots were generated using Matplotlib (version 3.3.4).

## Supplementary information

Transparent Peer Review file

Supplemental document

## Data Availability

All data supporting the findings of this study are publicly available at: 10.5281/zenodo.19480452.

## Code availability

The custom code used for data processing and evaluation is publicly available at: 10.5281/zenodo.19480452.
