# Supplementary material for: Asynchronous temporal mapping for high-dynamic-range video and hardware-level privacy with event cameras
Source: Commun Eng. 2026 May 13;5:136. doi: 10.1038/s44172-026-00687-4 (PMC13396165; doi:10.1038/s44172-026-00687-4)
Supplement: Supplementary file 2 — Supplemental document [file 44172_2026_687_MOESM2_ESM.pdf]

Capture Reality through a Confidential Eye: supplemental document

1. AsynTemMap System Setup

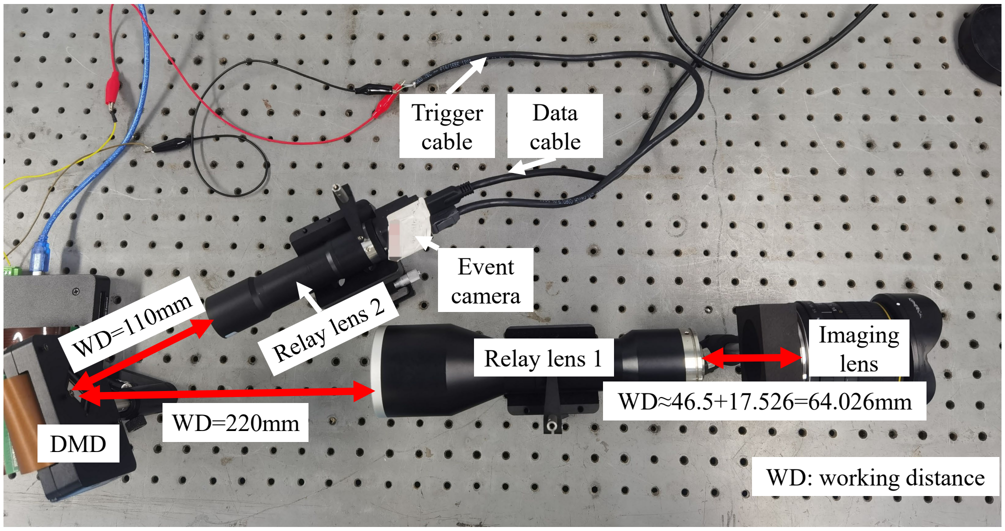

Fig. S1. Hardware Setup for the AsynTemMap System. The hardware setup consists of a Digital Micromirror Device (DMD), an event camera, two relay lenses, and an imaging lens. The image planes of the imaging lens, DMD, and event camera are conjugated through the two relay lenses. The event camera is connected to the DMD via a trigger cable to record the precise timestamps of DMD pattern switches.Key optical distances are labeled in red. The working distance between the imaging lens and Relay Lens 1, as labeled, is the sum of their respective back focal lengths (46.5 mm for the imaging lens and 17.526 mm for Relay Lens 1).

1.1. Overview of the AsynTemMap Hardware System

The AsynTemMap system employs an off-axis reflective optical path suitable for the Digital Micromirror Device (DMD), as shown in Fig. S1. The system incorporates a Prophesee EVK4 event camera with a resolution of 1280×720 and a pixel size of 4.86 µm, and a Texas Instruments DLP6500FLQ 0.65 1080p MVSP-A DMD, which supports binary transmittance modulation at up to 9.523 kHz. The relay optics consist of a VISION DATUM CLW-5MP-0.3X-110 and a COOLENS WWH08-110ATV2 lens, while the imaging lens is a Samyang 8mm f/3.5 UMC FISH-EYE CS II. To align with the DMD packaging, the event camera is rotated 45° around the optical axis.

1.2. Assembly Tips

For the assembly of the AsynTemMap system, a "back-to-front" approach is recommended, starting from relay lens 2 and proceeding toward the imaging lens.

- **Locate Event Camera and DMD:** Connect relay lens 2 to the event camera. Display an alternating all-black and multi-field-of-view focusing star pattern on the DMD. Adjust the distance and pose between relay lens 2 and the DMD until the flickering stars in all fields of view appear sharp on the event camera, indicating conjugation between the event camera's image plane and the DMD plane. Then, secure the event camera and DMD.
- **Locate relay lens 1:** Attach an auxiliary industrial camera to the image side of relay lens 1. While displaying the flickering star pattern on the DMD, adjust the distance and pose

between relay lens 1 and the DMD until the central field of view is sharp. *Note: Due to the off-axis configuration, relay lens 1 views a slanted surface, and the full field cannot be in focus simultaneously due to depth-of-field constraints.* Ensure the event camera's field of view overlaps with that of the auxiliary camera. Once aligned, remove the auxiliary camera and fix relay lens 1.

- **Locate imaging lens:** With all other components fixed and enclosed in a light-tight box, place a focusing star screen in the object plane of the imaging lens. Display alternating all-black and all-white patterns on the DMD. Adjust the imaging lens position until the event camera observes a sharp image of the central focusing stars.

1.3. Calibration of Event Camera and DMD Coordinates

To calibrate the coordinate mapping, place a light source (e.g., an iPad displaying a white screen) between relay lens 1 and the imaging lens to provide diffuse, even illumination across the field of view. Display an alternating checkerboard and all-black pattern on the DMD, allowing the event camera to accumulate a checkerboard event frame. The goal is to compute the homography between the original DMD checkerboard and the event-frame checkerboard. Since relay lens 2 is aligned to be normal to the DMD surface, the resulting homography should contain only rotation, translation, and scaling, without perspective distortion. This process also verifies the correct execution of the assembly steps. Note that distortion from relay lens 2 is negligible and can be ignored. Global, low-frequency non-uniformity in illumination is acceptable, as it does not affect the local extraction of checkerboard corners.

1.4. SECAM Encryption Key Generation Method

Optical encoding is implemented using a predefined temporal offset map $O(i, j)$, where each element specifies the modulation onset sub-timing for a DMD micromirror within the modulation period Λ. This map also functions as the encryption key in SECAM. The temporal offset map $O(i, j)$ is generated as follows:

Algorithm S1. Secure Temporal Offsets Key Generation

1: **Input:** image size $H \times W$; tile size w_s; seed; quantization range $(Q_{\min}, Q_{\max})$; acquisition period Λ
2: **Output:** temporal offsets $O \in \mathbb{Z}^{H \times W}$; modulation step τ
3: **Initialize:** Initialize two SHAKE-256 streams CTX_Q and CTX_M using seed to generate random values.
4: **Determine** Q**:** Sample Q uniformly from $[Q_{\min}, Q_{\max}]$ using CTX_Q and compute $\tau = \Lambda / Q$.
5: **Calculate grid size:** $S_h \leftarrow \lceil H/w_s \rceil, \quad S_w \leftarrow \lceil W/w_s \rceil$
6: **Generate grid offsets:** Use CTX_M to generate an i.i.d. stream of 64-bit integers $\{u_i\}$. Map each u_i to $[0, Q-1]$ and fill the grid $R \in \{0, \dots, Q-1\}^{S_h \times S_w}$.
7: **Expand to full resolution:** Upsample R by a factor of w_s using nearest-neighbor interpolation to obtain the full-resolution temporal offsets O.

This presents the secure temporal offsets key generation procedure, which generates secure offset maps using a seed and other hardware-related parameters. Key management is efficient—refreshing the seed instantly generates a new offset map, with only the seed needing storage or transmission. The quantization level Q, sampled from $[Q_{\min}, Q_{\max}]$, expands the key space and enhances security. The designed offsets $O(i, j)$ can be seen as the encryption key, while the calibrated offsets $O'(x, y)$ serve as the decryption key. The spatial relationship between them is fixed during system calibration. This static coordinate mapping functions as a persistent cipher component.

1.5. Relationship Between Modulation Frequency and Dynamic Range

Theoretically, a trade-off exists between the modulation frequency and the achievable dynamic range. The modulation period $T = 1/f$ fundamentally limits the maximum integration time available to detect an Initial Positive Event (IPE) from the darkest scene regions. Assuming the pixel reset time is sufficiently short, this period T defines the upper bound for the measurable latency. Therefore, the theoretical dynamic range (DR) can be expressed as:

$$\mathrm{DR}(f) = 20\log_{10}\left(\frac{I_{\max}}{I_{\min}(f)}\right) \propto 20\log_{10}\left(\frac{T_{\max}}{T_{\min}}\right) = 20\log_{10}\left(\frac{1/f}{T_{\min}}\right), \tag{S1}$$

where $T_{\min}$ is the minimum detectable latency, determined by the temporal resolution of the event camera. Current state-of-the-art event cameras offer a typical temporal resolution of 1 µs. This $T_{\min}$ value critically defines the highlight capture capability. A lower $T_{\min}$ significantly enhances the system's ability to resolve finer intensity gradations in bright regions, thereby pushing the $I_{\max}$ boundary further.

2. AsynTemMap Noise and Degradation Model Calibration

As detailed in the main text, the observed AsynTemMap frame is affected not only by asynchronous sampling artifacts (ASA) but also by a combination of hardware-induced degradations. These include geometric distortion $\Phi[\cdot]$, aberration-induced blur H, vignetting $V(\mathbf{x})$, fixed-pattern noise (FPN) texture $T(\mathbf{x})$, micromirror-jitter-induced dropouts $M(\mathbf{x})$, and Poisson-distributed timestamp jitter $n_t(\mathbf{x})$. The following sections describe the calibration procedures for each component to enable accurate synthesis of training data and high-quality reconstruction.

2.1. Geometric Distortion

Geometric distortion $\Phi[\cdot]$ originates primarily from the imaging lens, an 8mm fisheye lens, while the two telecentric relay lenses contribute negligible distortion.

We calibrate the distortion using a standard checkerboard pattern placed in the object plane. With the DMD set to synchronous modulation (switching between all-black and all-white patterns), we capture a grayscale image of the checkerboard via EvTemMap. The intrinsic parameters and distortion coefficients are then estimated using `cv2.calibrateCamera`, yielding a distortion model K. This model is applied to undistort all reconstructed frames—including those from AsynTemMap and E2VID—before quantitative comparison with the ground truth.

2.2. Aberration-induced Blur

A significant source of aberration in our setup stems from the tilted surface of the DMD within the off-axis optical path. To maintain pixel-level modulation accuracy, relay lens 1 is oriented obliquely to conjugate with the DMD, while relay lens 2 remains normally aligned. This configuration causes the image formed by the imaging lens to project onto a tilted plane at the DMD, resulting in a defocus that is most pronounced at the image edges.

This aberration-induced blur H represents a system-level limitation that equally affects AsynTemMap and E2VID, as all three methods share the same optical front-end. To ensure a fair comparison in the main experiments, we do not explicitly model or compensate for this specific blur within the AsynTemRec degradation model. Its presence is acknowledged here as a contributing factor to the overall deviation from the ground truth. Should future work require more precise aberration compensation, the modulation transfer function (MTF) could be calibrated via the checkerboard edge method and approximated with a Gaussian point spread function (PSF), following methods like [1].

2.3. Vignetting and FPN Texture

We calibrate the vignetting $V(\mathbf{x})$ and FPN texture $T(\mathbf{x})$ using a sequence of flat-field images captured under uniform illumination. The overall calibration results are shown in Fig. S2. This calibration requires a high-quality, spatially uniform Lambertian source (e.g., an integrating sphere) to establish a reliable reference for the system's intrinsic photometric response. It is a one-time procedure that characterizes fixed optical and DMD properties; the stringent uniformity condition is therefore both necessary and acceptable for obtaining an accurate degradation model. The procedure is as follows:

- **Flat-field Image Acquisition:** Capture multiple frames under uniform illumination to record the system's global response, encompassing both vignetting and FPN. A representative flat-field image is shown in Fig. S2(a).

- **Vignetting Calibration:** Vignetting manifests as a low-frequency intensity falloff from the image center. We estimate $V(\mathbf{x})$ by applying a Gaussian filter (kernel size $\sigma = 51$ pixels) to the average flat-field image, extracting the low-frequency attenuation map. The calibrated vignetting $V(\mathbf{x})$, showing normalized light attenuation, is visualized in Fig. S2(b).

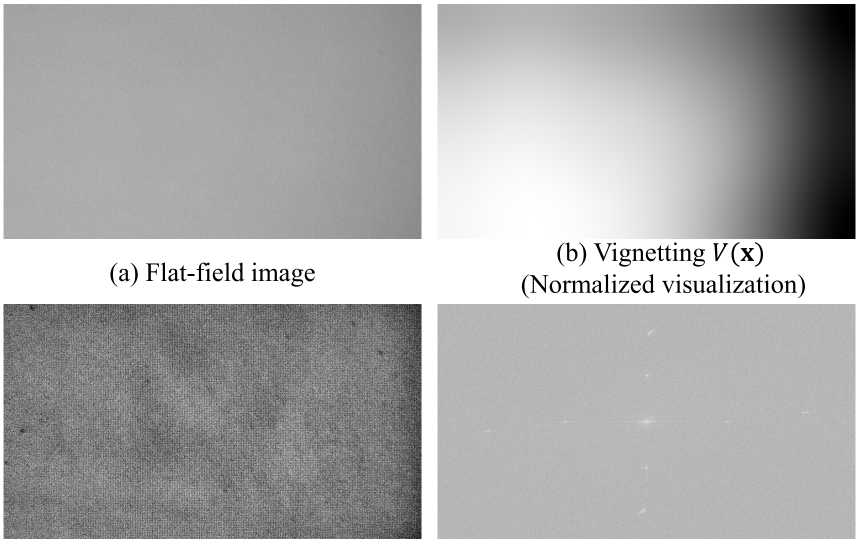

(a) Flat-field image

(b) Vignetting $V(\mathbf{x})$ (Normalized visualization)

(c) FPN Texture $T(\mathbf{x})$ (Normalized visualization)

(d) Fourier Transform of $T(\mathbf{x})$

Fig. S2. Calibration results for vignetting $V(\mathbf{x})$ and FPN texture $T(\mathbf{x})$. (a) Flat-field image captured under uniform illumination. (b) Calibrated vignetting $V(\mathbf{x})$, showing normalized light attenuation. (c) FPN texture $T(\mathbf{x})$ obtained after vignetting subtraction. **(d)** Fourier transform of the FPN texture, revealing its frequency-domain characteristics.

- **FPN Calibration:** The FPN texture $T(\mathbf{x})$ is isolated by subtracting the calibrated vignetting component $V(\mathbf{x})$ from the average flat-field image. The result is a high-frequency residue representing the fixed-pattern noise inherent to the DMD and sensor, as shown in Fig. S2(c). The Fourier transform of the FPN texture (Fig. S2(d)) reveals its frequency-domain characteristics, showing that the FPN is dominated by a periodic grid structure, manifested as local peaks in the frequency domain. This structure essentially arises from the regular array and edge effects of the individual micromirrors on the DMD surface.

The calibrated maps $V(\mathbf{x})$ and $T(\mathbf{x})$ are incorporated into the synthetic data generation pipeline, ensuring that the AsynTemRec model is trained under realistic, hardware-aware degradation conditions.

2.4. Micromirror-Jitter-Induced Dropouts

Due to the imperfect pixel-to-micromirror correspondence between the DMD and the event camera, certain micromirrors located near the boundaries of modulation tiles may partially span across two adjacent event-camera pixels during asynchronous modulation. As a result, these boundary micromirrors generate pseudo-jitter events simultaneously in both pixels. Such spurious events differ from genuine Initial Positive Events (IPEs) and occur frequently, degrading the local intensity accuracy of AsynTemMap reconstruction if retained.

To mitigate this effect, we perform a dedicated calibration procedure to identify and mask the affected pixels. A uniformly illuminated plane is placed at the object plane of the AsynTemMap system. Under ideal asynchronous modulation, all pixels should exhibit identical IPE generation latencies. We compute the per-pixel deviation between the measured IPE latency and the global mean latency. Pixels exhibiting significantly larger deviations are identified as being affected by micromirror jitter and are permanently masked during data decoding. This process produces the spatial dropout mask $M(\mathbf{x})$, which explains the characteristic crack-like grayscale discontinuities observed in raw AsynTemMap frames. This calibration requires the illumination to be sufficiently uniform such that the latency variation induced by illumination non-uniformity is substantially smaller than the deviation caused by micromirror jitter. This ensures that the mask $M(\mathbf{x})$ accurately captures the hardware defect rather than being contaminated by scene-dependent lighting artifacts.

2.5. Poisson-Distributed Jitter

Photon shot noise, also referred to as Poisson-distributed jitter, originates from the discrete and stochastic nature of photon arrival events at each pixel [2]. While the following derivation is formally based on the integrative imaging model of conventional CMOS sensors, the same underlying physics applies to our temporal mapping imaging principle, where it similarly gives rise to Poisson-distributed noise.

Under constant illumination, the number of photoelectrons $N_e(\mathbf{x})$ accumulated during an exposure time t follows a Poisson distribution with expectation $\lambda_e(\mathbf{x})$:

$$N_e(\mathbf{x}) \sim \text{Poisson}(\lambda_e(\mathbf{x})). \tag{S2}$$

Here, $\lambda_e(\mathbf{x})$ denotes the expected number of photoelectrons, which is proportional to both the local irradiance and the exposure duration.

After photoelectric conversion via the sensor gain g, the resulting digital signal $I_t(\mathbf{x})$ is given by

$$I_t(\mathbf{x}) = \frac{1}{g} N_e(\mathbf{x}) + n_r(\mathbf{x}), \tag{S3}$$

where $n_r(\mathbf{x})$ represents additive electronic readout noise, typically modeled as a zero-mean Gaussian process independent of the photon statistics.

The stochastic component attributable to photon shot noise is thus defined as

$$n_t(\mathbf{x}) = \frac{1}{g} \left[N_e(\mathbf{x}) - \lambda_e(\mathbf{x}) \right], \tag{S4}$$

with a variance that scales linearly with the mean signal intensity:

$$\text{Var}\left[n_t(\mathbf{x}) \right] = \frac{1}{g^2} \lambda_e(\mathbf{x}) \propto I_t(\mathbf{x}). \tag{S5}$$

This statistical behavior leads to the well-known variance–mean relation commonly observed in flat-field calibration:

$$\text{Var}(I_t) \approx a \cdot \text{Mean}(I_t) + b, \tag{S6}$$

where the slope $a = g^{-1}$ quantifies the photon shot noise contribution, and the intercept b captures the variance of the readout noise.

It is crucial to note that in the context of event-based temporal mapping imaging, this noise manifests directly as jitter in the IPE response latency, causing fluctuations in event timestamps. However, for the sake of practical calibration simplicity, we do not directly measure this jitter in the temporal domain. Instead, by analogy to the CMOS integrative model, we model and calibrate the Poisson-distributed noise directly in the *intensity domain* by following the variance–mean relation described above.

3. Implementation Details of Reconstruction Model

3.1. Model Overview

For static scenes, AsynTemMap faithfully matches the ground truth; however, dynamic content gives rise to a unique asynchronous sampling artifact (ASA), as shown in Fig. S3(a). This artifact manifests as misaligned features in moving regions due to the intrinsic asynchrony of our sampling scheme, where pixels are captured at different sub-timings. The combination of ASA and hardware-induced degradations necessitates a dedicated reconstruction step. Accordingly, we propose AsynTemMap Video Reconstruction (AsynTemRec), a model that corrects misalignments, fills dropouts, and suppresses noise. As shown in Fig. S3(b), AsynTemRec successfully restores high-quality frames by eliminating feature misalignment around the cyclist and inpainting pixels with missing grayscale.

Figure S3(c) outlines the reconstruction pipeline. The key modules are as follows: **Flow Estimate.** Coarse optical flow is first estimated between consecutive raw AsynTemMap frames to initialize the motion field. **Feature Extract.** Raw AsynTemMap frames $\{A\}$ are channel-wise concatenated with temporal offsets O', from which spatiotemporal features of asynchronous grayscale patches are extracted. Here, O' provides per-pixel positional embeddings, explicitly indicating the specific sampling time of each patch, providing crucial timing cues to the reconstruction model. **Flow Refine.** The initial flow is refined using the extracted features, yielding a

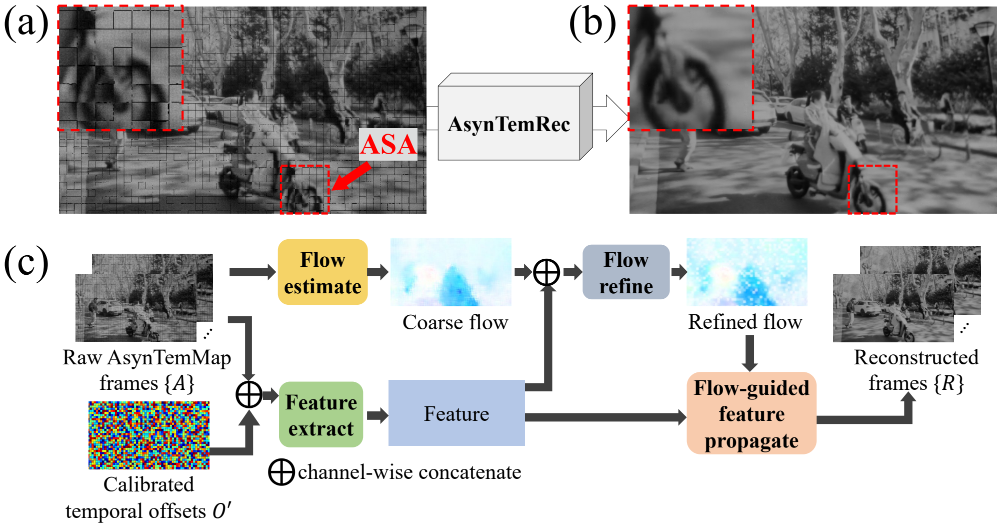

Fig. S3. **(a)** Raw AsynTemMap frame in a dynamic scene exhibiting asynchronous sampling artifact (ASA) and hardware-induced dropouts. **(b)** Reconstructed frame using AsynTemMap Video Reconstruction (AsynTemRec), where ASA is removed, dropouts are restored, and noise is suppressed. **(c)** Overview of the reconstruction pipeline.

motion field that aligns patches sampled at different sub-timings to their corresponding positions at the anchor moment t_m. **Flow-Guided Feature Propagate.** The refined flow guides feature warping and aggregation across space-time, enabling coherent reconstruction of frames at $\{t_m\}$. The implementation details are provided in the following section. AsynTemRec builds on the flow-guided propagation framework of BasicVSR++[3] and incorporates defect-aware refinement from video inpainting models[4, 5] to address hardware-induced degradations. We adapt these methods to address ASA—an artifact distinct from conventional ones such as motion blur or occlusion. Central to our approach is the incorporation of temporal offsets O' as positional embeddings within the reconstruction model. By supplying precise sampling-time information, AsynTemRec realigns features across sub-timings. Thus, temporal offsets bridge optical encoding, computational decoding, and neural reconstruction, highlighting the value of a unified physical model in the computation optical imaging framework.

3.2. Module Architecture

AsynTemRec is built upon the BasicVSR++ [3] backbone, a recurrent bidirectional video restoration framework. We retain its propagation and reconstruction modules while introducing two major modifications: (i) a feature extraction module that incorporates temporal offset embeddings, and (ii) a dedicated flow refinement module for correcting motion estimation under asynchronous sampling. Figure S4 illustrates the modified architecture, with detailed configurations of each component listed below.

Flow Estimate. We employ a lightweight SpyNet [6] variant for initial optical flow estimation, balancing accuracy with computational efficiency to suit high-frame-rate AsynTemMap inputs. This module produces a two-channel flow field at a reduced spatial resolution, which is then refined by the subsequent flow refinement module.

Feature Extract. As illustrated in Fig. S4(a), this module takes AsynTemMap frames concatenated with their corresponding temporal offsets as input. The calibrated offset map O' is normalized to the range $[0, 1]$ and provided as channel-wise positional embeddings. As a result, the input channels of the first convolutional layer are increased from 1 (grayscale) to 2 (grayscale + temporal offset embedding). This design enables the network to explicitly incorporate pixel-wise sampling time information during the feature extraction process.

Flow Refine. As detailed in Fig. S4(b), this module consists of a series of residual blocks that process the concatenation of the initial optical flow and features from the **Feature Extract** module. The refined flow output maintains the same spatial resolution as the input flow.

Flow-Guided Feature Propagate. Feature propagation follows the bidirectional recurrent strategy of BasicVSR++ [3]. Forward and backward features are warped according to the refined flow

a

Layer	**Feature Extract**
1	Conv(2,64,3)
2	LeakyReLU(0.1)
3	Conv(64,64,3)
4	LeakyReLU(0.1)
5	Conv(64,64,3)
6	LeakyReLU(0.1)
7~11	ResidualBlockNoBN

b

Layer	**Flow Refine**
1	Conv(64+2,64,3)
2	ReLu
3	ResidualBlock
4	Conv(64,32,3)
5	ReLu
6	Conv(32,2,3)

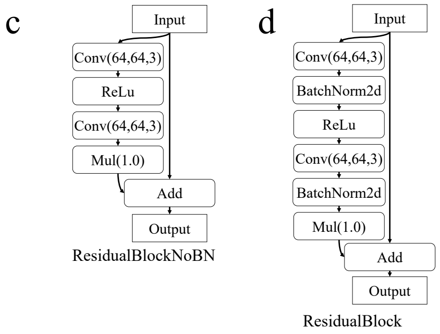

Fig. S4. Architecture of the modified modules in AsynTemMap. (a) The **Feature Extract** module. Each convolution layer is annotated as Conv(input channels, output channels, kernel size). The slope for negative inputs in the LeakyReLU activation is set to 0.1. (b) The **Flow Refine** module. (c) Structure of the **ResidualBlockNoBN**, which omits batch normalization. **(d)** Structure of the standard **ResidualBlock**, which includes batch normalization. Modules not explicitly shown (e.g., **Flow Estimate** and **Flow-Guided Feature Propagate**) adhere to the original BasicVSR++ design [3].

fields and fused using gated aggregation.

3.3. Training Objective

The AsynTemRec network is trained in a supervised manner to reconstruct ground-truth grayscale anchor frames $\{Y_m\}$ from the corresponding raw AsynTemMap inputs $\{A_m\}$. The reconstruction quality is optimized using the Charbonnier loss, a differentiable variant of the ℓ_1 loss that is robust to pixel-level outliers introduced by noise and slight misalignments in the data. The loss function is defined as:

$$\mathcal{L}_{\text{rec}} = \frac{1}{N} \sum_{i=1}^{N} \sqrt{(x_i - y_i)^2 + \epsilon^2}, \tag{S7}$$

where x_i and y_i denote the predicted and ground-truth pixel intensities, respectively, N is the number of valid pixels, and ϵ is a small constant (set to 10^{-12}) for numerical stability.

This objective function effectively penalizes reconstruction errors while maintaining resilience against pixel-level inaccuracies stemming from hardware-induced noise. We deliberately avoid additional perceptual or adversarial losses, as our primary goal is to produce visually faithful and radiometrically accurate reconstructions that preserve the true scene content, rather than prioritizing perceptual sharpness that may not correspond to physical reality.

3.4. Training Schedule and Hyperparameters

All experiments, including the ablation studies, are trained for 200k iterations with a batch size of 1 and a sequence length of 10. Each input patch has a spatial resolution of 256×256. The training is conducted on a single NVIDIA RTX 4090 GPU and takes approximately one day to complete.

The model is optimized using the Adam optimizer with an initial learning rate of 1×10^{-4}, using betas of (0.9, 0.99). A Cosine Restart learning rate schedule is applied for the entire 200k iterations, decaying the learning rate to a minimum of 1×10^{-7}. The total number of trainable parameters is approximately 9.89 million, and all training is implemented in PyTorch 1.10.2 with the MMEditing framework.

3.5. Inference Details

During inference, each input is a raw AsynTemMap sequence consisting of 35–40 consecutive frames with a spatial resolution of 1280×720. Inference is performed on a single NVIDIA RTX

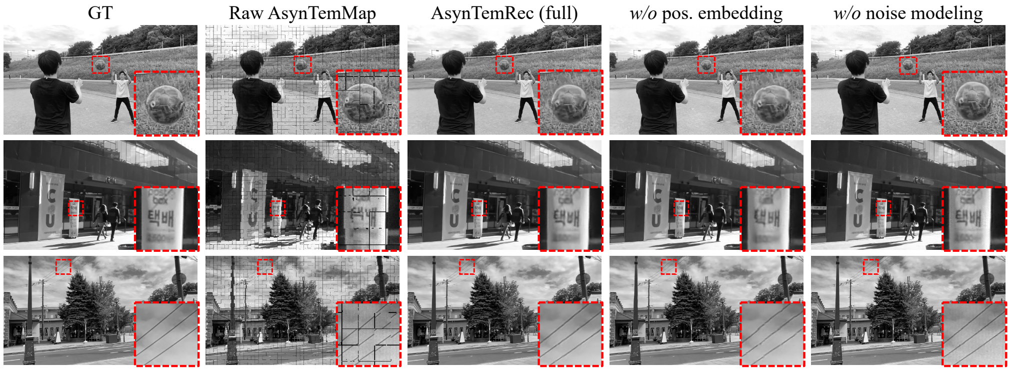

Fig. S5. Qualitative ablation study on the synthetic dataset. Red boxes highlight representative regions of interest. Raw AsynTemMap frames exhibit pronounced hardware-induced degradations and Asynchronous Sampling Artifacts (ASA). The full AsynTemRec model effectively mitigates both effects, producing high-quality reconstructions. Removing positional embeddings results in noticeable feature deformation in regions affected by ASA, while omitting noise modeling leads to reconstructions that retain considerable artifacts and spurious textures.

4090 GPU, achieving an average runtime speed of approximately 3.12 frames per second. Each raw sequence is reconstructed end-to-end to ensure consistency with real-time AsynTemMap video decoding. The peak GPU memory consumption during inference is approximately 6.3 GB.

3.6. Ablation Study

3.6.1. Ablation Configurations

To demonstrate the importance of temporal offsets as positional embeddings and the necessity of degradation modeling—as emphasized in the main text—we conduct an ablation study for validation. All model variants are trained under identical conditions—using the same number of iterations, datasets, schedules, and hyperparameters as the full model—to ensure a fair and conclusive comparison.

Raw AsynTemMap. This baseline represents the raw imaging quality prior to any learning-based processing. It directly converts the synchronized IPE timestamps into grayscale frames without applying our reconstruction network.

AsynTemRec (full). This is our complete proposed model. It incorporates both the calibrated degradation modeling during training and the temporal-offset positional embeddings at the input, enabling it to effectively handle practical hardware degradations and ASA.

w/o Positional Embedding. In this variant, the temporal offset map input O' is replaced by an all-zero matrix, effectively removing timing information from the network input. This configuration tests whether AsynTemRec can still correct ASA in the absence of explicit temporal alignment cues.

w/o Noise Modeling. In this variant, no calibrated noise is injected into the training dataset. Specifically, the Poisson-distributed jitter $n_t(\mathbf{x})$, vignetting $V(\mathbf{x})$, and FPN texture $T(\mathbf{x})$ are excluded during training. Specifically, the geometric distortion $\Phi[\cdot]$ is retained during evaluation to ensure a fair quantitative comparison with the full model. In addition, the spatial dropout mask $M(\mathbf{x})$ is preserved to maintain the basic inpainting functionality of the AsynTemMap framework.

Table S1. Ablation study on real and synthetic datasets.

Real Dataset				*Synthetic Dataset*			
Method	PSNR↑	SSIM↑	LPIPS↓	Method	PSNR↑	SSIM↑	LPIPS↓
Raw AsynTemMap	15.89	0.2663	0.7255	Raw AsynTemMap	15.70	0.3981	0.6117
AsynTemRec (full)	**21.42**	**0.6222**	**0.4313**	AsynTemRec (full)	**29.86**	**0.9128**	**0.1010**
w/o positional embedding	21.36	0.6183	0.4408	*w/o* positional embedding	28.28	0.8798	0.1299
w/o degradation modeling	20.94	0.5808	0.5122	*w/o* degradation modeling	28.34	0.8623	0.1377

3.6.2. Quantitative Comparison

Table S1 summarizes the ablation results, evaluating the impact of positional embeddings and degradation modeling on both real and synthetic datasets. On the real dataset, removing positional embeddings leads to a minor performance drop, whereas omitting degradation modeling causes a more pronounced decline (PSNR -0.48 dB, SSIM -0.041, LPIPS $+0.081$). This indicates that degradation modeling is crucial for robust real-world performance, especially when ASA is obscured by stronger distortions such as optical aberrations H. In contrast, the synthetic dataset—which excludes aberration-induced blur H while preserving other degradations and noise—clearly reveals the impact of ASA. Here, positional embeddings are critical: their removal results in substantial degradation across all metrics (PSNR -1.6 dB, SSIM -0.033, LPIPS $+0.029$). The ablation study confirms that temporal offsets O' act as a unifying variable throughout the imaging pipeline—guiding not only optical encoding but also computational decoding and deep reconstruction. This underscores the importance of co-designing optics and algorithms in computational optical imaging.

3.6.3. Qualitative Comparison

Figure S5 presents a qualitative visualization of ablation study on the synthetic dataset, providing visual evidence of each component's contribution. We select three representative sequences, each exhibiting pronounced ASA and characteristic hardware degradations.

In the first sequence, a rapidly moving ball exhibits severe asynchronous sampling artifacts (ASA). The full AsynTemRec model successfully restores both the spherical shape and surface texture of the ball. In contrast, when positional embeddings are ablated (*w/o* Pos. Embedding), the reconstructed ball appears noticeably elongated and deformed, a direct consequence of missing temporal alignment cues. On the other hand, omitting degradation modeling (*w/o* Noise Modeling) leads the network to misinterpret fixed-pattern noise as authentic scene texture, introducing structured artifacts across the ball's surface.

The second sequence, which contains fine textual details, further highlights the role of positional embeddings: their removal causes clear character distortion and blurring, as the model cannot accurately align asynchronously captured patches. When noise modeling is excluded, residual hardware-induced artifacts persist around the character boundaries, indicating that the model fails to distinguish true text structure from noise-corrupted regions.

In the third sequence, featuring thin power lines against a uniform sky, the absence of positional embeddings breaks the continuity of the wires, demonstrating that ASA correction remains inadequate. Simultaneously, the absence of noise modeling results in unsuppressed fixed-pattern noise in the sky, amplifying FPN into perceptually distracting patterns that compromise the radiometric cleanliness of smooth regions.

4. Encryption Characteristics under Hardware Constraints

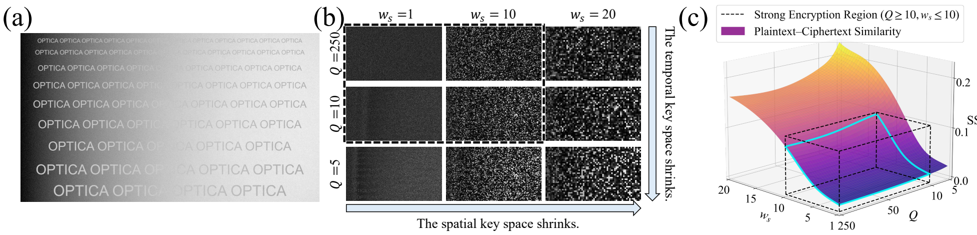

Fig. S6. Analysis of encryption characteristics under hardware constraints. (a) Plaintext image of characters against a gradient background. (b) Resulting SECAM ciphertext under different combinations of encryption parameters: temporal quantization level Q and spatial tile size w_s. (c) Structural similarity (SSIM) between the ciphertext and plaintext, quantifying the encryption strength across different parameter sets.

As established in the main text, SECAM provides significant advantages over software-based encryption in terms of security and computational overhead. In this section, we examine its intrinsic encryption characteristics under practical hardware constraints. The encryption strength of SECAM is governed by two key parameters: the temporal quantization level Q and the spatial tile size w_s, which define the modulation granularity achievable by the physical system.

To evaluate the sensitivity of these parameters, we conduct a simulation using a character text overlaid on a grayscale gradient as the plaintext (Fig. S6(a)). The resulting ciphertexts under different Q and w_s values are displayed in Fig. S6(b), with their structural similarity (SSIM) to the plaintext quantified in Fig. S6(c). This analysis reveals several critical trends: First, encryption effectiveness diminishes significantly when $w_s > 10$. In our experiment, the smallest characters occupy approximately 17×17 pixels, meaning that tile sizes larger than 10 pixels cover more than half of a character, leaving visible partial structures. Second, when $Q < 10$, plaintext-ciphertext similarity increases sharply, as coarse temporal quantization fails to sufficiently disrupt the IPE latency distribution. Third, details in dark regions are more vulnerable under small Q values. This is because, according to the inverse latency-intensity relationship, details in darker regions are more dispersed in the temporal domain. When subjected to equally spaced temporal scrambling, the encryption effect in dark areas is weaker than in brighter areas when mapped to the intensity domain.

From the quantitative analysis in Fig. S6(c), we observe that SSIM remains below 0.1—indicating strong encryption—when $Q \geq 10$ and $w_s \leq 10$. We now assess these thresholds against practical hardware limitations:

- The maximum achievable Q is constrained by the modulation frequency of the spatial light modulator. For instance, with an acquisition period of $\Lambda = 20$ ms (equivalent to 50 FPS), a modulator running at 1 kHz supports $Q = 20$, which is well above the security threshold of 10.

- The minimum feasible w_s is bounded by the effective resolution of the modulation system. For a sensor with 1280×720 pixels, a modulator with a resolution exceeding 128×72 ensures $w_s < 10$, again satisfying the encryption requirement.

Such specifications are readily supported by modern Digital Micromirror Device (DMD)-based spatial light modulators. This confirms that SECAM can deliver strong encryption within the performance envelope of currently available hardware, demonstrating that its security is not only theoretically sound but also practically achievable.

REFERENCES

1. S. Chen, H. Feng, K. Gao, *et al.*, "Extreme-quality computational imaging via degradation framework," in *Proceedings of the IEEE/CVF International Conference on Computer Vision (ICCV),* (2021), pp. 2632–2641.
2. J. R. Janesick, *Photon Transfer*, vol. v.PM170 (SPIE, Bellingham, 2007), 1st ed.
3. K. C. Chan, S. Zhou, X. Xu, and C. C. Loy, "Basicvsr++: Improving video super-resolution with enhanced propagation and alignment," in *Proceedings of the IEEE/CVF conference on computer vision and pattern recognition,* (2022), pp. 5972–5981.
4. S. Zhou, C. Li, K. C. Chan, and C. C. Loy, "Propainter: Improving propagation and transformer for video inpainting," in *Proceedings of the IEEE/CVF international conference on computer vision,* (2023), pp. 10477–10486.
5. H. Shi, Y. Li, K. Yang, *et al.*, "Fishdreamer: Towards fisheye semantic completion via unified image outpainting and segmentation," in *Proceedings of the IEEE/CVF Conference on Computer Vision and Pattern Recognition (CVPR) Workshops,* (2023), pp. 6434–6444.
6. A. Ranjan and M. J. Black, "Optical flow estimation using a spatial pyramid network," in *Proceedings of the IEEE conference on computer vision and pattern recognition,* (2017), pp. 4161–4170.
