# Supplementary material for: Asynchronous temporal mapping for high-dynamic-range video and hardware-level privacy with event cameras
Source: Commun Eng. 2026 May 13;5:136. doi: 10.1038/s44172-026-00687-4 (PMC13396165; doi:10.1038/s44172-026-00687-4)
Supplement: Supplementary file 1 — Transparent Peer Review file [file 44172_2026_687_MOESM1_ESM.pdf]

nature portfolio

Peer Review File

Asynchronous Temporal Mapping for High-Dynamic-Range Video and Hardware-Level Privacy with Event Cameras

Corresponding Author: Professor Kaiwei Wang

This file contains all reviewer reports in order by version, followed by all author rebuttals in order by version.

Version 0:

Reviewer comments:

Reviewer #1

(Remarks to the Author)
This paper attempts to address two challenges in conventional imaging systems: limited dynamic range and visual privacy leakage. Traditional cameras directly capture absolute scene intensity, making them vulnerable under extreme lighting conditions and prone to privacy leakage. Event cameras offer advantages over traditional RGB cameras by recording only relative intensity changes, although they struggle to recover the absolute scene intensity. To address these limitations, the authors propose asynchronous temporal mapping (AsynTemMap) to encode the real scene irradiance into temporal delay of the triggered events and using a neural network-based image recovery. This design allows for the raw event stream to be obfuscated during the acquisition. The authors conduct experiments to validate the proposed work. While these experiments are promising, there are some concerns regarding the framework itself, which I discuss below.

Strengths of the paper:
+ The paper shows strategies for effective bandwidth management by distributing event generation over time across pixels, allowing for high frame rate video acquisition
+ The neural reconstruction pipeline enables jointly compensating for misaligned sampling artifacts and hardware-induced imperfections, and shows strong image reconstruction performance.
+ The claimed hardware-level encryption implemented by modulating the sensing process is interesting. The results seem to indicate that the cipher text achieves security metrics that are comparable to digital encryption methods.

Major comments:

Although the results are promising, the authors seem to overstate the overall contributions. The authors use the term "confidential eye" to claim two capabilities: faithful HDR capture and privacy protection. HDR imaging with event cameras has been a well studied aspect, which includes the author provided references 13 and 14. The authors do not seem to improve over these methods, but largely alleviate the bandwidth limitations in high frame-rate video acquisition, which seems slightly orthogonal to the claim. On a minor note, this aspect of the paper is more of an engineering advance rather than a fundamental one.

Regarding the privacy protection, the analysis and comparisons with digital encryption methods are not sufficient to support the strong claim of confidentiality. If the claim is privacy and security, then a comprehensive security analysis needs to be provided, which includes a clear threat model, consideration of practical attack scenarios (e.g., chosen plaintext attack, known plaintext attack, side channel attack, etc.), and the proposed method's robustness under such attack scenarios. For instance, imagine the DMD modulation patterns are cyclic; what happens if someone inserts a uniform (e.g. all-white) frame into the video sequence? Or what happens if there are cyclic structural information in the video sequence? (same problem like ECB penguin). Without such analysis it is difficult to analyze the security of the camera, and the contribution may appear overstated.

Minor comments:
- Can you clarify if the temporal-mapping image of Fig. 2 is synchronous or asynchronous?
- In subfigure b and d of the Fig. 3, I suggest to use the same color to represent the same metric.
- This work uses a DMD to modulate the imaging process. As DMD patterns are updated globally, does this introduce bandwidth issues like the event camera?
- In the neural reconstruction section,

a) I suggest to clarify if the ProPainter is finetuned on the capturing dataset, otherwise it is unfair to compare with a training

method.
b) For E2VID baseline, it isn't suitable to use 'on' state to act as no-modulation because there will be hardware imperfections (mentioned by the authors).
c) It would be better to compare with a newer baseline rather than E2VID, and provide more video visualizations in the result section.
d)Please clarify if the network is trained and tested with the dataset consist of the same key or different key.

- As the authors claim that temporal misalignment error and hardware imperfections are solved by the neural reconstruction, is it really faithful image? If that is, what makes the E2VID reconstruction not a faithful image?
- Please provide the ground truth for Fig. 5.

Overall, the motivation for the work is well received and the topic is interesting and timely. However, the manuscript lacks sufficient analysis on privacy, and the claimed contributions appear to be somewhat overstated. I believe a major revision is needed to make the manuscript strong and to be considered for publication.

Reviewer #2

(Remarks to the Author)
In this manuscript by Bao et al., the authors present Asynchronous Temporal Mapping (AsynTemMap), an innovative computational imaging framework that integrates optical encoding, computational decoding, and neural reconstruction to achieve high-dynamic-range (HDR) video acquisition with built-in hardware-level privacy protection. The work is timely, addressing two critical challenges in DMD-based event imaging: extreme illumination handling with data-bandwidth overload and visual data confidentiality. The system is well-designed, thoroughly validated, and represents a significant advance in event-based vision. The paper is clearly written, well-structured, and supported by strong experimental results. Overall, I recommend acceptance pending minor revisions.

(a)In fact, the optical encryption property is a common feature in many computational imaging paradigms. This is because, during the intermediate process of optical encoding, it is often visually inaccessible. For instance, in the framework of DMD-based single-pixel imaging, only one-dimensional time fluctuation signals will be obtained after optical encoding. Without knowing the optical encoding (or illumination mode), it is impossible to obtain the spatial information of the target.
(b)Using spatially random latency as a key is interesting, but it is very easy to be detected or even be eavesdropped on. Therefore, the authors should discuss the degree of difficulty in inferring the correspondence or the key between the original scene patterns and the encrypted patterns when the eavesdropper has obtained both sets of information. Moreover, it seems that such a computational imaging framework may not be compatible with 3D imaging systems based on time-of-flight.
(c)There is no doubt that the time cost for encryption and decryption is extremely low. The time cost of neural reconstruction process should be clearly stated in the main text.

Reviewer #3

(Remarks to the Author)
This manuscript presents a comprehensive AsynTemMap system encompassing optical design, encoding-decoding algorithms, neural reconstruction, security encryption, and detailed calibration procedures. The topic is suitable for Communications Engineering. Detailed comments are as follows.

1. The main text mentions that conventional event streams leak contour information, but it does not provide a deep comparison between SECAM and existing hardware or algorithmic encryption schemes specifically designed for event cameras
2. Only a theoretical value of 114 dB is given. Quantitative comparison data on measured dynamic range and signal-to-noise ratio (SNR) between AsynTemMap and existing event-camera HDR imaging methods (e.g., [14]) in standard HDR scenes needs to be added.
3. The metrics in Table 2 only evaluate visual obfuscation. It 's better to add a brief analysis of potential resistance to known-plaintext attacks and chosen-plaintext attacks, alongside a discussion of the leakage risk of the time offset map O(i,j) under side-channel attacks (e.g., power analysis, timing analysis).
4. Figure 4 and the main text acknowledge AsynTemRec's limited recovery of fine, low-contrast wires in the "church" scene. I suggest adding an in-depth analysis to determine whether this is a limitation of the network architecture/loss function or a fundamental drawback of asynchronous sampling, along with suggestions for improvement.
5. In the hardware schematic, consider labeling key distances or focal lengths of optical components.
6. Clarify the dependence of some calibration steps on uniform illumination assumptions and their applicable conditions.

Version 1:

Reviewer comments:

Reviewer #2

(Remarks to the Author)
The author has made sufficient revisions. I recommend publishing it in the current version.

Reviewer #3

(Remarks to the Author)
Most of my previous concerns have been addressed. Some minor comments:
1. The revision reports AsynTemRec inference speed (198.82 ms/frame ≈ 5 fps on RTX 4090) and frames it as offline, high-quality reconstruction. However, wording such as ``real-time privacy-preserving vision / strict real-time performance" may be interpreted as end-to-end real-time reconstruction. Please explicitly separate real-time encryption/decryption/decoding from non-real-time neural reconstruction, and keep the abstract/conclusion consistent.
2. Please add a one-sentence clarification (main text or supplement): whether 115 dB refers to the scene illuminance dynamic range (measured by lux meter) or the reconstructable system dynamic range (derived from noise/threshold/temporal resolution). The current wording reads closer to the former.

Open Access This Peer Review File is licensed under a Creative Commons Attribution 4.0 International License, which permits use, sharing, adaptation, distribution and reproduction in any medium or format, as long as you give appropriate credit to the original author(s) and the source, provide a link to the Creative Commons license, and indicate if changes were made.
In cases where reviewers are anonymous, credit should be given to 'Anonymous Referee' and the source.
The images or other third party material in this Peer Review File are included in the article's Creative Commons license, unless indicated otherwise in a credit line to the material. If material is not included in the article's Creative Commons license and your intended use is not permitted by statutory regulation or exceeds the permitted use, you will need to obtain permission directly from the copyright holder.
To view a copy of this license, visit https://creativecommons.org/licenses/by/4.0/

Response Letter for COMMSENG-25-0908-T
Capture Reality through a Confidential Eye

Yuhan Bao, Hao Shi, Jiajun Zhai , and Kaiwei Wang

Quick Navigation for Reviewers

- Reviewer 1 & 2 & 3: Major Concerns on Privacy Protection and Security Analysis
- Reviewer 1:
  - Concerns on Contribution Positioning and Overstatement
  - Minor Comments of Reviewer 1
- Reviewer 2: Additional Comments from Reviewer 2
- Reviewer 3: Additional Comments from Reviewer 3

The authors of the paper would like to thank the Editors and Reviewers for their timely handling of our manuscript and useful feedback. They have helped us improve our paper in several aspects, for which we are really grateful. We provide this document as the response to the previous review comments and the revision of the submitted manuscript ("Capture Reality through a Confidential Eye", ID COMMSENG-25-0908-T).

As instructed, the paper has been modified and extended according to the Reviewers' comments. Details on the introduced changes and specific answers to the queries are reported in this document. Please notice that, in this report, the comments from the Reviewers are shown in Comments; the feedback from the authors is in the Response; the extended texts are shown in Added; the revised texts are shown in Revised and highlighted in green.

I Major Concerns on Privacy Protection and Security Analysis

I.1 Overview and Scope of the Security Analysis

Comments

Reviewer 1 major comment 2: Regarding the privacy protection, the analysis and comparisons with digital encryption methods are not sufficient to support the strong claim of confidentiality. If the claim is privacy and security, then a comprehensive security analysis needs to be provided, which includes a clear threat model, consideration of practical attack scenarios (e.g., chosen plaintext attack, known plaintext attack, side channel attack, etc.), and the proposed method's robustness under such attack scenarios. . . .

Reviewer 2 comment (b): Using spatially random latency as a key is interesting, but it is very easy to be detected or even be eavesdropped on. Therefore, the authors should discuss the degree of difficulty in inferring the correspondence or the key between the original scene patterns and the encrypted patterns when the eavesdropper has obtained both sets of information. . . .

Reviewer 3 comment 3: The metrics in Table 2 only evaluate visual obfuscation. It 's better to add a brief analysis of potential resistance to known-plaintext attacks and chosen-plaintext attacks, alongside a discussion of the leakage risk of the time offset map O(i,j) under side-channel attacks (e.g., power analysis, timing analysis).

Response

We thank the reviewers for raising important concerns regarding the security and privacy claims of SECAM. In the revised manuscript, we have substantially expanded the security analysis by adding a dedicated section (3.4. Threat model and attack analysis) that systematically examines the privacy protection capability of SECAM under different adversarial models.

Specifically, Section 3.4.1 introduces a clear threat model and defines the adversarial capabilities considered in this work, including ciphertext-only, known-plaintext, and chosen-plaintext attack scenarios, thereby addressing the reviewer 1' request for an explicit security scope.

Section 3.4.2 analyzes potential information leakage available to a ciphertext-only adversary, with a particular focus on timing-related side-channel information arising from the DMD modulation process. This analysis evaluates whether statistical patterns in the encrypted event stream could be exploited without access to the plaintext or the secret key.

Section 3.4.3 investigates the system behavior under known-plaintext conditions by quantifying the degree of information leakage when partial plaintext–ciphertext pairs are available to an adversary. This section is designed to address the reviewers' con-

cerns on key inference and correspondence recovery in realistic observation scenarios.

Finally, Section 3.4.4 discusses the implications of chosen-plaintext attacks, in which an adversary may actively inject controlled scene content (e.g., uniform or structured inputs). While such attacks represent a stronger and less realistic threat model for practical deployment, we analyze potential vulnerabilities and discuss mitigation strategies such as randomized key rotation to limit long-term exposure.

Together, these additions provide a comprehensive and structured security analysis that complements the visual obfuscation metrics in the original manuscript and clarifies the scope and limitations of the claimed privacy protection. In the following subsections, we respond in detail to each security-analysis-related comment raised by the reviewers, with explicit references to the corresponding revisions in the manuscript.

Added

3.4 . Threat model and attack analysis

3.4.1. Threat model definition

In this section, we define the threat model considered for the security analysis of SECAM and outline three types of adversaries with progressively increasing capabilities: a ciphertext-only attacker, a known-plaintext attacker (KPA), and a chosen-plaintext attacker (CPA). The ciphertext-only attacker represents the weakest adversary, who has access only to the encrypted event stream without any knowledge of the original scene or the encryption key. Under this setting, the attacker can only exploit statistical or side-channel information from the ciphertext itself. We evaluate SECAM under this threat model through side-channel analyses. The known-plaintext attacker is assumed to have access to both the ciphertext and partial plaintext information. This setting reflects more practical attack scenarios where limited scene content may be known or inferred. We assess SECAM under this threat model via quantitative leakage analysis and demonstrate its advantage over 2D-NVS. The chosen-plaintext attacker represents the strongest adversary, with the ability to actively select scene inputs and observe the corresponding ciphertext. Rather than claiming formal security against this attack, we discuss the potential risks of SECAM under a naive hardware configuration and outline feasible engineering mitigation strategies in. Together, these three threat models provide a structured framework for analyzing the security properties of SECAM, from passive observation to increasingly powerful adversarial capabilities.

3.4.2. Ciphertext-only attacker: side-channel analysis

...

3.4.3. Known-plaintext attacker (KPA) analysis on a periodic pattern

...

1.4.4. Discussion on chosen-plaintext attacker (CPA)

. . .

I.2 Side-Channel Analysis

Comments

Reviewer 1 major comment: . . . which includes a clear threat model, consideration of practical attack scenarios (e.g., chosen plaintext attack, known plaintext attack, side channel attack, etc.), and the proposed method's robustness under such attack scenarios. . . .

Reviewer 3 comment 3: . . . It 's better to add a brief analysis of potential resistance to known-plaintext attacks and chosen-plaintext attacks, alongside a discussion of the leakage risk of the time offset map O(i,j) under side-channel attacks (e.g., power analysis, timing analysis).

Response

To address the reviewers' concerns regarding side-channel leakage under a ciphertext-only attack model, we added Section 3.4.2 to explicitly analyze the statistical information available to an adversary who can only observe the encrypted event stream. The detailed analysis and experimental results are provided in the revised manuscript shown below.

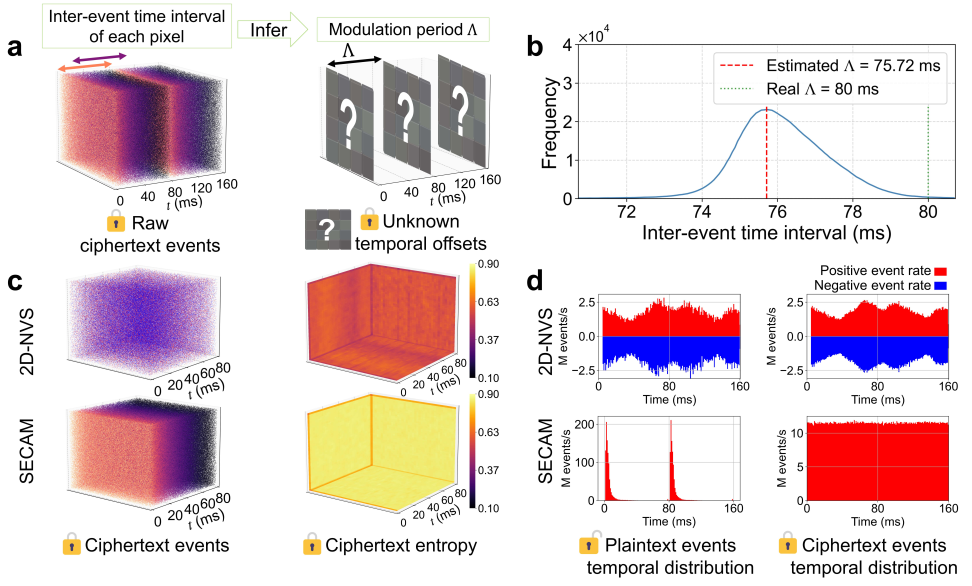

Figure 6: Side-channel analysis experiment conducted by the Ciphertext-only attacker. a The ciphertext events of SECAM have certain time repeatability under the periodic modulation of DMD. The DMD modulation period Λ can be inferred from the time interval when each pixel generates events, which is a fixed system property independent of the visual scene content. b Event time interval analysis of the ciphertext captured by SECAM to estimate the DMD modulation period Λ. Due to the influence of trailing events, the Λ estimated from this analysis is smaller than the true value. c Spatiotemporal entropy analysis of the ciphertext for both 2D-NVS and SECAM. The results show that SECAM's entropy is consistently higher than that of 2D-NVS, exhibiting stronger statistical randomness. Additionally, 2D-NVS's entropy significantly decreases between 40 and 60ms. d Further analysis of the temporal distribution characteristics of the ciphertext for both 2D-NVS and SECAM. 2D-NVS almost fully exposes the temporal distribution information of the plaintext, allowing the attacker to infer motion and scene texture changes. In contrast, SECAM's ciphertext is evenly distributed in time, demonstrating resilience to statistical analysis.

Added

3.4.2. Ciphertext-only attacker: side-channel analysis

For a ciphertext-only attacker, besides brute-force decryption, the only available attack vector is to exploit the statistical information inherent in the ciphertext. We refer to this as side-channel analysis.

For the SECAM paradigm, due to the cyclic modulation of the DMD, the ciphertext inherently contains periodic repetition characteristics, especially when capturing a static or slowly varying scene. This periodicity presents an initial opportunity for the

ciphertext-only attacker to perform side-channel analysis. Theoretically, under the fixed modulation period Λ of the DMD, the time intervals between positive events generated at the same position should correspond to Λ, as schematically illustrated in Fig. 6a. To investigate this, we extract approximately 160ms of raw IPEs from the face sequence and compute the time intervals between consecutive positive events for each pixel. The distribution of these inter-event time intervals is shown in Fig. 6b. A clear peak is observed in the histogram, where the peak position corresponds to an estimated $\Lambda = 75715\,\mu s$, which is smaller than the actual DMD modulation period of 80ms. This discrepancy arises because, after each modulation onset by the DMD, in addition to generating IPEs, a small number of trailing events are randomly generated during the subsequent period. Therefore, the estimated Λ, based on the inter-event time interval peak, is smaller than the true Λ. This shows that the hardware-induced noise from trailing events provides some level of resistance to side-channel attacks on SECAM.

In the subsequent experiment, we assume that the ciphertext-only attacker has successfully inferred the correct Λ by other means and has obtained the corresponding ciphertext for a frame. The attacker proceeds to analyze the statistical distribution characteristics of the ciphertext in order to extract features of the plaintext. In Fig. 6c, we compare the spatiotemporal characteristics of raw IPEs captured by SECAM with those of motion events encrypted using 2D-NVS. This comparison evaluates the statistical properties of the ciphertexts produced by the two encryption paradigms operating on their respective native data types. To quantify the randomness of the ciphertext event distribution, we employ the normalized spatiotemporal entropy:

$$H(i,j,k) = \begin{cases} \dfrac{-\sum_{\Omega} \frac{n_{\Omega}}{N} \log \frac{n_{\Omega}}{N}}{\log M}, & N > 0 \\ 0, & N = 0 \end{cases} \tag{1}$$

where $H(i,j,k)$ denotes the normalized local spatiotemporal entropy centered at voxel (i,j,k). Events are first discretized into a spatiotemporal grid with voxel dimensions of 8 pixels (spatial) and 1.0 ms (temporal). The summation in the entropy calculation is performed over all voxels Ω within a $K_X \times K_Y \times K_T$ neighborhood centered at (i,j,k), where we set $K_X = K_Y = K_T = 5$. This corresponds to a local spatiotemporal volume containing $M \triangleq K_X K_Y K_T = 125$ voxels. Here, n_{Ω} is the number of events in voxel Ω, and $N = \sum_{\Omega} n_{\Omega}$ is the total number of events in that neighborhood. A value of $H(i,j,k)$ closer to 1 indicates a more uniform spatiotemporal distribution of events within the local volume, implying weaker statistical structure and thus enhanced resistance to side-channel analysis of the ciphertext. As shown in Fig. 6c, the entropy of the SECAM ciphertext is consistently higher than that of 2D-NVS, demonstrating that SECAM produces ciphertext with reduced statistical regularities and thus enhanced resistance to side-channel attacks.

A more detailed comparison of the ciphertext entropy in Fig. 6c reveals that SECAM ciphertext exhibits uniform entropy projections along the X, Y, and T axes, while

the entropy of 2D-NVS ciphertext shows a noticeable drop between 40 and 60ms. This suggests that there might be a vulnerability in the temporal distribution characteristics of 2D-NVS ciphertext. To explain this phenomenon, we further compare the event rate over time for both plaintext and ciphertext of 2D-NVS and SECAM in Fig. 6d. It can be seen that the event distribution characteristics of 2D-NVS plaintext and ciphertext are similar, and the event rate decreases between 40 60ms, indicating a reduction in scene motion or texture. The temporal vulnerability of 2D-NVS allows the ciphertext-only attacker to analyze motion or scene changes from the event stream. In contrast, SECAM's ciphertext events are uniformly distributed in time, effectively masking the temporal distribution features of the plaintext, making it difficult for a ciphertext-only attacker to conduct statistical analysis for side-channel attacks.

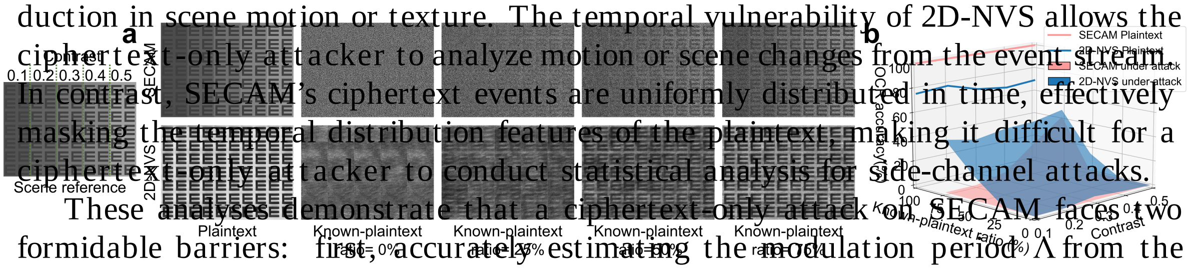

These analyses demonstrate that a ciphertext-only attack on SECAM faces two formidable barriers: first, accurately estimating the modulation period Λ from the noisy ciphertext is difficult in practice; second, and more critically, the statistical uniformity of the ciphertext—evidenced by its consistently high spatiotemporal entropy and temporally flat distribution—provides no leverage for reconstructing the visual plaintext even if Λ were known. Thus, the core security mechanism of SECAM successfully decouples the ciphertext from the plaintext in both space and time. Consequently, this fundamental spatiotemporal decorrelation ensures that statistical analysis of the ciphertext alone yields negligible information about the original scene, robustly fulfilling the requirement for confidentiality against ciphertext-only adversaries.

I.3 Known-plaintext Attack Analysis

Reviewer 1 major comment: . . . which includes a clear threat model, consideration of practical attack scenarios (e.g., chosen plaintext attack, known-plaintext attack, side channel attack, etc.), and the proposed method's robustness under such attack scenarios. . . .

Reviewer 2: . . . Therefore, the authors should discuss the degree of difficulty in inferring the correspondence or the key between the original scene patterns and the encrypted patterns when the eavesdropper has obtained both sets of information. . . .

Reviewer 3 comment 3: . . . It 's better to add a brief analysis of potential resistance to known-plaintext attacks and chosen-plaintext attacks, alongside a discussion of the leakage risk of the time offset map O(i,j) under side-channel attacks (e.g., power analysis, timing analysis).

Response

To address the reviewers' concerns regarding known-plaintext attacks and key/correspondence inference when partial plaintext information is available, we added Sec-

tion 3.4.3 to systematically evaluate information leakage under known-plaintext attack (KPA) scenarios. The detailed experimental design, quantitative results, and visual comparisons are provided in the revised manuscript and reproduced below for the reviewers' convenience.

Figure 7: Comparison of resistance to known-plaintext attacks between SECAM and 2D-NVS [1] event encryption. a Reconstructed ciphertext images of character 'E' under varying scene contrasts (0.1–0.5) and known-plaintext ratios: SECAM leaks high-contrast features only at high known-plaintext ratios, while 2D-NVS exhibits consistent leakage regardless of contrast. b Optical character recognition (OCR) accuracy as a quantitative metric of information leakage: SECAM achieves substantially lower ciphertext OCR accuracy than 2D-NVS across all attack conditions, demonstrating stronger confidentiality. The 100% plaintext OCR accuracy for SECAM confirms faithful reconstruction of the original scene—establishing a valid baseline for security evaluation—whereas 2D-NVS's degraded plaintext accuracy (< 80%) stems from reconstruction distortion, not enhanced privacy.

Added

3.4.3. Known-plaintext attacker (KPA) analysis on a periodic pattern

In event-based encryption, a known-plaintext attack (KPA) assumes the adversary possesses partial knowledge of the plaintext event stream and attempts to infer visual content from the reconstructed grayscale image. To realistically evaluate information leakage under such threats, we design an experiment that varies both the known-plaintext ratio (simulating the attacker's prior knowledge) and the scene contrast (reflecting real-world imaging conditions). We use a spatially periodic pattern of the character 'E' as a controlled test pattern and employ the open-source Tesseract-OCR software [2] to quantitatively measure optical character recognition (OCR) accuracy on both plaintext reconstructions and ciphertext reconstructions. This provides a principled, task-driven benchmark for visual information leakage across encryption schemes. We compare two event encryption approaches: SECAM IPEs reconstructed with AsynTemRec, and 2D-NVS [1] encrypted motion events reconstructed with E2VID [3].

Fig. 7a displays the plaintext and corresponding ciphertext images reconstructed

under varying known-plaintext ratios, with SECAM results shown in the top row and 2D-NVS in the bottom row. For each contrast level, we capture one dataset (SECAM raw IPEs and, separately, raw motion events for 2D-NVS input). The images shown are excerpts from reconstructions performed independently at different known-plaintext ratios and then stitched together for visualization. Fig. 7b quantifies information leakage via OCR accuracy across scene contrast levels (0.1–0.5) and known-plaintext ratios: solid curves represent plaintext OCR accuracy (100% known events), while colored surfaces depict ciphertext OCR accuracy under partial knowledge.

Plaintext comparison. When the full plaintext is known (rightmost column of Fig. 7a), SECAM's reconstruction faithfully reproduces the ground-truth character 'E' across all contrast levels. This high-fidelity reconstruction is confirmed by the 100% OCR accuracy for all contrasts (red curve in Fig. 7b), establishing a valid baseline for security evaluation. In contrast, the plaintext reconstructions for 2D-NVS (i.e., motion events reconstructed with E2VID) appear nearly identical across contrast levels and exhibit noticeable geometric distortion, resulting in a plaintext OCR accuracy below 80% (blue curve). The underlying reasons for this reconstruction fidelity gap have been analyzed in depth in Section 3.2.

Ciphertext analysis. When only the ciphertext is available (known-plaintext ratio = 0%), SECAM's output appears chaotic and shows no discernible periodic structure corresponding to the repeated 'E' pattern, regardless of contrast. This confirms that the spatially random key $O'(x, y)$ effectively provides diffusion in the spatial domain, thereby preventing ECB-penguin-like leakage. In contrast, 2D-NVS ciphertext exhibits a concerning consistency across contrast levels: its reconstructed images appear very similar for different contrast scenes. This occurs because the raw motion events generated by the character 'E' at various contrast levels are inherently similar. More importantly, the 2D-NVS algorithm processes these similar plaintext streams through a deterministic cryptographic process. Consequently, similar plaintexts lead to statistically similar ciphertexts, resulting in nearly identical reconstructions. This allows statistical patterns from the plaintext to persist in the ciphertext, a behavior reminiscent of the well-known weakness in the ECB encryption mode [4].

Partial known-plaintext analysis. As the known-plaintext ratio increases, SECAM exhibits contrast-dependent leakage: higher-contrast instances become visually discernible first. The lowest-contrast character (0.1) remains largely unrecognizable until the known-plaintext ratio reaches 75%. Quantitatively, at 75% known-plaintext ratio, the OCR accuracy for the highest-contrast character (0.5) reaches only 40% and drops sharply with decreasing contrast or known-plaintext ratio (red surface in Fig. 7b). In contrast, 2D-NVS shows consistent information leakage across all contrast levels, with ciphertext OCR accuracy consistently higher than SECAM's under the same attack conditions (blue surface). This indicates greater vulnerability to known-plaintext attacks.

These results demonstrate that SECAM provides effective resistance to known plaintext attacks while maintaining high-fidelity reconstruction capability, whereas 2D-NVS exhibits higher information leakage under the same attack conditions. This

divergence stems from the fundamentally different types of event data they encrypt. SECAM operates on intensity-encoded events that retain absolute photometric information. Its encryption scrambles pixel-level intensity values, which inherently imposes stronger perturbation on low-contrast, weak-signal features. This property directly enhances privacy: the pixel-wise intensity scrambling imposed by SECAM's encryption disproportionately affects low-contrast features (e.g., watermarks, fine facial textures). As a result, even with partial known-plaintext, such sensitive content remains effectively shielded from leakage. In contrast, 2D-NVS encrypts motion-triggered events that reflect scene edges and texture. Due to the high redundancy of these events in representing structural content, even a partial set of known events often enables an adversary to infer the dominant contours of the scene. Consequently, this encryption paradigm offers inherently weaker privacy protection: low-contrast features either fail to generate sufficient events to faithfully represent the underlying scene, or—once encoded—are just as vulnerable to reconstruction from partial known-plaintext as high-contrast ones.

I.4 Chosen-plaintext Attack (Active Injection Attacks) Analysis

Comments

Reviewer 1 major comment: ...which includes a clear threat model, consideration of practical attack scenarios (e.g., chosen plaintext attack, known plaintext attack, side channel attack, etc.), and the proposed method's robustness under such attack scenarios. ...

Reviewer 3 comment 3: ...It 's better to add a brief analysis of potential resistance to known-plaintext attacks and chosen-plaintext attacks, alongside a discussion of the leakage risk of the time offset map O(i,j) under side-channel attacks (e.g., power analysis, timing analysis).

Reviewer 1: ...For instance, imagine the DMD modulation patterns are cyclic; what happens if someone inserts a uniform (e.g., all-white) frame into the video sequence? ...

Response

To address the reviewers' concerns regarding chosen-plaintext attacks (CPA) and active information injection, we added Section 3.4.4 to explicitly discuss the practical implications of CPA for SECAM.

In particular, our analysis is directly motivated by the example raised by Reviewer 1, where an adversary injects a spatially uniform (e.g., all-white) frame into the video sequence to probe the system. We show that such an attack could, in principle, be used to infer the temporal offset map $O'(x, y)$ if a static key were reused over an extended period.

Importantly, we clarify that mounting this type of CPA requires a strong adver-

sarial capability with direct control over the optical input, which exceeds the passive or remote eavesdropping threat model considered in most deployment scenarios. Nevertheless, for completeness, we analyze this worst-case setting and demonstrate that simple and low-overhead mitigation strategies—such as frequent and randomized key renewal—can effectively eliminate long-term leakage even under active injection attempts.

The detailed discussion and analysis are provided in the revised manuscript and reproduced below for the reviewers' convenience.

Added

3.4.4. Discussion on chosen-plaintext attacker (CPA)

A chosen-plaintext attacker (CPA) possesses the capability to actively choose input patterns to the imaging system and observe the corresponding ciphertexts, constituting a powerful attack model that typically assumes a high degree of access to or control over the target. For SECAM, mounting a practical CPA would generally require direct physical access to the hardware to precisely inject controlled optical patterns, a scenario that extends beyond our defined threat model of a remote or passive eavesdropper. Nevertheless, we discuss the potential risks and mitigation strategies for completeness.

The most effective CPA against SECAM would involve injecting a spatially uniform, high-intensity light pulse at a known time t_{inj}. Under such a condition, the IPE generation latency Δt^* for every pixel approaches its minimum, causing the recorded event timestamps to cluster near $t_{inj} + O'(x,y)$. An adversary could then analyze the temporal offsets in the ciphertext event stream to statistically infer the spatial key map $O'(x,y)$.

The primary risk arises if the same static key $O'(x,y)$ is used indefinitely across a long capture session. To mitigate this, we emphasize that SECAM's security in operational settings does not rely on long-term key secrecy alone. As demonstrated by the high key sensitivity (NPCR $\approx$ 99%) in Table 2, even a minor change in the key completely alters the ciphertext. This property enables a straightforward and effective countermeasure: frequent key renewal. By randomly regenerating and reloading the DMD offset map $O'(x,y)$, any partially recovered key information becomes obsolete. The computational and operational overhead for this renewal is negligible, as it involves only reloading a precomputed pattern to the DMD.

Therefore, while a classical CPA with extensive hardware access poses a theoretical challenge, the proposed system can be robustly secured against such advanced threats in practice through simple key management. This maintains the confidentiality of video streams without compromising the efficiency and hardware-native advantages of the SECAM paradigm.

I.5 Analysis Under Periodic and Repetitive Scene Structures

Comments

Reviewer 1: ... Or what happens if there are cyclic structural information in the video sequence? (same problem like ECB penguin)...

Response

We thank Reviewer 1 for raising the concern regarding cyclic or repetitive structural information in the video sequence and its potential analogy to the ECB penguin problem. In the revised manuscript, we explicitly address this concern by analyzing two distinct forms of periodicity: temporal repetition and spatial structural repetition.

First, we consider temporal periodicity arising from static or slowly varying scenes under the cyclic DMD modulation. As analyzed in Section 3.4.2, such periodicity may allow a ciphertext-only adversary to infer the DMD modulation period Λ via side-channel analysis of inter-event timing. Importantly, we clarify that the inferred modulation period reflects only a fixed system parameter and does not encode scene-dependent statistical information. Consequently, while temporal repetition may expose system-level timing characteristics, it does not lead to visual information leakage.

Second, we analyze spatially repetitive structures using a controlled periodic scene pattern in the known-plaintext attack analysis (Section 3.4.3). This experiment directly evaluates whether repeated spatial structures in the plaintext could lead to ECB-like leakage in the ciphertext. The results show that SECAM effectively disrupts spatial periodicity through pixel-wise intensity scrambling, producing ciphertext reconstructions that exhibit no discernible repetitive structure even when the plaintext contains strong spatial regularity. In contrast, we observe that 2D-NVS exhibits consistent ciphertext patterns for similar periodic inputs, revealing an ECB-penguin-like vulnerability due to both the redundancy of motion events and the deterministic processing of similar plaintexts.

Together, these analyses demonstrate that SECAM is resilient to both temporally and spatially repetitive scene structures: temporal periodicity reveals only system-level parameters without scene leakage, while spatial repetition is effectively obfuscated, preventing ECB-like information exposure. The detailed analyses are provided in Sections 3.4.2 and 3.4.3 of the revised manuscript.

Added

3.4.2. Ciphertext-only attacker: side-channel analysis

... For the SECAM paradigm, due to the cyclic modulation of the DMD, the ciphertext inherently contains periodic repetition characteristics, especially when capturing a static or slowly varying scene. This periodicity presents an initial opportunity for the ciphertext-only attacker to perform side-channel analysis. Theoretically, under the fixed modulation period Λ of the DMD, the time intervals between positive events

generated at the same position should correspond to Λ, as schematically illustrated in Fig. 6a. To investigate this, we extract approximately 160ms of raw IPEs from the face sequence and compute the time intervals between consecutive positive events for each pixel. The distribution of these inter-event time intervals is shown in Fig. 6b. A clear peak is observed in the histogram, where the peak position corresponds to an estimated $\Lambda = 75715\,\mu s$, which is smaller than the actual DMD modulation period of 80ms. This discrepancy arises because, after each modulation onset by the DMD, in addition to generating IPEs, a small number of trailing events are randomly generated during the subsequent period. Therefore, the estimated Λ, based on the inter-event time interval peak, is smaller than the true Λ. This shows that the hardware-induced noise from trailing events provides some level of resistance to side-channel attacks on SECAM. . . .

3.4.3. Known-plaintext attacker (KPA) analysis on a periodic pattern

. . . We use a spatially periodic pattern of the character 'E' as a controlled test pattern and employ the open-source Tesseract-OCR software [2] to quantitatively measure optical character recognition (OCR) accuracy on both plaintext reconstructions and ciphertext reconstructions. . . .

Ciphertext analysis. When only the ciphertext is available (known-plaintext ratio = 0%), SECAM's output appears chaotic and shows no discernible periodic structure corresponding to the repeated 'E' pattern, regardless of contrast. This confirms that the spatially random key $O'(x, y)$ effectively provides diffusion in the spatial domain, thereby preventing ECB-penguin-like leakage. In contrast, 2D-NVS ciphertext exhibits a concerning consistency across contrast levels: its reconstructed images appear very similar for different contrast scenes. This occurs because the raw motion events generated by the character 'E' at various contrast levels are inherently similar. More importantly, the 2D-NVS algorithm processes these similar plaintext streams through a deterministic cryptographic process. Consequently, similar plaintexts lead to statistically similar ciphertexts, resulting in nearly identical reconstructions. This allows statistical patterns from the plaintext to persist in the ciphertext, a behavior reminiscent of the well-known weakness in the ECB encryption mode [4]. . . .

I.6 Compatibility with 3D Time-of-Flight Imaging Systems

Comments

Reviewer 2: . . . Moreover, it seems that such a computational imaging framework may not be compatible with 3D imaging systems based on time-of-flight. . . .

Response

We thank Reviewer 2 for raising the question regarding the compatibility of AsynTemMap with 3D time-of-flight (ToF) imaging systems. We clarify that the current AsynTemMap framework is not designed to be directly integrated with ToF systems, as the two paradigms encode fundamentally different physical information in the temporal domain: AsynTemMap maps scene irradiance to event timing, whereas ToF systems encode depth information via photon time-of-flight.

Nevertheless, we note that these two approaches operate on markedly different temporal scales. AsynTemMap typically introduces intensity-dependent temporal delays on the order of milliseconds, while ToF systems measure depth using time delays on the order of microseconds. In principle, this separation in temporal bandwidth suggests that a joint design may be feasible by allocating distinct temporal ranges to irradiance encoding and depth measurement.

A rigorous integration of AsynTemMap with ToF-based 3D imaging would require careful co-design of the sensing hardware and timing architecture, which is beyond the scope of the present work and constitutes an interesting direction for future research. We emphasize that this limitation does not affect the core contributions of the paper, which focus on high-dynamic-range event video acquisition and hardware-level privacy protection for intensity-based visual sensing.

II Concerns on Contribution Positioning and Overstatement

Comments

Reviewer 1: Although the results are promising, the authors seem to overstate the overall contributions. The authors use the term ”confidential eye” to claim two capabilities: faithful HDR capture and privacy protection. HDR imaging with event cameras has been a well studied aspect, which includes the author provided references 13 and 14. The authors do not seem to improve over these methods, but largely alleviate the bandwidth limitations in high frame-rate video acquisition, which seems slightly orthogonal to the claim. On a minor note, this aspect of the paper is more of an engineering advance rather than a fundamental one.
Reviewer 2: Only a theoretical value of 114 dB is given. Quantitative comparison data on measured dynamic range and signal-to-noise ratio (SNR) between AsynTemMap and existing event-camera HDR imaging methods (e.g., [14]) in standard HDR scenes needs to be added.

Response

We thank Reviewer 1 for the insightful comments regarding potential overstatement of the contributions. We would like to clarify our positioning and the corresponding

revisions made in the manuscript.

First, regarding the term "confidential eye," we emphasize that this claim is intentionally retained. In the revised manuscript, rather than weakening this claim, we substantially strengthen its technical foundation by adding a dedicated security analysis section (Section 3.4). This new section provides a systematic evaluation of SECAM under ciphertext-only, known-plaintext, and chosen-plaintext attack models, directly addressing the reviewers' concerns on privacy protection. We therefore believe that the "confidential eye" concept is now more rigorously justified through explicit threat modeling and quantitative analysis, rather than relying solely on visual obfuscation.

Second, we agree with Reviewer 1 that high-fidelity HDR imaging with event cameras is not an independent contribution of this work. As clarified in the revised manuscript, faithful HDR reconstruction is a core contribution of our prior work EvTemMap [5], and we do not claim AsynTemMap to fundamentally advance HDR reconstruction quality beyond existing event-based HDR methods. Accordingly, we do not position AsynTemMap as a new HDR imaging benchmark and therefore do not include exhaustive quantitative comparisons of dynamic range or SNR against prior HDR-focused event-camera methods (e.g., [6]), as requested by Reviewer 2. Instead, HDR fidelity in this work is treated as an inherited property from the EvTemMap framework and serves as a baseline to highlight the bandwidth bottleneck and video acquisition limitations that AsynTemMap is designed to address. In response to Reviewer 1's comment, we have deliberately toned down the HDR-related contribution statements in both the Abstract and Introduction.

Nevertheless, we retain the EvTemMap-related experimental results in the manuscript for two specific reasons. First, these results serve to highlight the inherent limitations of synchronous temporal-mapping imaging, particularly the severe bandwidth bottleneck under high-speed modulation and the resulting difficulty in achieving continuous video acquisition. Second, they provide essential background for readers who may not be familiar with our prior work, offering necessary context for understanding temporal-mapping-based imaging before introducing AsynTemMap.

Accordingly, the primary contribution of AsynTemMap is repositioned as addressing the bandwidth-limited setting of prior synchronous temporal-mapping approaches, enabling high-fidelity video acquisition under extreme illumination and motion conditions. To reflect this revised positioning, we have (i) refined the contribution statements in the Abstract, (ii) restructured the narrative logic in the Introduction to emphasize bandwidth-aware asynchronous modulation, and (iii) reorganized the Results section by merging the original Sections 3.1 and 3.2 into a new Section 3.1 entitled "From Snapshot to Video: Overcoming the Bandwidth Bottleneck." This revised presentation treats HDR capability as an inherited property from EvTemMap rather than a standalone contribution of AsynTemMap.

We hope these clarifications and revisions adequately address the concern regarding contribution overstatement while accurately reflecting the system-level advances introduced in this work.

Revised

Abstract

Conventional imaging systems are challenged to reconcile the competing demands of capturing reality under extreme illumination and ensuring visual data remain confidential from the moment of acquisition. We present Asynchronous Temporal Mapping (AsynTemMap), a framework that encodes high-dynamic-range visual information into a chaotic, asynchronous event stream through pixel-wise optical modulation. AsynTemMap addresses the bandwidth bottleneck of prior synchronous temporal-mapping approaches, enabling high-fidelity video capture with event cameras. By embedding the asynchronous principle of event sensing into a computational optical pipeline, AsynTemMap forges a new visual paradigm—one that captures reality with high fidelity and inherently protects it at the hardware level.

Introduction

...Among them, temporal-mapping approaches [5, 6] attempt to overcome the intensity ambiguity of event cameras by globally modulating optical transmittance and decoding absolute intensity from event timing, thereby enabling high-fidelity snapshot HDR imaging. However, their synchronous, global modulation schemes suffer from a fundamental bottleneck for video acquisition: at the high modulation rates required for video, it triggers near-simultaneous events across the entire sensor. This creates bursty event explosions that saturate the sensor's data bandwidth, leading to severe readout artifacts and fundamentally restricting its application to low-frame-rate snapshot imaging. Moreover, the deterministic link between event timing and scene intensity offers no inherent privacy protection, as the raw event stream remains visually interpretable. Thus, while temporal mapping provides a path to high-fidelity intensity recovery, its synchronous implementation is ill-suited for confidential, high-speed video capture.

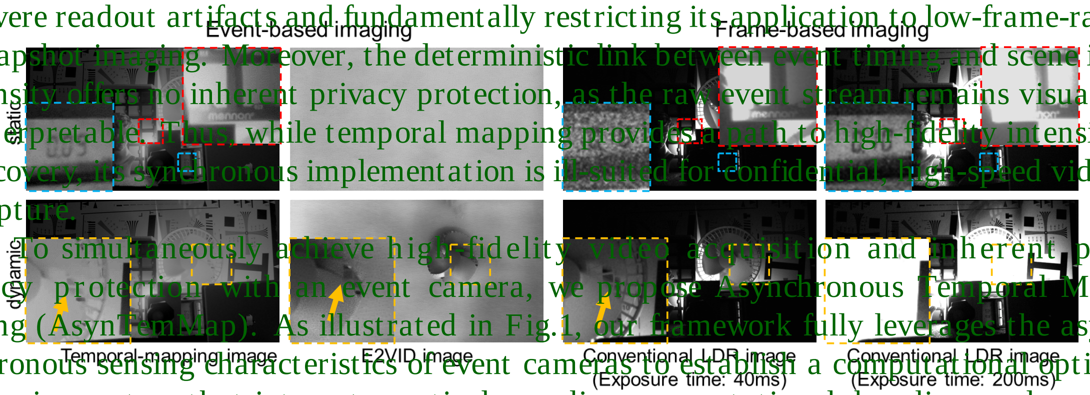

To simultaneously achieve high-fidelity video acquisition and inherent privacy protection with an event camera, we propose Asynchronous Temporal Mapping (AsynTemMap). As illustrated in Fig.1, our framework fully leverages the asynchronous sensing characteristics of event cameras to establish a computational optical imaging system that integrates optical encoding, computational decoding, and neural reconstruction. Specifically, AsynTemMap applies pixel-wise asynchronous transmittance modulation to an event camera, encoding the absolute intensity information of reality into the generation latency Δt^* of each initial positive event (IPE). ...

In summary, our work bridges the gap between high-fidelity intensity recovery and practical high-speed acquisition in event-based vision. The key contributions are: (i) A Paradigm of High-Fidelity Video Acquisition for Event Cameras: We introduce the Asynchronous Temporal Mapping (AsynTemMap) paradigm. By replacing global synchronous modulation with pixel-wise asynchronous modulation, we overcome the bandwidth saturation that plagues existing temporal-mapping meth-

ods, enabling stable, high-frame-rate video acquisition while preserving the ability to faithfully recover absolute scene irradiance. (ii) A Unified Reconstruction Pipeline: We develop an end-to-end pipeline comprising computational decoding and a neural reconstruction model (AsynTemRec). This pipeline jointly compensates for the temporal misalignment artifacts induced by asynchronous sampling and various hardware imperfections, delivering high-fidelity video reconstruction across diverse dynamic scenes, including those with extreme illumination. (iii) Hardware-Level Confidential Vision: We demonstrate that the asynchronous modulation mechanism inherently implements a Secure Event Coding via Asynchronous Modulation (SECAM) scheme. This provides robust, zero-latency privacy protection at the point of capture, with security metrics rivaling software encryption but at a fraction of the computational cost, realizing a truly confidential eye.

III Minor Comments of Reviewer 1

III.1 Clarification of Fig. 2

Reviewer 1: Can you clarify if the temporal-mapping image of Fig. 2 is synchronous or asynchronous?

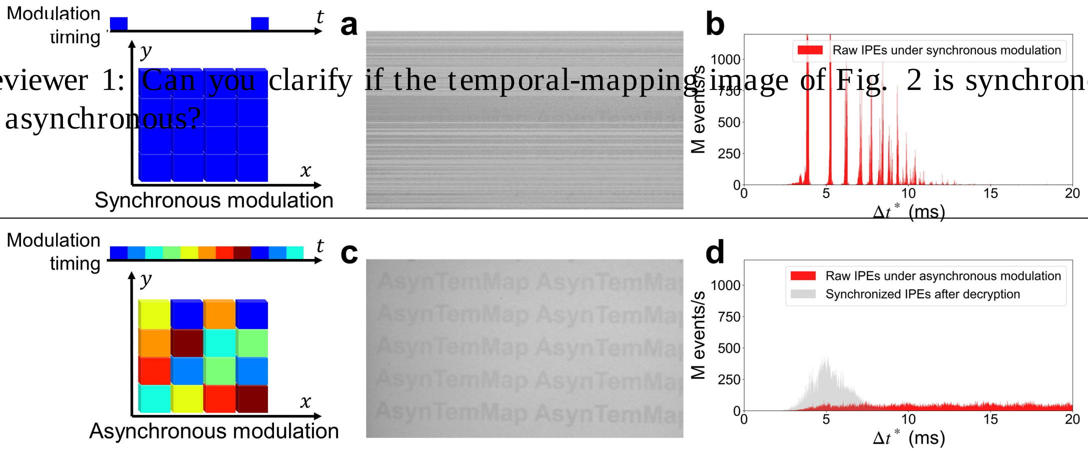

Figure 2: ... In static scenes, temporal-mapping imaging under synchronous modulation captures the brightly lit text ”mennon” on the color chart (red box) alongside the dimly lit digital reading ”0.09” on the illuminance meter (blue box). ...

We thank the reviewer for pointing out the ambiguity in Fig. 2. In the revised manuscript, we have explicitly clarified in the figure caption that the temporal-mapping result shown in Fig. 2 is obtained under synchronous modulation. This example is used to illustrate the inherent high-dynamic-range (HDR) property of temporal-mapping imaging, which applies to both the synchronous temporal-mapping framework (EvTemMap) and the proposed asynchronous temporal-mapping scheme (AsynTemMap).

III.2 Color Consistency in Fig. 3

Comments

Reviewer 1: In subfigure b and d of the Fig. 3, I suggest to use the same color to represent the same metric.

Figure 3: . . . b Under synchronous modulation, IPE timestamp distribution (red) shows event bursts and abnormal spikes. . . . d Under asynchronous modulation, the raw IPE distribution (red) is temporally uniform, and the synchronized IPEs after decryption (gray) match the expected response latency distribution.

Response

In the revised manuscript, we have updated the color coding to ensure that the same metric is represented consistently across subfigures. Specifically, the intensity-encoded events (IPEs) obtained after modulation are now marked in red in both subfigures (b) and (d), while the IPEs recovered after post-processing and decryption in subfigure (d) are shown in gray.

III.3 DMD Pattern Update and Bandwidth Considerations

Comments

Reviewer 1: This work uses a DMD to modulate the imaging process. As DMD patterns are updated globally, does this introduce bandwidth issues like the event camera?

We thank the reviewer for this question. In the proposed system, the DMD modulation employs a fixed sequence of patterns that is preloaded and stored internally in the DMD. During operation, these patterns are played back cyclically according to a predefined modulation period, without requiring continuous pattern updates. As a result, the DMD modulation does not introduce bandwidth issues analogous to those of the event camera.

We note that in Section 3.4.4 (Discussion on chosen-plaintext attacker), we discuss an engineering-level mitigation strategy for chosen-plaintext attacks that involves periodically changing the offset map $O'(x,y)$, which indeed requires reloading DMD patterns. However, this process does not pose a bandwidth bottleneck in practice. During pattern reloading, event acquisition is temporarily paused, and the resulting idle interval depends on the specific loading mechanism but is typically short. Therefore, occasional pattern updates for security purposes do not introduce sustained bandwidth constraints to the imaging system.

III.4 Training Protocol of Neural Reconstruction Baselines

Comments

Reviewer 1: In the neural reconstruction section, I suggest to clarify if the ProPainter is finetuned on the capturing dataset, otherwise it is unfair to compare with a training method.

Response

We thank the reviewer for this important comment regarding the fairness of the neural reconstruction comparison. In the revised manuscript, we have explicitly clarified the training protocols of all reconstruction methods in Section 3.2.

Added

3.2. Neural Reconstruction for Faithful Reality Recovery

...Implementation details. AsynTemRec is trained from scratch on a synthetic dataset generated using the same temporal offset key $O'(x,y)$ as employed in the real test sequences. Details of the synthetic dataset generation, along with the network

architecture, loss function, and training schedule, are provided in Section 3 of the Supplement. To ensure a fair comparison, ProPainter is retrained on the same training dataset (including the identical temporal offset keys $O'(x, y)$) used for AsynTemRec, following its publicly available training schedule. ...

III.5 Validity of the E2VID Baseline under No-Modulation Setting

Comments

Reviewer 1: For the E2VID baseline, it isn't suitable to use the "on" state to act as no-modulation because there will be hardware imperfections (mentioned by the authors).

Response

We thank the reviewer for raising this concern regarding the validity of the E2VID baseline. In the revised manuscript, we have clarified the experimental setup to explain why fixing the DMD micromirrors in the "on" state is appropriate for capturing motion events without modulation.

Specifically, when all micromirrors are fixed in the "on" state, the DMD effectively behaves as a static planar mirror, and the temporal modulation is completely disabled. Under this configuration, modulation-induced hardware artifacts—such as the crack-like patterns caused by micromirror switching—are eliminated. As a result, the captured motion events are free from modulation-specific imperfections and are suitable for use with E2VID, which is designed for standard, unmodulated event streams.

This clarification has been added to the manuscript.

Revised

3.2. Neural Reconstruction for Faithful Reality Recovery

Experimental setup. ...In parallel, for event-based baseline comparison, we capture motion events for each sequence by fixing the DMD micromirrors in the 'on" state, thereby disabling modulation, which turns the DMD into a static planar mirror, eliminating modulation-specific defects such as the crack-like patterns. ...This experimental setup ensures that both the AsynTemMap and the motion-event-reconstruction pipelines share identical optical conditions, including aberrations, thus enabling a direct and equitable comparison of their reconstruction performance.

III.6 Comparison with a Newer Motion-Event-Reconstruction Baseline

Comments

Reviewer 1: It would be better to compare with a newer baseline rather than E2VID, and provide more video visualizations in the result section.

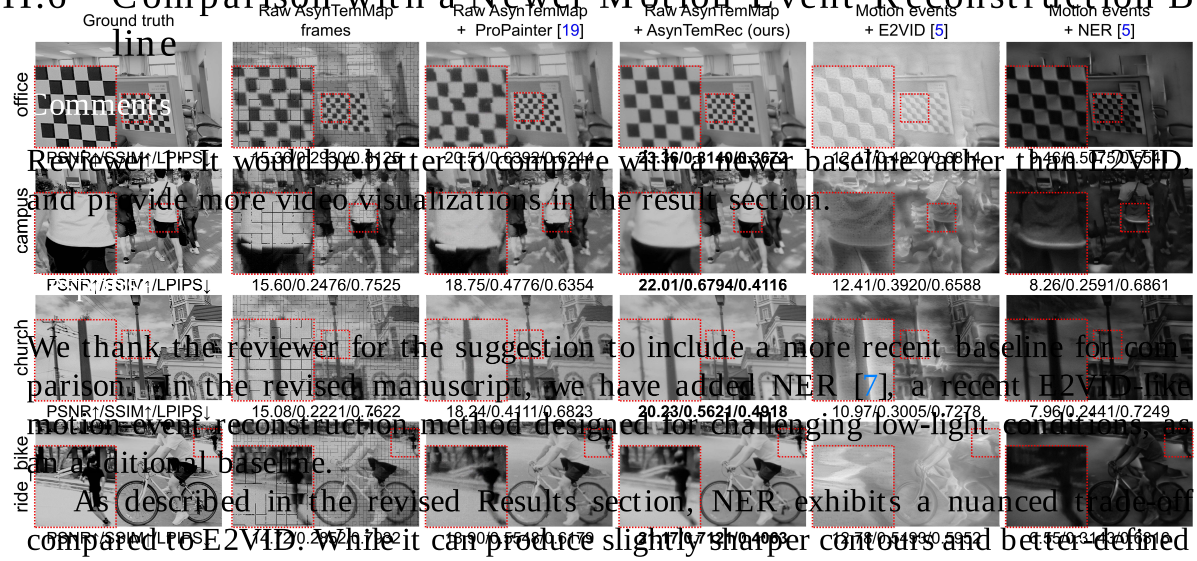

We thank the reviewer for the suggestion to include a more recent baseline for comparison. In the revised manuscript, we have added NER [7], a recent E2VID-like motion-event reconstruction method designed for challenging low-light conditions, as an additional baseline.

As described in the revised Results section, NER exhibits a nuanced trade-off compared to E2VID. While it can produce slightly sharper contours and better-defined textures in some structured regions, this improvement comes at the expense of more severe photometric distortion. In particular, NER's reconstructions are consistently darker and show larger intensity inaccuracies across all evaluated scenes. Consequently, its overall quantitative performance is comparable to, or in some cases worse than, that of E2VID. Quantitative results are summarized in Table 1.

In addition, to address the request for more video visualizations, we have included an extra video sequence (ride_bike) in the qualitative results. This new example further illustrates the temporal reconstruction behavior of the compared methods under dynamic motion conditions.

3.2. Neural Reconstruction for Faithful Reality Recovery

... The ride_bike scene features a camera tracking a nearby cyclist moving at a moderate speed, while a distant runner in the upper-right corner exhibits much faster relative motion. In the raw AsynTemMap frames, this runner region in the red box shows pronounced ASA. Comparing the reconstructions for raw AsynTemMap frames, AsynTemRec effectively corrects this misalignment, whereas ProPainter merely inpaints the crack-like defects without resolving the structural distortion. E2VID, reconstructing solely from motion events, only recovers the foreground cyclist; the contour of the distant runner is severely distorted. For a more comprehensive evaluation, we also include the recent NER [7] as an additional motion-event-reconstruction baseline, which is specifically designed for challenging low-light conditions similar to our experimental setup. Qualitatively, NER shows a nuanced trade-off compared to E2VID: it produces slightly sharper contours and better-defined textures in some structured regions (e.g., the checkerboard in office and the wire poles in church). However, this comes at the cost of more severe photometric distortion; NER's reconstructions are consistently darker and exhibit greater intensity inaccuracy across all scenes. As visually evident

Figure 4: Qualitative and quantitative comparison of reconstruction performance under motion and hardware degradation. Columns (left to right): ground-truth images; raw AsynTemMap frames, exhibiting asynchronous sampling artifacts (ASA) and hardware defects; results after applying ProPainter [8] to inpaint crack-like defects; results after applying proposed AsynTemRec, compensating for both ASA and hardware degradation; E2VID [3] reconstructions from motion events; and NER [7] reconstructions from motion events. Rows (top to bottom): office—a well-texture scene featuring fast linear motion in the foreground against a static background; campus—a complex dynamic scene involving non-linear human motion; church—a fast-motion scene featuring multi-scale structures; and ride_bike—a scene with humans in different movements near and far. The proposed AsynTemRec achieves the highest PSNR/SSIM and lowest LPIPS in all cases.

in the examples of Fig. 4, this trade-off results in NER's overall reconstruction metrics being comparable to, or even lower than, that of E2VID. Critically, both E2VID and NER exhibit a common trait of motion-event-reconstruction methods: they cannot achieve true photometric fidelity.

III.7 Consistency of Encryption Keys in Training and Testing

Reviewer 1: Please clarify if the network is trained and tested with the dataset consist of the same key or different key.

Table 1: Quantitative comparison across methods on all test sequences. This table reports a representative subset of methods from Table 1 for clarity, focusing on the proposed AsynTemRec and two motion-event-based baselines (E2VID and NER). Higher is better for PSNR/SSIM; lower is better for LPIPS. Best results are highlighted in bold.

Sequence	Raw AsynTemMap + AsynTemRec (ours)			Motion events + E2VID [3]			Motion events + NER [7]		
	PSNR↑	SSIM↑	LPIPS↓	PSNR↑	SSIM↑	LPIPS↓	PSNR↑	SSIM↑	LPIPS↓
drone_lake	23.96	0.6607	0.3833	5.89	0.1040	0.8427	14.95	0.2372	0.6049
alley_1	20.58	0.6114	0.4574	12.11	0.3715	0.6840	8.56	0.3052	0.6622
alley_2	20.37	0.6125	0.4442	11.26	0.3683	0.7069	8.04	0.3083	0.6446
catch_ball	22.33	0.5625	0.4174	10.08	0.3345	0.5822	9.13	0.2957	0.5810
ride_bike	21.15	0.6920	0.4044	12.40	0.5201	0.5742	6.53	0.3103	0.6828
campus	21.48	0.6443	0.4264	12.28	0.3539	0.6465	8.32	0.2578	0.6908
shrub	19.49	0.4855	0.4983	11.26	0.2136	0.6421	7.98	0.1603	0.7279
church	19.96	0.5506	0.4932	11.79	0.3109	0.7207	7.82	0.2214	0.7317
office	23.48	0.8103	0.3749	11.98	0.4795	0.7010	9.58	0.5003	0.5749
traffic	21.44	0.5924	0.4136	11.80	0.2729	0.6734	9.36	0.2388	0.6463
Average	21.42	0.6222	0.4313	11.09	0.3329	0.6774	9.03	0.2835	0.6547

Response

We thank the reviewer for requesting this clarification. In the revised manuscript, we have explicitly stated in the implementation details that AsynTemRec is trained and tested using datasets generated with the same temporal offset key $O'(x, y)$.

Specifically, the synthetic training dataset is generated using the identical key $O'(x, y)$ as that employed in the real test sequences. This setting reflects the intended deployment scenario, where a fixed key is used during a capture session, and ensures consistency between training and testing.

Added

3.2 . Neural Reconstruction for Faithful Reality Recovery

...Implementation details. AsynTemRec is trained from scratch on a synthetic dataset generated using the same temporal offset key $O'(x, y)$ as employed in the real test sequences. ...

III.8 Faithfulness of Neural Reconstruction Results

Reviewer 1: As the authors claim that temporal misalignment error and hardware imperfections are solved by the neural reconstruction, is it really a faithful image? If that is, what makes the E2VID reconstruction not a faithful image?

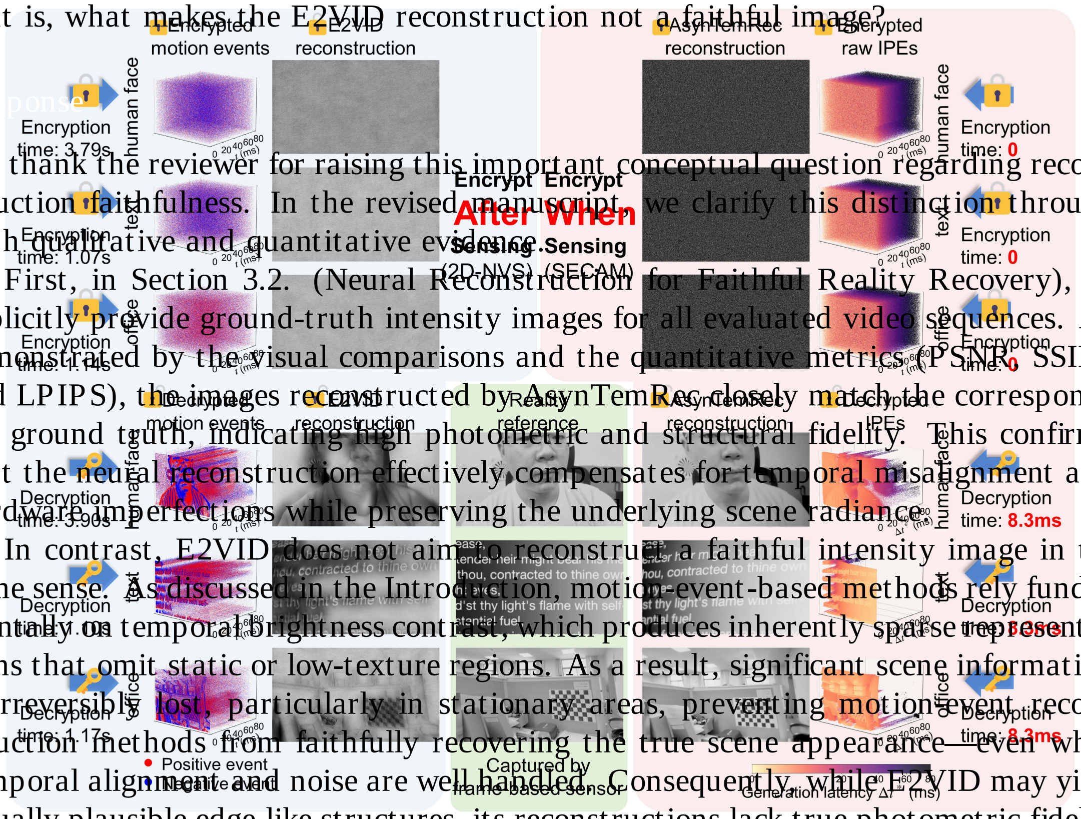

ponse

We thank the reviewer for raising this important conceptual question regarding reconstruction faithfulness. In the revised manuscript, we clarify this distinction through both qualitative and quantitative evidence.

First, in Section 3.2. (Neural Reconstruction for Faithful Reality Recovery), we explicitly provide ground-truth intensity images for all evaluated video sequences. As demonstrated by the visual comparisons and the quantitative metrics (PSNR, SSIM, and LPIPS), the images reconstructed by AsynTemRec closely match the corresponding ground truth, indicating high photometric and structural fidelity. This confirms that the neural reconstruction effectively compensates for temporal misalignment and hardware imperfections while preserving the underlying scene radiance.

In contrast, E2VID does not aim to reconstruct a faithful intensity image in the same sense. As discussed in the Introduction, motion-event-based methods rely fundamentally on temporal brightness contrast, which produces inherently sparse representations that omit static or low-texture regions. As a result, significant scene information is irreversibly lost, particularly in stationary areas, preventing motion-event reconstruction methods from faithfully recovering the true scene appearance—even when temporal alignment and noise are well handled. Consequently, while E2VID may yield visually plausible edge-like structures, its reconstructions lack true photometric fidelity and cannot be considered faithful representations of the original scene.

III.9 Ground-Truth Reference in Fig. 5

Reviewer 1: Please provide the ground truth for Fig. 5.

We thank the reviewer for this suggestion. In the revised manuscript, we have substantially updated Fig. 5 by adding ground-truth scene references for three representative sequences, which are displayed in the central green box of the figure.

These reference images are captured using a frame-based camera after the event acquisition and therefore serve as visual references only; they are not spatially aligned with the event-based reconstructions. We have explicitly clarified this in the figure caption. Despite this limitation, visual comparison clearly shows that the results

Figure 5: Comparison of encryption paradigms for event-based vision. Left (blue panel): Post-sensing software encryption (2D-NVS). The raw motion events are encrypted after acquisition, and the ciphertext/plaintext is reconstructed with E2VID [3]. While visual obfuscation is achieved, this method incurs a high encryption and decryption latency (> 1 s for 80 ms of events). Right (red panel): In-sensing hardware encryption (SECAM). The scene is optically encrypted into chaotic raw IPEs with zero latency during acquisition. Decrypting a frame takes only 8.3 ms; subsequent reconstruction with AsynTemRec faithfully restores the scene. Bottom center (green panel): Scene reference captured by a conventional frame-based sensor (for visual reference only, not spatially aligned). Three representative privacy-sensitive scenes are shown: human face, text, and office. Both encryption paradigms provide visual obfuscation, but SECAM simultaneously offers near-zero-latency hardware encryption and higher-fidelity reconstruction.

IV Additional Comments from Reviewer 2

IV.1 Relation to Optical Encryption in Computational Imaging

Reviewer 2: In fact, the optical encryption property is a common feature in many computational imaging paradigms. This is because, during the intermediate process of optical encoding, it is often visually inaccessible. For instance, in the framework of DMD-based single-pixel imaging, only one-dimensional time fluctuation signals will be obtained after optical encoding. Without knowing the optical encoding (or illumination mode), it is impossible to obtain the spatial information of the target.

Response

We thank the reviewer for this insightful observation. We fully agree that optical encryption is a common implicit property of many computational imaging paradigms, such as DMD-based single-pixel imaging, where intermediate optical measurements are visually inaccessible without knowledge of the encoding pattern.

In the revised manuscript, we clarify that SECAM does not claim to be the first optical encryption method in a general sense. Instead, its key distinction lies in being a hardware-level encryption paradigm natively designed for event-based vision. Unlike frame-based computational imaging approaches that impose encryption as a separate optical encoding stage, SECAM intrinsically exploits the asynchronous sensing principle of event cameras. Specifically, encryption is achieved by randomizing the per-pixel modulation onset within the same optical encoding process that enables high-fidelity video acquisition.

This co-design tightly couples sensing, encoding, and encryption: privacy protection incurs no additional system complexity or latency, and is achieved simultaneously with faithful video capture. We have added a dedicated discussion in the manuscript to emphasize this distinction and to position SECAM relative to existing optical encryption paradigms.

Added

3.3 . Confidential Vision through Hardware-Level Encryption

...

SECAM establishes a hardware-level encryption paradigm natively designed for event-based vision. While optical encryption concepts exist for frame-based systems (e.g., single-pixel imaging), SECAM is not a mere adaptation of such methods. Instead, it intrinsically exploits the asynchronous sensing principle of event cameras: encryption is achieved by randomizing the per-pixel modulation onset within the same optical encoding process designed for high-fidelity video acquisition. This co-design means that privacy protection incurs no additional system complexity—the encryption is

accomplished simultaneously with faithful video capture, resulting in the zero-latency advantage demonstrated in Table 2.. . .

IV.2 Inference Time of Neural Reconstruction

Comments

Reviewer 2: There is no doubt that the time cost for encryption and decryption is extremely low. The time cost of neural reconstruction process should be clearly stated in the main text.

Response

We thank the reviewer for this helpful suggestion. In the revised manuscript, we have explicitly added a dedicated Inference speed paragraph in the main text to report the computational cost of the neural reconstruction stage.

Added

3.2. Neural Reconstruction for Faithful Reality Recovery

Inference speed. For practical deployment, we measure the inference speed of AsynTemRec on an NVIDIA RTX 4090 GPU with FP32 precision. AsynTemRec processes an input frame of size 1280×720 in 198.82 ms on average, corresponding to approximately 5 frames per second. This speed is acceptable for offline, high-quality reconstruction applications where reconstruction fidelity is prioritized over real-time performance.

V Additional Comments from Reviewer 3

V.1 Comparison with Existing Event-Camera Encryption Schemes

Comments

Reviewer 3: The main text mentions that conventional event streams leak contour information, but it does not provide a deep comparison between SECAM and existing hardware or algorithmic encryption schemes specifically designed for event cameras.

Response

We thank the reviewer for this comment. In the revised manuscript, we have significantly strengthened the comparison between SECAM and existing event-camera encryption approaches.

Specifically, we include visual and quantitative comparisons with a representative

event-domain encryption method, 2D-NVS, which is explicitly designed for encrypting motion-event streams. Figure 5 has been updated to include the corresponding ciphertext visualizations of existing event encryption algorithm (2D-NVS [1]). In addition, the added Sections 3.4.2 and 3.4.3 now provide a detailed security analysis comparing SECAM and 2D-NVS under ciphertext-only and known-plaintext attack models.

Details of the corresponding modifications and analyses are provided in Response Section I.2 and Response Section I.3.

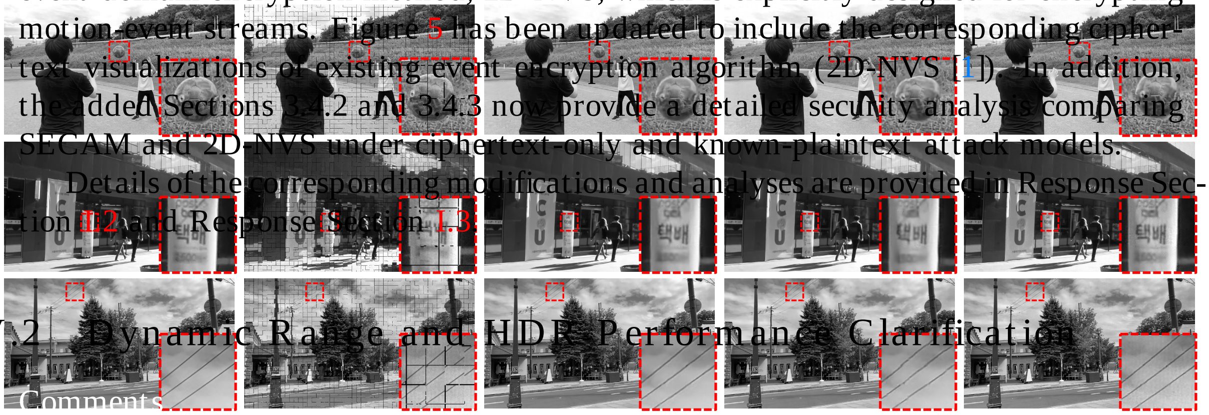

V.2 Dynamic Range and HDR Performance Clarification

Comments

Reviewer 3: Only a theoretical value of 114 dB is given. Quantitative comparison data on measured dynamic range and signal-to-noise ratio (SNR) between AsynTemMap and existing event-camera HDR imaging methods (e.g., [5]) in standard HDR scenes needs to be added.

We thank the reviewer for this comment. In the revised manuscript, we have supplemented the theoretical analysis with a measured dynamic range result. Specifically, under the experimental setting shown in Fig. 2, we report a measured dynamic range of 115 dB.

We would like to clarify, however, that high-dynamic-range (HDR) imaging itself is not claimed as an incremental contribution of AsynTemMap over our prior work EvTemMap. As stated in the revised manuscript, faithful HDR reconstruction is a core contribution of EvTemMap, and AsynTemMap is designed to inherit this capability rather than fundamentally advance HDR reconstruction quality beyond existing event-based HDR methods (e.g., [5]). Accordingly, AsynTemMap is not positioned as a new HDR benchmark, and exhaustive quantitative comparisons of dynamic range or SNR against prior HDR-focused methods are outside the primary scope of this work.

The central contribution of this paper lies instead in upgrading temporal-mapping imaging from a snapshot-only paradigm (EvTemMap) to a video-capable framework (AsynTemMap), thereby overcoming bandwidth limitations under synchronous modulation, and in leveraging this system to realize hardware-level visual encryption (SECAM). We have revised the manuscript to clearly reflect this positioning and contribution scope, as detailed in Response Section II.

V.3 Limited Recovery of Fine, Low-Contrast Features in the “Church” Scene

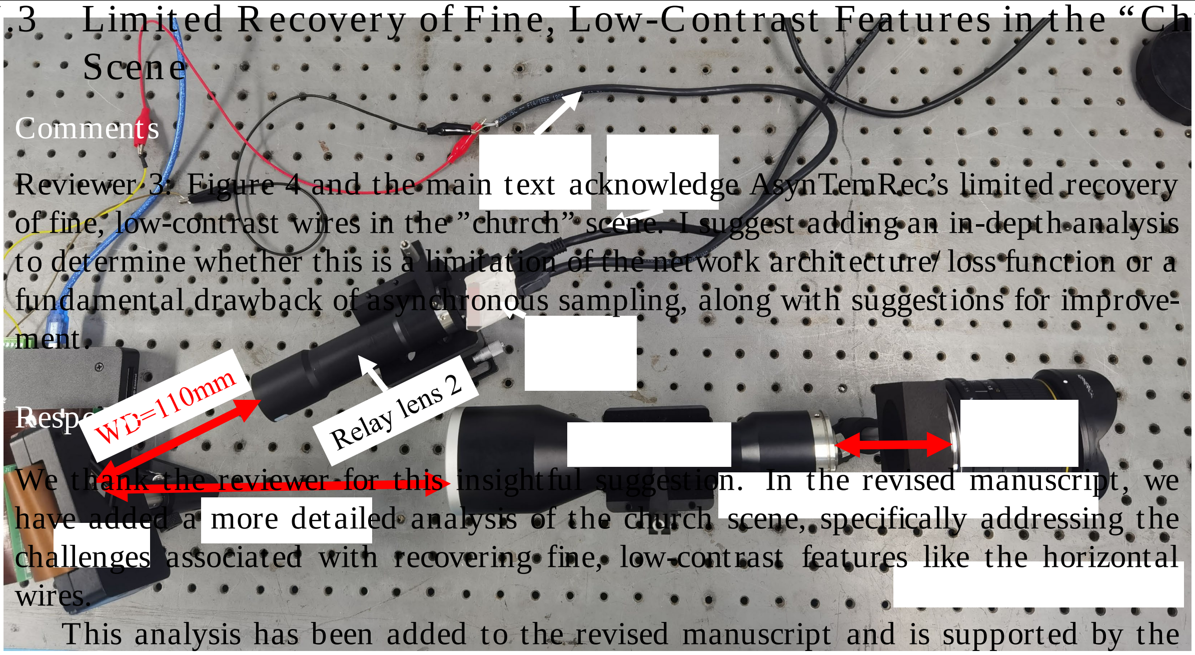

Comments

Reviewer 3: Figure 4 and the main text acknowledge AsynTemRec's limited recovery of fine, low-contrast wires in the ”church” scene. I suggest adding an in-depth analysis to determine whether this is a limitation of the network architecture/ loss function or a fundamental drawback of asynchronous sampling, along with suggestions for improvement.

Response

We thank the reviewer for this insightful suggestion. In the revised manuscript, we have added a more detailed analysis of the church scene, specifically addressing the challenges associated with recovering fine, low-contrast features like the horizontal wires.

This analysis has been added to the revised manuscript and is supported by the experimental results presented in Fig. S5. Assuming that the optical aberrations are well-corrected, the raw frames captured by the AsynTemMap system should show fine lines similar to those shown in the “Raw AsynTemMap” in Fig. S5. While these fine lines are misaligned, they remain visible. After reconstruction with AsynTemRec(full), the fine details of these lines are effectively restored.

3.2 . Neural Reconstruction for Faithful Reality Recovery

...The church scene, containing both coarse and fine structures in motion, presents significant challenges for scene reconstruction. In the raw AsynTemMap frames, the finest horizontal wires exhibit inherently low contrast due to optical aberrations in the system. Both ProPainter and AsynTemRec manage to recover the basic structure of these wires, with AsynTemRec providing a cleaner result by effectively suppressing noise. However, neither method significantly enhances the contrast of these subtle textures. We attribute this limitation primarily to the optical system's aberrations, which attenuate the signal of fine, low-contrast structures in the raw AsynTemMap frames before reconstruction. This attribution is directly supported by a simulation experiment: when AsynTemRec is tested on a synthetic church sequence free from optical aberrations (see Fig. S5 in the Supplement), fine features like the wires are recovered with high fidelity. Therefore, the most effective strategy to enhance the recovery of such fine details is to improve the optical system. A secondary factor is the training objective: the pixel-wise MSE loss used in AsynTemRec prioritizes overall fidelity and naturally yields smooth outputs, but does not specifically amplify weak signals. For tasks requiring enhanced detail, supplementing the perceptual or adversarial loss could be beneficial, though may introduce non-physical artifacts. ...

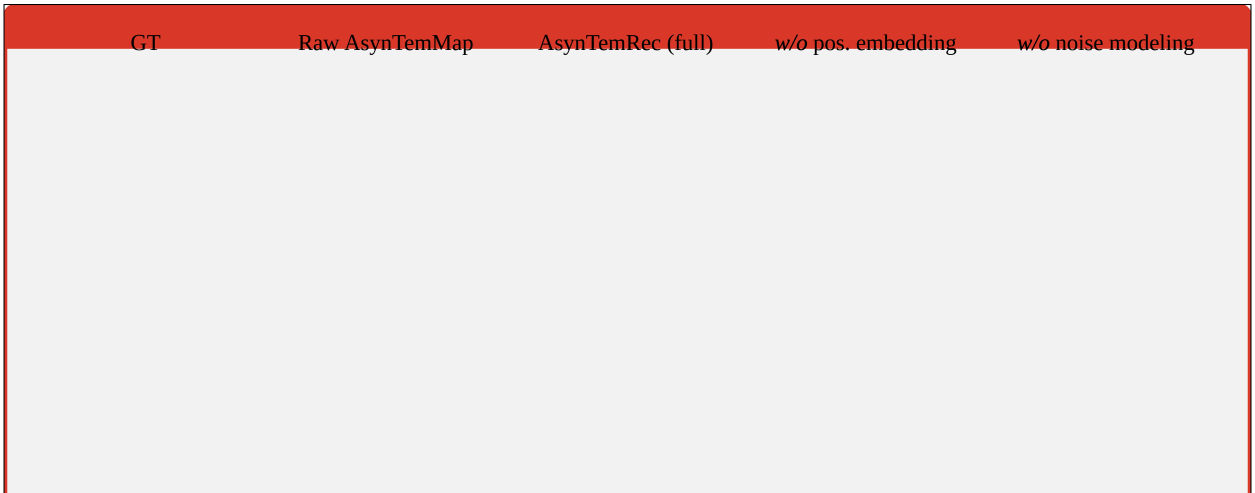

Figure S5: Qualitative ablation study on the synthetic dataset. Red boxes highlight representative regions of interest. Raw AsynTemMap frames exhibit pronounced hardware-induced degradations and Asynchronous Sampling Artifacts (ASA). The full AsynTemRec model effectively mitigates both effects, producing high-quality reconstructions. Removing positional embeddings results in noticeable feature deformation in regions affected by ASA, while omitting noise modeling leads to reconstructions that retain considerable artifacts and spurious textures.

V.4 Labeling Key Distances or Focal Lengths in the Hardware Schematic

Reviewer 3: In the hardware schematic, consider labeling key distances or focal lengths of optical components.

We thank the reviewer for the suggestion. In the revised supplemental document, we have added the key distances and focal lengths of optical components to the hardware schematic in Fig. S1 for clarity.

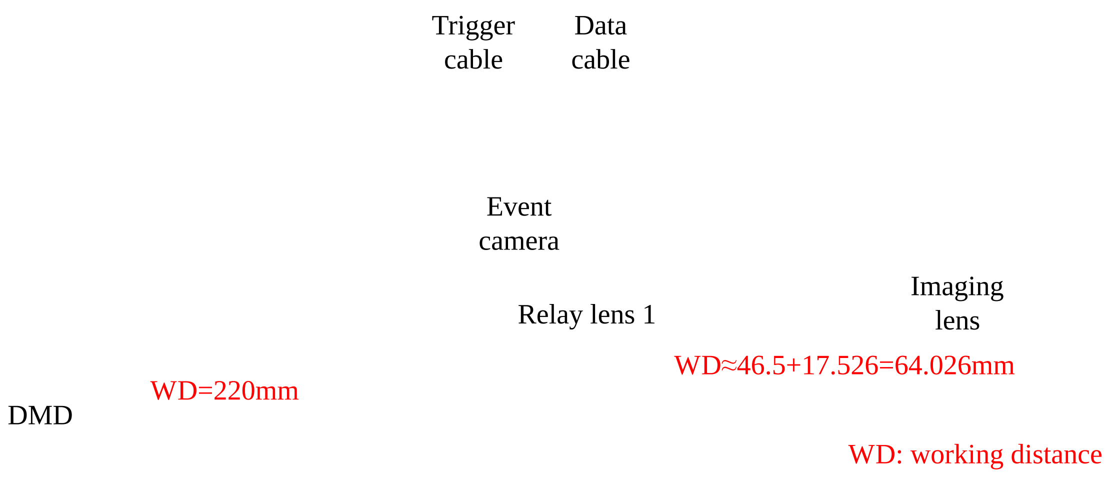

Figure S1: Hardware Setup for the AsynTemMap System. The hardware setup consists of a Digital Micromirror Device (DMD), an event camera, two relay lenses, and an imaging lens. The image planes of the imaging lens, DMD, and event camera are conjugated through the two relay lenses. The event camera is connected to the DMD via a trigger cable to record the precise timestamps of DMD pattern switches. Key optical distances are labeled in red. The working distance between the imaging lens and Relay Lens 1, as labeled, is the sum of their respective back focal lengths (46.5 mm for the imaging lens and 17.526 mm for Relay Lens 1).

V.5 Clarification of Calibration Steps and Uniform Illumination Assumptions

Reviewer 3: Clarify the dependence of some calibration steps on uniform illumination assumptions and their applicable conditions.

We thank the reviewer for this comment. In the revised supplemental document, we have clarified the dependence of certain calibration steps on uniform illumination assumptions and outlined their applicable conditions. Specifically, we have made the following clarifications.

Supp Section 1.3. Calibration of Event Camera and DMD Coordinates

...Global, low-frequency non-uniformity in illumination is acceptable, as it does not affect the local extraction of checkerboard corners. ...

Supp Section 2.3. Vignetting and FPN Texture

...This calibration requires a high-quality, spatially uniform Lambertian source (e.g., an integrating sphere) to establish a reliable reference for the system's intrinsic photometric response. It is a one-time procedure that characterizes fixed optical and DMD properties; the stringent uniformity condition is therefore both necessary and acceptable for obtaining an accurate degradation model. ...

Supp Section 2.4. Micromirror-Jitter-Induced Dropouts

...This calibration requires the illumination to be sufficiently uniform such that the latency variation induced by illumination non-uniformity is substantially smaller than the deviation caused by micromirror jitter. This ensures that the mask $M(x)$ accurately captures the hardware defect rather than being contaminated by scene-dependent lighting artifacts. ...

References

[1] Bowen Du, Weiqi Li, Zeju Wang, Manxin Xu, Tianchen Gao, Jiajie Li, and Hongkai Wen. Event encryption for neuromorphic vision sensors: Framework, algorithm, and evaluation. Sensors, 21(13):4320, 2021.

[2] Tesseract OCR Developers. Tesseract ocr (version 5.5.0.20241111). https://github.com/tesseract-ocr/tesseract/releases/tag/5.5.0, 2024. Accessed: 2025-05-15.

[3] Henri Rebecq, René Ranftl, Vladlen Koltun, and Davide Scaramuzza. High speed and high dynamic range video with an event camera. IEEE Trans. Pattern Anal. Mach. Intell. (T-PAMI), 2019.

[4] Morris Dworkin. Recommendation for block cipher modes of operation: Methods and techniques. NIST Special Publication 800-38A, National Institute of Standards and Technology, December 2001.

[5] Yuhan Bao, Lei Sun, Yuqin Ma, and Kaiwei Wang. Temporal-mapping photography for event cameras. In European Conference on Computer Vision, pages 55–72. Springer, 2025.

[6] Yuliang Wu, Ganchao Tan, Jinze Chen, Wei Zhai, Yang Cao, and Zheng-Jun Zha. Event-based asynchronous hdr imaging by temporal incident light modulation. Optics Express, 32(11):18527–18538, 2024.

[7] Haoyue Liu, Jinghan Xu, Shihan Peng, Yi Chang, Hanyu Zhou, Yuxing Duan, Lin Zhu, Yonghong Tian, and Luxin Yan. Ner-net+: Seeing motion at nighttime with an event camera. IEEE Transactions on Pattern Analysis and Machine Intelligence, 2025.

[8] Shangchen Zhou, Chongyi Li, Kelvin CK Chan, and Chen Change Loy. Propainter: Improving propagation and transformer for video inpainting. In Proceedings of the IEEE/CVF international conference on computer vision, pages 10477–10486, 2023.

Author responses for COMMSENG-25-0908-A
Capture Reality through a Confidential Eye

Yuhan Bao, Hao Shi, Jiajun Zhai , and Kaiwei Wang

For the minor comments to the revised version by the reviewer # 2, we made the following modifications.

The revision reports AsynTemRec inference speed (198.82 ms/frame = 5 fps on RTX 4090) and frames it as offline, high-quality reconstruction. However, wording such as "real-time privacy-preserving vision / strict real-time performance" may be interpreted as end-to-end real-time reconstruction. Please explicitly separate real-time encryption/decryption/decoding from non-real-time neural reconstruction, and keep the abstract/conclusion consistent.

As required, we clarified the discussion on "real-time" in the final conclusion of the introduction and discussion. In abstract, we do not mention "real-time" related statements.

1. Introduction

...

Unlike traditional software-based event encryption algorithms, AsynTemMap eliminates encryption overhead and reduces decryption time by a factor of 200, thereby ensuring real-time privacy-preserving data acquisition at the point of capture, while subsequent neural reconstruction is performed offline for high-quality recovery.

...

4. Discussion

...

In the current implementation, real-time encryption and decoding are complemented by offline high-quality reconstruction, enabling efficient data acquisition and

high-fidelity recovery.

. . .

Please add a one-sentence clarification (main text or supplement): whether 115 dB refers to the scene illuminance dynamic range (measured by lux meter) or the reconstructable system dynamic range (derived from noise/threshold/temporal resolution). The current wording reads closer to the former.

According to the suggestion, we clarified that 115dB refers to the dynamic range of the real scene.

To evaluate imaging performance under extreme illumination and motion, we construct a laboratory scene under an extremely dark ambient condition (0.1 Lux on average), which contains local illumination ranging from 28,400 Lux (brightest) to 0.05 Lux (darkest), corresponding to a real scene illuminance dynamic range of 115dB, and incorporate a rotating fan to illustrate dynamic distortion. The comparative results of different imaging methods under static and dynamic conditions are presented in Fig.2.
